# Exceptionally high charge mobility in phthalocyanine-based poly(benzimidazobenzophenanthroline)-ladder-type two-dimensional conjugated polymers

**DOI:** 10.1038/s41563-023-01581-6

**Published:** 2023-06-19

**Authors:** Mingchao Wang, Shuai Fu, Petko Petkov, Yubin Fu, Zhitao Zhang, Yannan Liu, Ji Ma, Guangbo Chen, Sai Manoj Gali, Lei Gao, Yang Lu, Silvia Paasch, Haixia Zhong, Hans-Peter Steinrück, Enrique Cánovas, Eike Brunner, David Beljonne, Mischa Bonn, Hai I. Wang, Renhao Dong, Xinliang Feng

**Affiliations:** 1 Center for Advancing Electronics Dresden (cfaed) and Faculty of Chemistry and Food Chemistry, Technische Universität Dresden, Dresden, Germany; 2 Max Planck Institute for Polymer Research, Mainz, Germany; 3 Faculty of Chemistry and Pharmacy, University of Sofia, Sofia, Bulgaria; 4 Max Planck Institute of Microstructure Physics, Halle, Germany; 5 Anhui Province Key Laboratory of Condensed Matter Physics at Extreme Conditions, High Magnetic Field Laboratory, HFIPS, Chinese Academy of Sciences, Hefei, China; 6 Laboratory for Chemistry of Novel Materials, University of Mons, Mons, Belgium; 7 Institute of Physical Chemistry II, Friedrich-Alexander-Universität Erlangen-Nürnberg, Erlangen, Germany; 8 Instituto Madrileño de Estudios Avanzados en Nanociencia (IMDEA Nanociencia), Madrid, Spain; 9 Key Laboratory of Colloid and Interface Chemistry of the Ministry of Education, School of Chemistry and Chemical Engineering, Shandong University, Jinan, China

**Keywords:** Materials science, Chemistry, Physics

## Abstract

Two-dimensional conjugated polymers (2DCPs), composed of multiple strands of linear conjugated polymers with extended in-plane π-conjugation, are emerging crystalline semiconducting polymers for organic (opto)electronics. They are represented by two-dimensional π-conjugated covalent organic frameworks, which typically suffer from poor π-conjugation and thus low charge carrier mobilities. Here we overcome this limitation by demonstrating two semiconducting phthalocyanine-based poly(benzimidazobenzophenanthroline)-ladder-type 2DCPs (2DCP-MPc, with M = Cu or Ni), which are constructed from octaaminophthalocyaninato metal(ii) and naphthalenetetracarboxylic dianhydride by polycondensation under solvothermal conditions. The 2DCP-MPcs exhibit optical bandgaps of ~1.3 eV with highly delocalized π-electrons. Density functional theory calculations unveil strongly dispersive energy bands with small electron–hole reduced effective masses of ~0.15*m*_0_ for the layer-stacked 2DCP-MPcs. Terahertz spectroscopy reveals the band transport of Drude-type free carriers in 2DCP-MPcs with exceptionally high sum mobility of electrons and holes of ~970 cm^2^ V^−1^ s^−1^ at room temperature, surpassing that of the reported linear conjugated polymers and 2DCPs. This work highlights the critical role of effective conjugation in enhancing the charge transport properties of 2DCPs and the great potential of high-mobility 2DCPs for future (opto)electronics.

## Abstract (web summary)

Linear π-conjugated polymers have attracted great attention as semiconductors for (opto)electronic devices, but charge transfer is only effective along polymer chains. Here poly(benzimidazobenzophenanthroline)-ladder-type two-dimensional conjugated polymers are presented with high charge carrier mobilities.

## Main

Linear one-dimensional π-conjugated polymers (1DCPs)^1^ have attracted great interest as semiconductor materials^2^ in (opto)electronic devices such as organic solar cells, organic field-effect transistors and organic photodetectors^3^. In 1DCPs, charge conduction effectively occurs along the polymer chain, whereas the interchain charge transfer sets a bottleneck for charge transport via inefficient hopping^4^. By expanding the dimensionality of conjugated polymers, multiple charge transport strands can be established to bypass potential defects^5^, narrow the bandgap^6^ and suppress vibrational degrees of freedom^7,8^ to facilitate effective band-like transport^8–11^ in the resulting two-dimensional conjugated polymers (2DCPs), leading to enhanced electronic performance^12^ and potentially unique electronic structures (for example, Dirac cones)^13,14^.

In this context, two-dimensional π-conjugated covalent organic frameworks (2D c-COFs)^15–18^ represent a unique class of layer-stacked, crystalline 2DCPs with in-plane π-conjugation, which allow control over the spatial arrangement of molecular building blocks^7,19^. Despite the growing interest in developing 2D c-COFs^20–23^, they typically suffer from poor π-electron delocalization^24^ due to the polarized imine linkage^25–27^, which results in large bandgaps and inefficient charge transport^28^. Recent advances in vinylene-linked 2D c-COFs (or 2D poly(arylene vinylene)s^29–34^) have demonstrated enhanced π-conjugation along with high chemical/thermal stability. Nevertheless, these polymer materials are jet limited by inefficient conjugation, thus exhibiting weak or moderate in-plane dispersion in the energy band diagram^8,11,15,34–37^. Accordingly, the reported intrinsic charge carrier mobilities (without doping treatment^37,38^) remain moderate with values typically below ~20 cm^2^ V^−1^ s^−1^ (refs. ^15,37^).

To increase the effective π-conjugation length, ladder-type conjugated polymers^39,40^ (for example, graphene nanoribbons^41^ and poly(benzimidazobenzophenanthroline) (BBL)^39,40^ with two or more independent but tied strands of bonds) represent a unique molecular geometry, which consists of linearly fused aromatic subunits with strengthened parallel *p*-orbital interactions^42^ (versus single–double bond alternation in polyimines and poly(arylene vinylene)s). Although ladder-type 2DCPs^15,42,43^ can provide independent control over the molecular arrangements to enhance charge conduction, a rational design in their polymer backbones with considerable electronic band dispersion and strong π-electron delocalization to achieve high charge carrier mobility has remained unexplored.

Here we report the first examples of two phthalocyanine-based BBL-ladder-type crystalline 2DCPs (termed as **2DCP-MPc**, where M = Cu or Ni) with unique band transport and outstanding charge carrier mobilities. To optimize the conjugation degree in 2DCPs, we investigate various conjugated linkages by theoretical computations on representative model compounds, and find that the BBL-ladder-type structure exhibits delocalized π-electrons with a narrow highest occupied molecular orbital (HOMO)–lowest unoccupied molecular orbital (LUMO) gap. Thus, we synthesize **2DCP-MPc**s via a *p*-toluenesulfonic acid (PTSA)-modulated polycondensation between octaaminophthalocyaninato metal(ii) and naphthalenetetracarboxylic dianhydride under solvothermal conditions. **2DCP-MPc**s display narrow optical bandgaps of ~1.3 eV. Density functional theory (DFT) calculations reveal strongly dispersive valence and conduction bands and thus a low electron–hole reduced effective charge carrier mass (m*) of 0.137*m*_0_ and 0.172*m*_0_ for the layer-stacked **2DCP-NiPc** and **2DCP-CuPc**, respectively. Terahertz (THz) spectroscopy demonstrates a Drude-type charge transport behaviour with room-temperature scattering times up to 76 fs. The delocalized charge transport, together with the small m*, gives rise to exceptionally high electron–hole sum mobility of up to ~970 cm^2^ V^−1^ s^−1^ in **2DCP-NiPc**, which is comparable to that of crystalline silicon and significantly exceeds those for reported 1DCPs and 2DCPs. Moreover, temperature-dependent THz photoconductivity measurements show negative temperature coefficients of mobility (d*μ*/d*T* < 0) in **2DCP-MPc**s, consistent with phonon-scattering-limited band transport.

## Results

### Design principle of conjugated linkages in 2DCPs

**Fig. 1:**
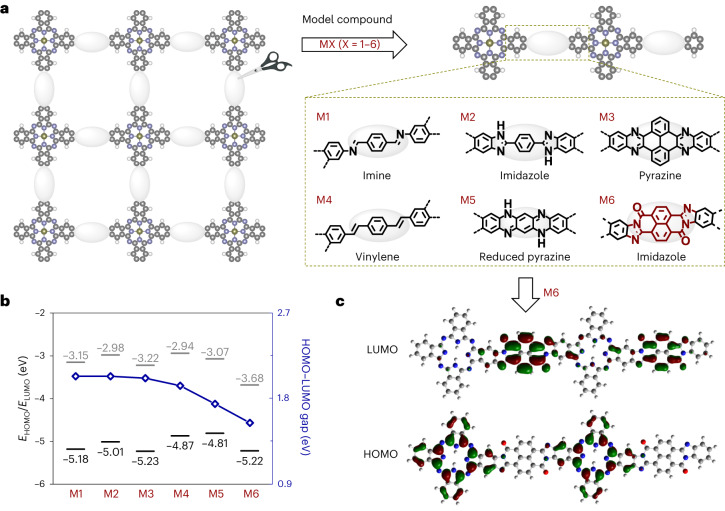
Design principle of phthalocyanine-based 2DCPs. **a**, Schematic showing the phthalocyanine-based 2DCPs connected by different conjugated linkages (in the pale ellipses) and the selected model compounds **MX** (X = 1**−**6). C, grey; N, lavender; H, white. **b**, HOMO**−**LUMO energy levels and gaps of **M1**−**M6**. **c**, Molecular electronic structures of the frontier orbitals of **M6**. Source data

To endow 2DCPs with highly extended π-delocalization, we first perform DFT calculations on various possible conjugated linkages of the photo-/electro-active building block phthalocyanine^15,35^ (the model compounds **MX** (Fig. 1a) are employed to simplify the calculation), such as imine in **M1**, imidazole of 2-phenylimidazole in **M2**, pyrazine in **M3**, vinylene in **M4**, partially reduced pyrazine in **M5** and imidazole of perinone in **M6** (BBL ladder type^39^). Their molecular electronic structures and HOMO–LUMO energy levels are compared in Fig. 1b and Supplementary Figs. 1–6. Despite the slight shift in the HOMO–LUMO energy levels (gap, ~2.02 eV; Fig. 1b), the π-electrons in **M1**−**M3** are localized within the phthalocyanine moiety (Supplementary Figs. 1–3), which suggests ineffective π-conjugation. The vinylene groups in **M4** and reduced pyrazine units in **M5** bond the neighbouring phthalocyanines with delocalized HOMO electrons (Supplementary Figs. 4 and 5), resulting in simultaneously raised HOMO–LUMO energy levels (**M1** as the reference) and diminished energy gaps (down to 1.93 and 1.74 eV, respectively). It is noteworthy that when BBL-type imidazole serves as the linkage, the LUMO level of **M6** considerably decreases by 0.53 eV compared with **M1**, and the HOMO−LUMO gap is narrowed to 1.54 eV. We account for these observations by the favourable intramolecular charge transfer in the linear skeleton (Fig. 1c; note that the isomers of **M6** are energetically less stable and show a negligible change in the electronic structures and HOMO–LUMO energy levels; Supplementary Figs. 6 and 7). We further calculate the electronic structures of the related 1DCPs, and find that the energy levels/gaps follow the same trend as that of the model compounds (Supplementary Figs. 8 and 9). Therefore, we envision that the development of BBL-ladder-type 2DCPs would confer a narrow bandgap and dispersive electronic bands to the 2D frameworks.

### Synthesis and characterizations of 2DCPs

**Fig. 2:**
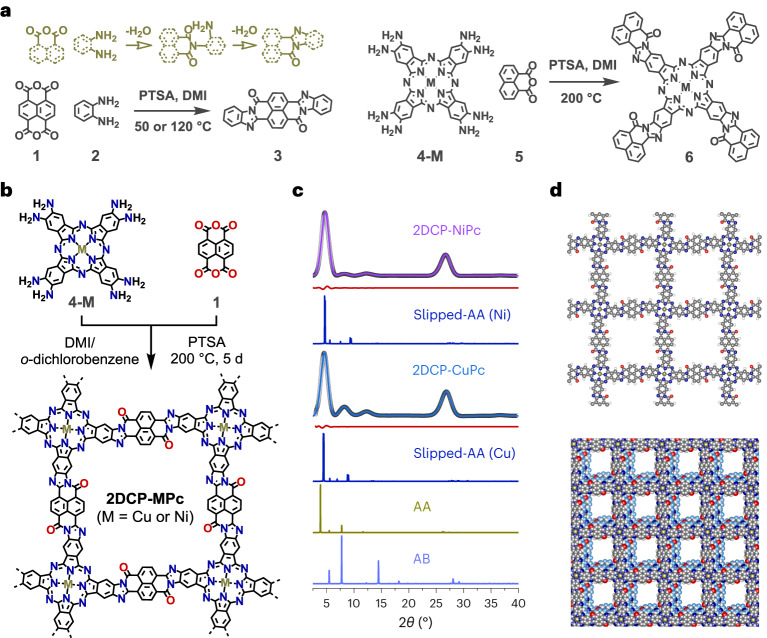
Phthalocyanine-based BBL-ladder-type 2DCP-MPcs. **a**, Schematic of the formation of the imidazole ring and synthesis of model compounds **3** and **6**. **b**, Schematic of the synthesis of **2DCP-MPc**. **c**, Experimental (violet and blue lines), Pawley refined (black dotted lines) and calculated (dark blue lines for slipped AA stacking; Supplementary Information) PXRD patterns as well as their difference plots (dark red lines) of **2DCP-MPc**s. The calculated PXRD patterns of AA- and AB-stacked **2DCP-CuPc** are shown in the dark yellow and light blue lines, respectively. **d**, Models of the monolayer (C, grey; N, dark blue; O, red; Cu, dark yellow; H, white) and slipped-AA-stacked **2DCP-CuPc**. Source data

Due to the harsh conditions (for example, ~200 °C temperature) required for the imidazole ring formation from *o*-diamine and anhydride (Fig. 2a, dark yellow scheme), the synthesis of crystalline BBL-ladder-type 2DCPs has not been realized so far. To achieve the imidazole ring formation under benign conditions, we investigated the reaction mechanism via multiple model reactions (Supplementary Figs. 9–18 and Supplementary Schemes 1−3) and DFT calculations (Supplementary Figs. 19 and 20). The results reveal that an organic acid (that is, PTSA) enhances the reactivity due to its unique protonation–deprotonation ability, compared with basic or catalyst-free conditions (Supplementary Figs. 10 and 19 and Supplementary Scheme 1).

We then synthesized the crystalline phthalocyanine-based BBL-ladder-type **2DCP-MPc** by 2D polycondensation between octaaminophthalocyaninato metal(ii) (**4-M**, M = Cu or Ni) and naphthalenetetracarboxylic dianhydride (**1**) using PTSA as the catalyst and 1,3-dimethyl-2-imidazolidinone (DMI)/*o*-dichlorobenzene (v/v = 2/1) as the solvent at 200 °C for 5 days (Fig. 2b; the model compounds are shown in Fig. 2a and Supplementary Figs. 21 and 22). For **2DCP-CuPc**, a powder X-ray diffraction (PXRD) analysis reveals its crystalline nature with distinct 2*θ* peaks at 4.62°, 8.18°, 12.17° and 26.82° (Fig. 2c, blue line), which can be assigned to the (100), (200), (400) and (002) crystallographic planes, respectively, for a slipped-AA-stacking geometry (Fig. 2c,d; Supplementary Figs. 23–28 provide details and discussion of the isomeric structures). Pawley refinement provides a PXRD pattern matching well with the experimental results, as evidenced by the low *R*_wp_ and *R*_p_ (reliability factors) values of 3.39% and 2.26%, respectively (Supplementary Fig. 29). As expected, **2DCP-NiPc** displays similar PXRD pattern and stacking geometry to **2DCP-CuPc** (Fig. 2c, violet line, and Supplementary Figs. 30 and 31).

Field-emission scanning electron microscopy and transmission electron microscopy images reveal ordered spherical aggregated polycrystals up to ~500 nm in diameter and lattice fringes with parameters of *a* = *b* = ~2.1 nm in **2DCP-MPc**s (Supplementary Figs. 32–35). The Fourier-transform infrared spectra of **2DCP-MPc**s show C=N and C=O stretching vibrations at 1,660 and 1,701 cm^−1^, respectively, indicating the formation of imidazole rings (Supplementary Fig. 36)^44^, which is further confirmed by solid-state nuclear magnetic resonance, Raman and X-ray photoelectron spectroscopies (Supplementary Figs. 37–41; Supplementary Figs. 42–45 provide other characterizations). The intense peaks at 1,582−1,603 cm^−1^ in the Raman spectra (Supplementary Fig. 41) can be assigned to the ring stretching (C=C) pattern, analogous to those G-band peaks in graphene nanoribbons^41^, indicative of efficient conjugation in **2DCP-MPc**s.

### Electronic band structures and effective mass calculation

**Fig. 3:**
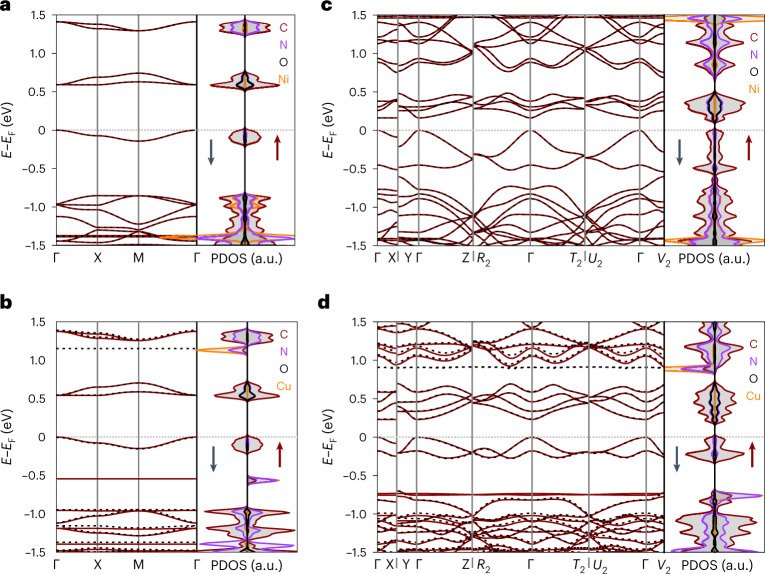
Energy band diagrams of 2DCP-MPcs. **a**,**b**, Electronic band structures and PDOS (C*p*, N*p*, O*p* and Cu/Ni*d*) of **2DCP-NiPc** (**a**) and **2DCP-CuPc** (**b**) monolayers. **c**,**d**, Electronic band structures and PDOS of the slipped-AA-stacked **2DCP-NiPc** (**c**) and **2DCP-CuPc** (**d**). The Fermi energy is shifted to 0 eV. Source data

We calculated the energy band diagrams of the **2DCP-MPc** monolayers and the slipped-AA-stacked structures via DFT/Perdew–Burke–Ernzerhof method. The results suggest that both **2DCP-NiPc** (Fig. 3a) and **2DCP-CuPc** (Fig. 3b) monolayers are direct-bandgap semiconductors with highly dispersive valence band maximum (VBM); two bands are nearly degenerate at the Γ point, forming the conduction band minimum (CBM). Taking the **2DCP-CuPc** monolayer as an example, the dispersed VBM leads to a small averaged hole effective mass (${m}_{\rm{h}({\rm{avg}})}^{* }=2{[\frac{1}{{m}_{{\rm{h}}(\Gamma {\rm{X}})}^{* }}+\frac{1}{{m}_{{\rm{h}}(\Gamma {\rm{M}})}^{* }}]}^{-1}$) of 0.369*m*_0_ (Supplementary Table 1). The degenerate, relatively flat and dispersive CBM values correspond to two types of electron mass (for example, 1.67*m*_0_ and 0.34*m*_0_, respectively, along the Γ–M direction). Taking into account masses along different directions, we obtained an averaged electron effective mass (${m}_{\rm{e}({\rm{avg}})}^{* }$) of 0.427*m*_0_ (Supplementary Fig. 46). Although the isomeric **2DCP-MPc** can, in principle, exist (for example, at the edges of the crystal domains), they do not change the band structure of the 2DCP (for example, bandgap, dispersion and effective mass; Supplementary Fig. 47).

We then explored the electronic structures of the slipped-AA-stacked **2DCP-MPc**s in the full K path in space to address the degeneration of conduction bands (Fig. 3c,d). The direct bandgaps of the slipped-AA-stacked **2DCP-MPc**s are reduced with respect to the monolayers due to the interlayer π-interaction. From the projected density of states (PDOS), we observe considerable hybridization of the C*p*, N*p* and O*p* orbitals at CBM, whereas only the contribution of the C*p* orbital to VBM is evident; it is also clear that the metal centres (that is, Ni or Cu) barely hybridize with the polymer backbone composed of C, N and O elements. To understand the role of the metal centre in the electronic structure^15^, we project metal *d*-orbital-resolved band structures for the slipped-AA-stacked **2DCP-MPc**s. Despite the similar PDOS, the Ni*d* orbital contributes more than the Cu*d* orbital to the band edges (Supplementary Fig. 48; Supplementary Fig. 49 shows the calculation for the model compounds), thus resulting in a smaller effective mass for **2DCP-NiPc** than that for **2DCP-CuPc** (0.137*m*_0_ versus 0.172*m*_0_, as shown below).

It is notable that the slipped-AA-stacked **2DCP-MPc**s present significant dispersion in VBM and CBM (Fig. 3c,d). Such strongly dispersive bands have not been observed so far in other 2DCPs^11,15^. Moreover, the effective masses are found to be anisotropic (Supplementary Table 2). For instance, the slipped-AA-stacked **2DCP-NiPc** shows the smallest hole and electron effective masses of 0.125*m*_0_ and 0.139*m*_0_, respectively, along the Γ−Y path (and 0.129*m*_0_ and 0.145*m*_0_ for **2DCP-CuPc**), whereas the Γ−Z direction provides larger hole and electron effective masses of 0.803*m*_0_ and 1.664*m*_0_ (and 2.338*m*_0_ and 2.275*m*_0_ for **2DCP-CuPc**), respectively. We then individually calculated the averaged hole or electron effective mass (${m}_{\mathrm{h}({\rm{avg}})}^{* }{\rm{or}}\,{m}_{\mathrm{e}({\rm{avg}})}^{* }=n{[\frac{1}{{m}_{1}^{* }}+\frac{1}{{m}_{2}^{* }}+\frac{1}{{m}_{3}^{* }}+\ldots \frac{1}{{m}_{n}^{* }}]}^{-1}$), aiming at evaluating the overall transport properties of the charge carriers along the slipped-AA-stacked **2DCP-MPc** backbone, which yields 0.240*m*_0_ and 0.319*m*_0_ for **2DCP-NiPc** (0.310*m*_0_ and 0.386*m*_0_ for **2DCP-CuPc**), respectively. Based on the obtained ${m}_{\mathrm{h}({\rm{avg}})}^{* }$ and ${m}_{\mathrm{e}({\rm{avg}})}^{* }$ values, we further infer the electron–hole reduced effective mass (${m}^{* }$; $\frac{1}{{m}^{* }}=\frac{1}{{m}_{\mathrm{e}({\rm{avg}})}^{* }}+\frac{1}{{m}_{\mathrm{h}({\rm{avg}})}^{* }}$) to be 0.137*m*_0_ and 0.172*m*_0_ for **2DCP-NiPc** and **2DCP-CuPc**, respectively.

### Optical absorption and time-resolved THz spectroscopy

**Fig. 4:**
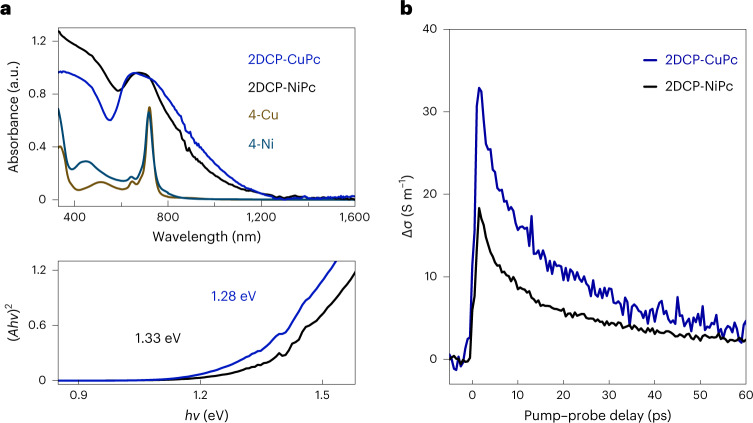
Optical absorption spectra and time-resolved THz spectroscopy of 2DCP-MPcs. **a**, Ultraviolet–visible–near-infrared absorption of **2DCP-MPc** films on fused silica and the starting monomers dissolved in dimethyl sulfoxide (top) and the Tauc plots of the **2DCP-MPc**s (bottom). **b**, Time-resolved THz photoconductivity of **2DCP-CuPc** and **2DCP-NiPc** films. The samples were photoexcited by above-bandgap excitation using 1.55 eV ultrashort laser pulses. Here ∆*σ* is the photoinduced conductivity. Source data

**Fig. 5:**
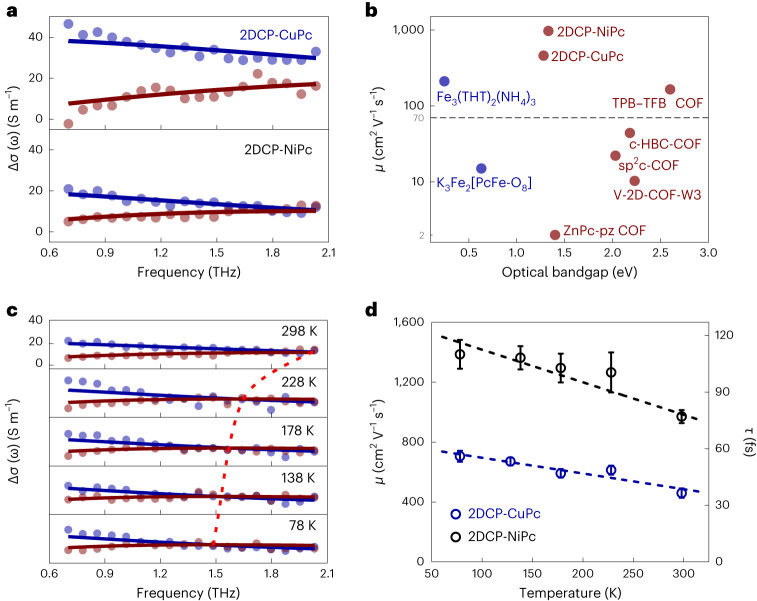
Frequency-resolved THz photoconductivity of 2DCP-MPcs. **a**, Frequency-resolved complex THz photoconductivity measured at ~0.5 ps after the maximum photoconductivity of **2DCP-CuPc** (top) and **2DCP-NiPc** (bottom). The solid lines correspond to the Drude fits describing the real and imaginary parts of the complex THz photoconductivity. **b**, Comparison of charge carrier mobilities and optical bandgaps of **2DCP-MPc**s with reported COFs (light wine spots) and MOFs (light blue spots) measured by THz spectroscopy at ambient temperature (Supplementary Fig. 61 and Supplementary Table 4 provide a comprehensive comparison and references). The reported powder samples exhibit mobilities below 70 cm^2^ V^−1^ s^−1^ (underneath the dashed reference line). **c**, *T*-dependent frequency-resolved complex THz photoconductivity measured at ~0.5 ps after the maximum photoconductivity of **2DCP-NiPc**. The dashed line highlights the shift in the intersection of the real and imaginary components of the complex THz photoconductivity; the intersection corresponds to the momentum scattering frequency following the Drude model, which is inversely proportional to the mobility. **d**, *T*-dependent THz mobilities (left *y* axis) and scattering times (right *y* axis) of **2DCP-CuPc** and **2DCP-NiPc**. The error bars originate from the uncertainty of the obtained charge scattering time from the Drude fit. The dashed lines serve as guides to the eye. Source data

To experimentally explore their transport properties, we grew **2DCP-NiPc** and **2DCP-CuPc** films (with facile charge migration^9,11,12^ compared with the powder samples) on fused silica substrates in the polycondensation reaction mixtures (~1 μm thickness; Supplementary Figs. 50–57 provide the characterizations). The ultraviolet–visible–near-infrared absorption spectra of the **2DCP-MPc** films show a broad Q-band absorption in the range of 550−1,000 nm (Fig. 4a, top). This result suggests a considerable π-electron delocalization, which contributes to optical bandgaps as low as 1.33 and 1.28 eV for **2DCP-NiPc** and **2DCP-CuPc**, respectively (the Tauc plots are shown in Fig. 4a; Supplementary Fig. 58 provides a discussion). These bandgap values are the smallest among the reported 2D c-COFs (Fig. 5b)^15,45^, confirming that the BBL-ladder-type structure confers a narrow bandgap onto the polymer backbone.

We then performed time-resolved photoconductivity measurements on **2DCP-MPc** films using THz spectroscopy (Methods describes its working principle). Briefly, following the photoinjection of free charge carriers into **2DCP-MPc**s by above-bandgap optical femtosecond laser excitation (1.55 eV photon energy; Supplementary Fig. 59 provides the discussion), the high-frequency THz conductivity is determined using a freely propagating THz pulse. Figure 4b depicts the pump-induced conductivity, that is, photoconductivity (∆*σ*), of **2DCP-NiPc** and **2DCP-CuPc** films as a function of time after optical excitation. Both samples show very similar features in the ∆*σ* dynamics: although their sub-picosecond rise is associated with free carrier generation following optical excitation, the subsequent decay in ~20 ps follows a biexponential function (Supplementary Table 3; Supplementary Fig. 60 shows the fluence-dependent THz photoconductivity dynamics) and can be interpreted as free carrier localization (for example, due to trapping or exciton formation^46^) within the **2DCP-MPc**s.

### Frequency-resolved THz photoconductivity measurements

To elucidate the charge transport mechanism, we further measured the complex frequency-resolved photoconductivity (∆*σ*(*ω*)) on the **2DCP-MPc** films (Fig. 5a). We find that the conduction of photogenerated charge carriers can be well described by the Drude model (Methods)—a characteristic response seen in conventional high-mobility inorganic semiconductors^47^, for example, GaAs and Si. From the best fit to the data, we can infer the averaged charge carrier scattering times (*τ*) and consequently estimate the charge carrier mobilities (*μ*) as follows: $\mu =\frac{e\tau }{{m}^{\ast }}$, where *e* is the elementary charge. Owing to the long *τ* (76 ± 3 and 45 ± 3 fs) and small m* (0.137*m*_0_ and 0.172*m*_0_) of the slipped-AA-stacked **2DCP-NiPc** and **2DCP-CuPc**, respectively, the estimate yields exceptionally high mobilities of 971 ± 44 cm^2^ V^−1^ s^−1^ for **2DCP-NiPc** and 460 ± 31 cm^2^ V^−1^ s^−1^ for **2DCP-CuPc** at room temperature, superior to those of the reported linear conjugated polymers including graphene nanoribbons^41^, COFs (for example, 165 cm^2^ V^−1^ s^−1^ for TPB-TFB COF^11^) and metal–organic frameworks (MOFs; for example, 211 cm^2^ V^−1^ s^−1^ for Fe_3_(THT)_2_(NH_4_)_3_^9^) characterized by THz spectroscopy (Fig. 5b and Supplementary Fig. 61; Supplementary Table 4 describes *μ*, *τ* and m*). The outstanding mobility and relatively long carrier lifetime (compared with COFs^15,36,37^ characterized by the same technique) lead to diffusion lengths of ~237 and ~157 nm for **2DCP-NiPc** and **2DCP-CuPc**, respectively. As part of control studies, we also measured **2DCP-NiPc** and **2DCP-CuPc** in powder forms, similarly displaying very high charge carrier mobility of 219 ± 14 and 183 ± 19 cm^2^ V^−1^ s^−1^, respectively (Supplementary Fig. 62), largely exceeding the state-of-the-art powder-based COFs and MOFs.

## Discussion

The Drude-like photoconductivity dispersion and outstanding charge carrier mobility in **2DCP-MPc**s demonstrate the band-like transport character of the samples. To gain further insight, we conduct temperature (*T*)-dependent photoconductivity on **2DCP-MPc** films grown on fused silica. As shown in Supplementary Figs. 63 and 64, the photoconductivity amplitude goes up slightly by lowering *T*. To disentangle the contributions eventually arising from changes in *μ* and those linked to charge carrier density *N* (Δ*σ* = *e* × *µ* × *N*), we performed *T*-dependent frequency-resolved complex conductivity ∆*σ*(*ω*) measurements (Fig. 5c). We find that all the photoconductivity spectra can be described well by the Drude model. The *T*-dependent *τ* is summarized in Fig. 5d. The inferred *τ* increases substantially for both samples (for example, from 76 ± 3 to 108 ± 7 fs for **2DCP-NiPc** by lowering *T* from 298 to 78 K). These results indicate that electron–phonon scattering is dominant in determining the charge carrier mobility in **2DCP-MPc**s, entirely consistent with the band-like transport picture. At 78 K, phonon scattering is minimized, and the obtained *τ* implies mobilities as high as 1,386 ± 96 and 706 ± 37 cm^2^ V^−1^ s^−1^ in **2DCP-NiPc** and **2DCP-CuPc**, respectively. We observed a higher carrier mobility and its stronger dependence of *τ* on *T* in **2DCP-NiPc** compared with **2DCP-CuPc**. This observation can, in part, be attributed to the lower charge carrier effective mass in **2DCP-NiPc**, and further rationalized by a scenario in which **2DCP-NiPc** suffers much less from defect scattering (due to, for example, better film quality and thus reduced defect density; Supplementary Figs. 52 and 53 show the film quality characterizations). Electron–phonon scattering-limited transport proves our design principle of high-mobility 2DCPs, that is, the BBL-ladder-type structure confers significant in-plane conjugation to the **2DCP-MPc**s, leading to predominant intralayer charge transfer; further, the ordered crystalline polymer networks ensure relatively low defect and impurity densities. Moreover, the inferred plasma frequency squared (${w}_{\rm{p}}^{2}$) and thus *N* (*N* ≈ $\frac{{w}_{\rm{p}}^{2}}{{m}^{* }}$) are decreased by ~18% and ~34% for **2DCP-NiPc** and **2DCP-CuPc**, respectively, by lowering *T* from 298 to 78 K (Supplementary Fig. 65). This may be attributed, for example, to the more favorable internal conversion of free carriers to excitons at low temperatures, which reduces the free carrier population^8^.

In this work, we have demonstrated highly delocalized π-electrons and dispersive electronic bands in two phthalocyanine-based BBL-ladder-type crystalline, semiconducting 2DCPs. They present narrow optical bandgaps of 1.3 eV and a band transport feature with high charge mobility on the order of hundreds of cm^2^ V^−1^ s^−1^ at room temperature, showing the potential of 2DCPs in high-performance organic (opto)electronics. Further efforts to develop highly crystalline or single-crystalline **2DCP-MPc**s and delaminate them into thin layers will not only enable a fundamental understanding of the structure–property relationships (by exploring the role of intrinsic and extrinsic contributions and minimizing the extrinsic influence) but also render the integration of high-mobility semiconducting 2DCPs into organic (opto)electronic and nanoelectronic devices.

## Methods

### Synthesis of 2DCP-MPcs

A glass ampule was charged with **4-M** (ref. ^15^) (8.0 mg, 8.1 μmol), **1** (4.3 mg, 16.0 μmol), PTSA (6.2 mg, 32.6 μmol) and DMI/*o*-dichlorobenzene (0.4/0.2 ml). The ampule was sonicated at room temperature for 10 min, degassed by three freeze–pump–thaw cycles, sealed under a vacuum and heated at 200 °C for 5 days. After cooling to room temperature, the precipitate was filtered and sequentially washed with dimethylformamide, dimethyl sulfoxide, H_2_O, ethanol and acetone. After Soxhlet extraction with tetrahydrofuran (4 h), ethanol (8 h) and diethyl ether (2 h), the sample was collected and dried under a vacuum at 150 °C overnight to give **2DCP-MPc**s as dark green powders in ~90% yield.

To prepare **2DCP-MPc** as a thin-film sample, a fused silica substrate cleaned by piranha solution (at 120 °C for 5 h) was placed vertically in the above reaction mixture in DMI/*o*-dichlorobenzene (0.50/0.25 ml). The obtained film on the substrate was washed by immersing in dimethylformamide and then in acetone. Each solvent was changed three times. After natural drying, **2DCP-CuPc** and **2DCP-NiPc** were obtained as dark green and greyish green films on the fused silica substrates, respectively.

### Electronic band structures and effective mass estimates

DFT calculations were carried out using the Vienna ab initio simulation package version 5.4.1 (Supplementary Information provides details and references). We used the DFT + *U* approach to describe the localized *d* orbitals of Cu and Ni ions. The effective Coulomb (*U*) and exchange (*J*) terms were set to 4 and 1 eV, respectively. A Monkhorst–Pack Γ-centred grid with dimensions of 2 × 2 × 1 was used for the *k*-point sampling of the Brillouin zone for the monolayer during geometry optimization and dimensions of 4 × 4 × 1 during band structure calculations. In the computational protocol for the three-dimensional stacking of the studied 2DCPs, the *k*-point grid dimensions were changed to 2 × 2 × 5 for geometry optimization and to 4 × 4 × 10 for the band structure calculations, and Grimme’s D2 correction for the interlayer dispersion interactions was applied. All the models were subjected to full geometry optimization (cell parameters and ionic positions). The corresponding electronic band structures were evaluated along the Γ–X–M–Γ and Γ–X|Y–Γ–Z|*R*_2_–Γ–*T*_2_|*U*_2_–Γ–*V*_2_ path in the Brillouin zone for the monolayer and multilayered structures, respectively. The effective masses for the electrons and holes were calculated by a parabolic fit of the VBM and CBM using the sumo Python toolkit^48^ or manually in the case of nearly degenerate electronic states.

### Time- and frequency-resolved THz photoconductivity measurements

THz photoconductivity^49^ measurements were performed by an optical pump THz probe spectrometer. The setup was powered by a titanium:sapphire laser amplifier system, which generated ultrafast laser pulses with a central wavelength of 800 nm. The pulses have a duration of ~50 fs and a repetition rate of 1 kHz, which are further used for optical excitation, THz generation and detection. Single-cycle THz pulses of ~1 ps duration were generated via optical rectification by pumping a 1-mm-thick (110) ZnTe crystal with 800 nm laser pulses. The time-dependent electric field of the generated THz pulse was mapped out by free-space electro-optic sampling. The measurements were performed in the transmission mode in a dry N_2_ environment, and the samples were photoexcited by the 800 nm laser pulses with pump fluences in the range of ~0.4−6.2 mJ cm^–2^. The temperature-dependent photoconductivity measurements of **2DCP-MPc** film samples were conducted in the presence of a cryostat under vacuum conditions (pressure < 2 × 10^−4^ mbar). By measuring the transmitted THz traces with and without photoexcitation in the time domain (*E*_pump_(t) and *E*_0_(t)) and further converting them into the frequency domain by Fourier transform (*E*_pump_(*ω*) and *E*_0_(*ω*)), we can obtain their complex photoconductivity (∆*σ*(*ω*)) as per the following thin-film approximation:

$$\Delta{\sigma} \left(\omega \right)=-\frac{{n}_{1}+{n}_{2}}{{Z}_{0}l}\times \frac{({E}_{{\rm{pump}}}\left(\omega \right)-{E}_{0}\left(\omega \right))}{{E}_{0}(\omega )},$$

where *Z*_0_ = 377 Ω is the impedance of free space; *n*_1_ and *n*_2_ are the refractive indices of the media before and after the sample, respectively; and *l* is the excitation thickness.

Here ∆*σ*(*ω*) of **2DCP-MPc** films was described by the Drude model, which accounts for the transport of delocalized free carriers in the material:

$$\sigma \left({{\omega }}\right)=\frac{{{{\omega }}}_{\rm{p}}^{2}{\varepsilon }_{0}\tau }{1-{\rm{i}}{{\omega }}\tau },$$

where *ω*_p_, *ε*_0_ and *τ* are the plasma frequency, vacuum permittivity and Drude scattering time, respectively.

The diffusion length *L* can be deduced from the charge mobility *μ* and carrier lifetime *t* as follows:

$$L=\sqrt{D{{\times }}t},\,{\rm{where}}\,{D}=\frac{\mu {k}_{\rm{B}}T}{e}$$

and *k*_B_ and *T* denote the Boltzmann constant and temperature, respectively.

## Online content

Any methods, additional references, Nature Portfolio reporting summaries, source data, extended data, supplementary information, acknowledgements, peer review information; details of author contributions and competing interests; and statements of data and code availability are available at 10.1038/s41563-023-01581-6.

## Supplementary information

Supplementary Information Supplementary Schemes 1–3, Figs. 1–65, Tables 1–4, References 1–58, Methods, Materials and Synthesis procedures.

Supplementary Data Coordinate files for slipped-AA-stacked 2DCP-CuPc, slipped-AA-stacked 2DCP-NiPc, monolayer 2DCP-CuPc and monolayer 2DCP-NiPc.

## Data Availability

All data shown in the main text are available via figshare at 10.6084/m9.figshare.22700377. All other datasets generated during and/or analysed during this study are available from the corresponding authors on reasonable request. The theoretical calculations presented in the paper were carried out using publicly available codes. Source data are provided with this paper.

## Source data

Source Data Fig. 1 HOMO and LUMO energies and HOMO–LUMO gap data (Fig. 1b).
Source Data Fig. 2 Powder X-ray diffraction data (Fig. 2c).
Source Data Fig. 3 Electronic band structure data (Fig. 3a–d).
Source Data Fig. 4 Ultraviolet–visible–near-infrared absorption data (Fig. 4a) and THz photoconductivity data (Fig. 4b).
Source Data Fig. 5 THz photoconductivity data (Fig. 5a), charge carrier mobilities and optical bandgaps (Fig. 5b), THz photoconductivity data (Fig. 5c) and THz mobilities and charge scattering times (Fig. 5d).

## References

[1] Shirakawa, H., Louis, E. J., MacDiarmid, A. G., Chiang, C. K. & Heeger, A. J. Synthesis of electrically conducting organic polymers: halogen derivatives of polyacetylene, (CH)_*x*_. *J. Chem. Soc., Chem. Commun.* 578–580 (1977).

[2] Fratini S, Nikolka M, Salleo A, Schweicher G, Sirringhaus H (2020). Charge transport in high-mobility conjugated polymers and molecular semiconductors. Nat. Mater..

[3] Oh JY (2016). Intrinsically stretchable and healable semiconducting polymer for organic transistors. Nature.

[4] Wang C, Dong H, Hu W, Liu Y, Zhu D (2012). Semiconducting π-conjugated systems in field-effect transistors: a material odyssey of organic electronics. Chem. Rev..

[5] Müllen K (2016). Molecular defects in organic materials. Nat. Rev. Mater..

[6] Gutzler R, Perepichka DF (2013). π-Electron conjugation in two dimensions. J. Am. Chem. Soc..

[7] Haldar R (2021). Interplay of structural dynamics and electronic effects in an engineered assembly of pentacene in a metal–organic framework. Chem. Sci..

[8] Ghosh S (2022). Band-like transport of charge carriers in oriented two-dimensional conjugated covalent organic frameworks. Chem. Mater..

[9] Dong R (2018). High-mobility band-like charge transport in a semiconducting two-dimensional metal–organic framework. Nat. Mater..

[10] Xie LS, Skorupskii G, Dincă M (2020). Electrically conductive metal–organic frameworks. Chem. Rev..

[11] Fu S (2022). Outstanding charge mobility by band transport in two-dimensional semiconducting covalent organic frameworks. J. Am. Chem. Soc..

[12] Wang M, Dong R, Feng X (2021). Two-dimensional conjugated metal–organic frameworks (2D c-MOFs): chemistry and function for MOFtronics. Chem. Soc. Rev..

[13] Jing Y, Heine T (2019). Two-dimensional kagome lattices made of hetero triangulenes are Dirac semimetals or single-band semiconductors. J. Am. Chem. Soc..

[14] Galeotti G (2020). Synthesis of mesoscale ordered two-dimensional π-conjugated polymers with semiconducting properties. Nat. Mater..

[15] Wang M (2019). Unveiling electronic properties in metal–phthalocyanine-based pyrazine-linked conjugated two-dimensional covalent organic frameworks. J. Am. Chem. Soc..

[16] Jin Y (2020). Confined growth of ordered organic frameworks at an interface. Chem. Soc. Rev..

[17] Wang Y (2022). Facile construction of fully *sp*^2^-carbon conjugated two-dimensional covalent organic frameworks containing benzobisthiazole units. Nat. Commun..

[18] Li X (2022). Constructing ambivalent imidazopyridinium-linked covalent organic frameworks. Nat. Synth..

[19] Evans AM (2021). Thermally conductive ultra-low-*k* dielectric layers based on two-dimensional covalent organic frameworks. Nat. Mater..

[20] Karak S (2017). Constructing ultraporous covalent organic frameworks in seconds via an organic terracotta process. J. Am. Chem. Soc..

[21] Haase F (2018). Topochemical conversion of an imine- into a thiazole-linked covalent organic framework enabling real structure analysis. Nat. Commun..

[22] Ascherl L (2018). Solvatochromic covalent organic frameworks. Nat. Commun..

[23] Kang C (2022). Growing single crystals of two-dimensional covalent organic frameworks enabled by intermediate tracing study. Nat. Commun..

[24] Evans AM (2022). Two-dimensional polymers and polymerizations. Chem. Rev..

[25] Ma T (2018). Single-crystal X-ray diffraction structures of covalent organic frameworks. Science.

[26] Zhao S (2021). Hydrophilicity gradient in covalent organic frameworks for membrane distillation. Nat. Mater..

[27] Li L (2022). Isoreticular series of two-dimensional covalent organic frameworks with the kgd topology and controllable micropores. J. Am. Chem. Soc..

[28] Cusin L, Peng H, Ciesielski A, Samorì P (2021). Chemical conversion and locking of the imine linkage: enhancing the functionality of covalent organic frameworks. Angew. Chem. Int. Ed..

[29] Zhuang X (2016). A two-dimensional conjugated polymer framework with fully *sp*^2^-bonded carbon skeleton. Polym. Chem..

[30] Jadhav T (2019). 2D poly(arylene vinylene) covalent organic frameworks via aldol condensation of trimethyltriazine. Angew. Chem. Int. Ed..

[31] Jin E (2017). Two-dimensional *sp*^2^ carbon–conjugated covalent organic frameworks. Science.

[32] Lyu H, Diercks CS, Zhu C, Yaghi OM (2019). Porous crystalline olefin-linked covalent organic frameworks. J. Am. Chem. Soc..

[33] Acharjya A, Pachfule P, Roeser J, Schmitt F-J, Thomas A (2019). Vinylene-linked covalent organic frameworks by base-catalyzed aldol condensation. Angew. Chem. Int. Ed..

[34] Bi S (2019). Two-dimensional semiconducting covalent organic frameworks via condensation at arylmethyl carbon atoms. Nat. Commun..

[35] Yue Y (2021). Stable bimetallic polyphthalocyanine covalent organic frameworks as superior electrocatalysts. J. Am. Chem. Soc..

[36] Xing G (2022). Nonplanar rhombus and kagome 2D covalent organic frameworks from distorted aromatics for electrical conduction. J. Am. Chem. Soc..

[37] Jin E (2022). Module-patterned polymerization towards crystalline 2D *sp*^2^-carbon covalent organic framework semiconductors. Angew. Chem. Int. Ed..

[38] Wang M (2020). High-mobility semiconducting two-dimensional conjugated covalent organic frameworks with p-type doping. J. Am. Chem. Soc..

[39] Schlüter A-D (1991). Ladder polymers: the new generation. Adv. Mater..

[40] Scherf U (1999). Ladder-type materials. J. Mater. Chem..

[41] Wang X (2022). Cove-edged graphene nanoribbons with incorporation of periodic zigzag-edge segments. J. Am. Chem. Soc..

[42] Che S, Fang L (2020). Porous ladder polymer networks. Chem.

[43] Noh H-J (2022). Hydrophenazine-linked two-dimensional ladder-type crystalline fused aromatic network with high charge transport. Chem.

[44] Noh H-J (2020). Vertical two-dimensional layered fused aromatic ladder structure. Nat. Commun..

[45] Keller N, Bein T (2021). Optoelectronic processes in covalent organic frameworks. Chem. Soc. Rev..

[46] Tries A (2020). Experimental observation of strong exciton effects in graphene nanoribbons. Nano Lett..

[47] Hendry E, Koeberg M, Pijpers J, Bonn M (2007). Reduction of carrier mobility in semiconductors caused by charge-charge interactions. Phys. Rev. B.

[48] Ganose AM, Jackson JA, Scanlon DO (2018). sumo: command-line tools for plotting and analysis of periodic ab initio calculations. J. Open Source Softw..

[49] Ulbricht R, Hendry E, Shan J, Heinz TF, Bonn M (2011). Carrier dynamics in semiconductors studied with time-resolved terahertz spectroscopy. Rev. Mod. Phys..

