# Supplementary material for: Exceptionally high charge mobility in phthalocyanine-based poly(benzimidazobenzophenanthroline)-ladder-type two-dimensional conjugated polymers
Source: Nat Mater. 2023 Jun 19;22(7):880–7. doi: 10.1038/s41563-023-01581-6 (PMC10313522; doi:10.1038/s41563-023-01581-6)
Supplement: Supplementary file 1 — Supplementary Schemes 1–3, Figs. 1–65, Tables 1–4, References 1–58, Methods, Materials and Synthesis procedures. [file 41563_2023_1581_MOESM1_ESM.pdf]

Table of contents

Section A. Supplementary Methods………………………………………..…. S2

Section B. Materials and Synthetic Procedures………………………… S5

Section C. Supplementary Figures………………………………………. S9

Section D. Supplementary Tables…………………………………..…….. S59

Section E. Supplementary References……………………………..….. S66

Section A. Supplementary Methods

Instrumentation and characterization

Nuclear magnetic resonance (NMR) spectroscopy was carried out on Bruker AV-II 300 spectrometer operating at 300.1 MHz for ^{1}H NMR. Chemical shifts are given in ppm relative to tetramethylsilane (TMS). **Solid-state NMR** spectra were recorded on BRUKER Ascend 300 MHz spectrometer using a commercial 2.5 mm MAS NMR probe and operating at a resonance frequency of 75.48 MHz for ^{13}C and 300.13 MHz for ^{1}H. The MAS frequency was 15 kHz. Cross polarization (CP) and SPINAL ^{1}H decoupling were used during signal acquisition. The ^{1}H and ^{13}C chemical shift were referenced relative to TMS using adamantane as secondary standard. **Fourier transform infrared (FTIR)** spectroscopy was performed on a Bruker Optics ALPHA-E spectrometer. **High resolution matrix-assisted laser desorption ionization time of flight mass spectrometry (MALDI-TOF MS)** was recorded on a Bruker Autoflex Speed MALDI TOF MS (Bruker Daltonics, Bremen, Germany). The instrument is equipped with an Agilent Series 1200 HPLC binary pump, and Autosampler, using Mass Hunter software. **UV-visible-near IR absorption** was measured on a SolidSpec-3700i/3700i DUV UV-VIS-NIR Spectrophotometer at room temperature. **Powder X-ray diffraction (PXRD)** were obtained on an X-ray diffractometer (Dectris Mythen 1K Strip Detector, Stoe Stadi-P) using Cu-Kα radiation (λ = 0.15418 nm) at 40 kV and 40 mA at room temperature in transmission geometry. **Scanning electron microscopy (SEM)** and **energy-dispersive X-ray (EDX) spectroscopy** were carried out with a Gemini 500 (Carl Zeiss) system equipped with an Oxford Xmax^{N}-150 EDX detector. **Thermal gravimetric analysis (TGA)** was characterized using a Netzsch STA 449C instrument under argon atmosphere with a heating rate of 5ºC/min in ceramic crucible. **Nitrogen physisorption** was conducted on a Micromeritics, TriStar II Plus automated, three-station, surface area and porosity analyzer at 77 K for N_2. **X-ray photoelectron spectroscopy (XPS)** was acquired on an AXIS Ultra DLD (Kratos) system using Al Kα radiation.

Reaction energetics and HOMO-LUMO energy levels

Theoretical calculations of model compounds were performed using the Gaussian 16 program.[1] The B3LYP functional was used for geometry optimization in the ground state. The 6-31G(d) basis set was used. The dispersion correction was conducted by Grimme's D3 version with the BJ damping function.[2] All geometry optimization was done in the gas phase.

For transition state (TS) calculation, all the structures were optimized in gas phase by using B3LYP level of density functional theory with the 6-31G* basis. To confirm the accuracy of the TS, frequency calculation was performed. The Gibbs free energies of reaction ($\Delta_r G^o$) at room temperature (298 K) can be calculated using following equations[3]:

$$H_{corr} = E_{tot} + k_B T$$

$$G_{corr} = H_{corr} - TS_{tot}$$

$$\Delta_r G^o(T) = \sum(\varepsilon_0 + G_{corr})_{products} - \sum(\varepsilon_0 + G_{corr})_{reactants}$$

where H_{corr} is the thermal correction to Enthalpy; E_{tot} is the correction to the internal thermal energy; k_B is the Boltzmann constant; T is the temperature; G_{corr} is the thermal correction to Gibbs free energy (thermal Free Energies); S_{tot} is the correction to the internal Entropy; ε_0 is the total electronic energy and nuclear repulsion energy. In our case, the isomerization process represents the reaction.

Structural modeling and Pawley refinement of 2DCPs

Density functional theory (DFT) calculations were carried out using the Vienna ab-initio Simulation Package (VASP)[4,5] version 5.4.1. The electronic wave-functions were expanded in a plane-wave basis set with a kinetic energy cutoff of 500 eV. The geometry optimization convergence was set to forces acting on the ions were smaller than 0.015 eV A^{-1}. Electron-ion interactions were described using the projector augmented wave (PAW) method[6,7]. Generalized gradient approximation (GGA)[8] of the exchange-correlation energy in the form of Perdew-Burke-Ernzerhof (PBE) was applied[9]. We used DFT+U approach to describe the localized d-orbitals of Cu and Ni ions. The effective Coulomb (U) and exchange (J) terms were set to 4 and 1 eV, respectively[10], such approach was already

successfully applied for similar systems[11]. Monkhorst-Pack Gamma-centered grid[12] with 2×2×1 dimension was used for K-point sampling of the Brillouin zone for the monolayer during the geometry optimization and 4×4×1 for band structure calculations. In the computational protocol for the three-dimensional (3D) stacking of the studied 2DCPs, the K-point grid dimension was changed to 2×2×5 for the geometry optimization and 4×4×10 for the band structure calculations, and Grimme-D2 correction for the interlayer dispersion interactions was applied[13]. In order to determine the full high-symmetry K-points in the Brillouin zone a VASPKIT[14] code for pre- and post-processing of the VASP calculated data was used. The PBE-D2 method can lead to a relative error up to 3% in lattice parameter for metal-organic frameworks[15]. Thus, an error of 3% was considered during calculating the PXRD patterns of the final structures. The 2DCP monolayer was modeled by adding a large vacuum space, 10 Å, in the direction normal to the monolayer. The unit cell used in the calculations of the 3D models contains two layers. All the models were subject of full geometry optimization (cell parameters and ionic positions). The corresponding electronic band structures were evaluated along the Γ-X-M-Γ and Γ-X|Y-Γ-Z|R_2-Γ-T_2|U_2-Γ-V_2 path in the Brillouin zone for the monolayer and multilayered structures, respectively. The effective masses for the electrons and holes were calculated by parabolic fit of the VBM and CBM using SUMO Python toolkit[16] or manually in the case of nearly degenerated electronic states (see details in Supplementary Tables 1 and 2).

For Pawley refinement, the unit cells of the models were refined in the 2θ range ca. 2.5−40° with the experimentally obtained PXRD pattern in the Reflex module of the BIOVA Materials Studio 2020 with fixed atom coordinates.

Section B. Materials and Synthetic Procedures

All the solvents and reagents were purchased from commercial suppliers and used without purification. Octaaminophthalocyaninato metal(II) (**4-M**) was synthesized according to a literature procedure.[17] Naphthalenetetracarboxylic dianhydride (**1**) and 1,3-dimethyl-2-imidazolidinone (DMI) was purchased from TCI Deutschland GmbH. *o*-Dichlorobenzene was supplied by Fisher Scientific GmbH. Copper phthalocyanine (CuPc) and poly(benzimidazobenzophenanthroline) (BBL) 1DCP for Raman measurements were provided by Sigma-Aldrich Chemie GmbH. Fused silica substrates were purchased from Microchemicals GmbH.

Synthesis of 2DCPs and related model compounds

Model compound 3: A mixture of naphthalenetetracarboxylic dianhydride (**1**, 120 mg, 449 μmol), 1,2-phenylenediamine (**2**, 100 mg, 925 μmol) and PTSA (256 mg, 1350 μmol) or trifluoroacetic acid (TFA, 110 μL, 1440 μmol) was stirred in DMI (2 mL) under N_2 at 120 °C for 22 h. After cooling down to room temperature, the precipitate was collected by filtration, washed with dimethylformamide (DMF), H_2O, ethanol, acetone as well as DCM, and dried under vacuum at 120 °C overnight, to give perinone as red solid in 95% yield. The same reaction using PTSA as catalyst performed at 50 °C for ~4 d results in 35% yield. HR MALDI-TOF MS for $C_{26}H_{12}N_4O_2$ (calcd. 412.0960): m/z = 413.1053 ($[M + H]^+$).

Model compound 6: A glass ampule was charged with octaaminophthalocyaninato zinc(II) (**4-Zn**·8HCl) (19.0 mg, 19.2 μmol), 1,8-naphthalic anhydride (**5**, 16.7 mg, 84.5 μmol), PTSA (14.6 mg, 76.8 μmol), and DMI/*o*-dichlorobenzene (0.8/0.4 mL). The ampule was sonicated at room temperature for 5 min, degassed by three freeze-pump-thaw cycles, sealed under vacuum, and heated at 200 °C for 3 days. After cooling to room temperature, the precipitate was filtered, washed with DMF, ethanol and acetone, and dried under vacuum at 120 °C for overnight to give **6** as dark green powders.

2DCP-MPc: A glass ampule was charged with **4-M**·8HCl (M = Ni or Cu, 8.0 mg, 8.1 μmol), **1** (4.3 mg, 16.0 μmol), PTSA (6.2 mg, 32.6 μmol), and DMI/*o*-dichlorobenzene (0.4/0.2 mL).

The ampule was sonicated at room temperature for 10 min, degassed by three freeze–pump–thaw cycles, sealed under a vacuum, and heated at 200 °C for 5 days. After cooling to room temperature, the precipitate was filtered and sequentially washed with DMF, dimethyl sulfoxide (DMSO), H_2O, ethanol and acetone. After Soxhlet extraction with tetrahydrofuran (4 h), ethanol (8 h) and diethyl ether (2 h), the sample was collected and dried under a vacuum at 150 °C overnight to give **2DCP-MPc** as dark green powders in ~90% yield. To prepare **2DCP-MPc** as a thin-film sample, a fused silica substrate cleaned by piranha solution (at 120 °C for 5 h) was placed vertically in the above reaction mixture in DMI/*o*-dichlorobenzene (0.50/0.25 mL). The obtained film on the substrate was washed by immersing in DMF and then in acetone. Each solvent was changed three times. After natural drying, **2DCP-CuPc** and **2DCP-NiPc** were obtained as dark green and greyish green films on fused silica substrates, respectively.

In-situ exchange experiments of model compounds and competitive reactions

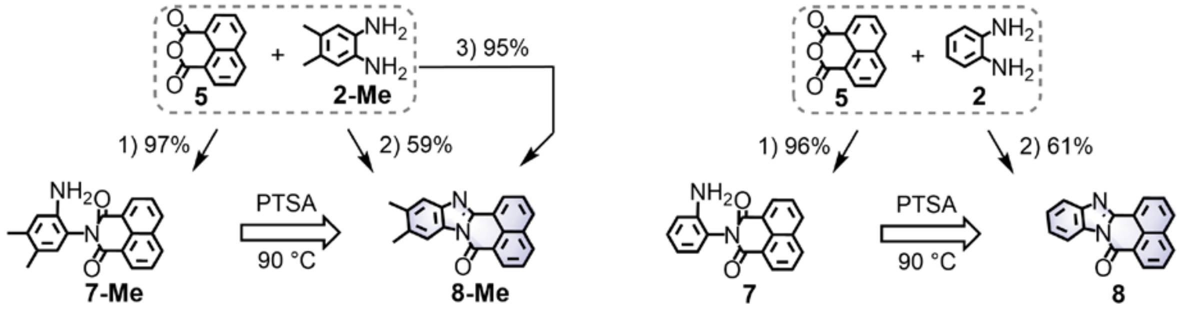

Supplementary Scheme 1. Synthesis of compounds 7, 7-Me, 8, and 8-Me for the exchange experiments. Reaction conditions: 1) catalyst-free, 90 °C, 6 h; 2) catalyst-free, 200 °C, 36 h; 3) PTSA, 90 °C, 6 h.

The condensation between **5** and **2** involves imide-bond formation towards 2-(2-aminophenyl)-1*H*-benzo[*de*]isoquinoline-1,3(2*H*)-dione (**7**) and intramolecular dehydration of **7** to 7*H*-benzo[*de*]benzo[4,5]imidazo[2,1-*a*]isoquinolin-7-one (**8**). Imide-bond formation under basic or catalyst-free condition has been widely employed for synthesizing

crystalline 2D polyimides.[18] However, the intramolecular dehydration from **7** to **8** is rather challenging under these conditions.[19] To enhance the reactivity, we first optimized the synthesis of the *o*-methyl-substituted **8** (**8-Me**). It was synthesized from **5** and *o*-methyl-substituted **2** (**2-Me**) without catalyst in 1,3-dimethyl-2-imidazolidinone (DMI) as solvent at 200 °C for 36 h in 59 % yield (condition 2, pyridine as catalyst is ineffective), which raised significantly up to 95 % when an organic acid, PTSA, was used as catalyst even at 90 °C for 6 h (condition 3).

7-Me: The synthesis was conducted according to a modified literature procedure.[20] A mixture of **5** (100 mg, 505 μmol) and **2-Me** (70 mg, 505 μmol) was stirred in DMF (1 mL) at 90 °C for 6 h. After cooling down to room temperature, H_2O was added. The precipitate was collected by filtration, washed with methanol/H_2O (1:3, v/v), and dried under vacuum at 60 °C overnight, to give **7-Me** as yellow solid in 97% yield. **7** was prepared starting from **5** and **2**.

8-Me: A mixture of **5** (100 mg, 505 μmol) and **2-Me** (70 mg, 505 μmol) was stirred in DMI (1 mL) at 200 °C for 36 h. After cooling down to room temperature, H_2O was added. The precipitate was collected by filtration, washed with H_2O and methanol, and dried under vacuum at 60 °C overnight, to give **8-Me** as dark yellow solid in 59% yield. **8** was prepared starting from **5** and **2**. Using PTSA as catalyst, **8-Me** was synthesized at 90 °C for 6 h.

***In-situ* exchange experiments involving 7-Me, 2 equiv 2, and 3 equiv catalyst: 7-Me** (12 mg, 38 μmol) and **2** (8.2 mg, 76 μmol) were dissolved in DMSO-d_6/D_2O (1800/3.6 μL), and divided into three equal portions in three NMR tubes. PTSA (7.3 mg, 38 μmol), pyridine (3.1 μL, 38 μmol), or no catalyst was added to the NMR tube for corresponding exchange experiment. For the exchange experiments with 9 equiv catalyst, **7-Me** (4 mg, 12.6 μmol), **2** (2.7 mg, 25.0 μmol), and PTSA (21.7 mg, 114 μmol) or TFA (8.7 μL, 114 μmol) were dissolved in DMSO-d_6 (0.6 mL) in the NMR tube.

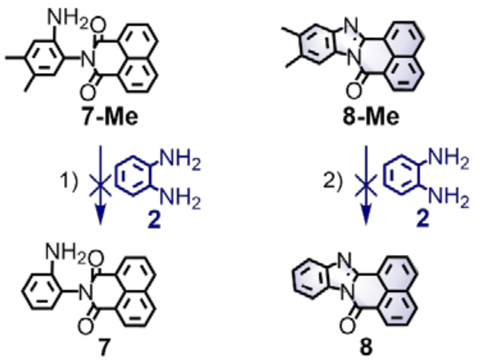

Supplementary Scheme 2. Schematic exchange experiments. Reaction conditions: 1) DMSO/D_2O or DMI/H_2O, catalyst-free or pyridine or PTSA, 90 °C, 12 h; 2) DMI/H_2O, catalyst-free or pyridine or PTSA, 200 °C, 2 d.

Competitive reaction between 5, 2 and 2-Me: A mixture of **5** (100 mg, 505 μmol), **2** (55 mg, 505 μmol), **2-Me** (70 mg, 505 μmol), and 3 equiv catalyst (TFA, PTSA, pyridine, or no catalyst) was stirred in DMF (1mL) at 90°C for 6 h. After cooling down to room temperature, H_2O was added. The precipitate was collected by filtration, washed with methanol/H_2O (1:3, v/v), and dried under vacuum at 60°C overnight.

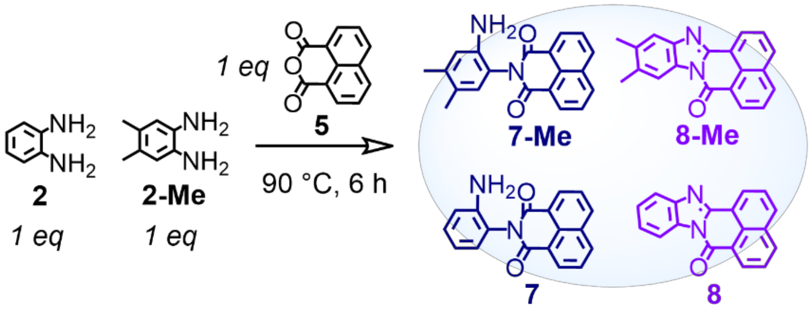

7 : 7-Me : 8 : 8-Me mol%
24 : 76 : 0 : 0 *(no cat.)*
28 : 72 : 0 : 0 *(pyridine)*
0 : 0 : 51 : 49 *(PTSA)*
0 : 0 : 47 : 53 *(TFA)*

Supplementary Scheme 3. Competitive reactions between 5, 2 and 2-Me.

Competitive reactions between the anhydride **5** (1 equiv) and two *o*-diamines of **2** (1 equiv) and 4,5-dimethyl-1,2-phenylenediamine (**2-Me**, 1 equiv) are examined at 90 °C for 6 h. Compared to the basic or catalyst-free condition, an acidic catalyst (PTSA or TFA) results in a widely varied molar ratio of **7-Me**/**7** (equal to the observed products of **8-Me**/**8**) in the product from 3/1 to 1/1 (see details in Supplementary Figures 16–18).

Section C. Supplementary Figures

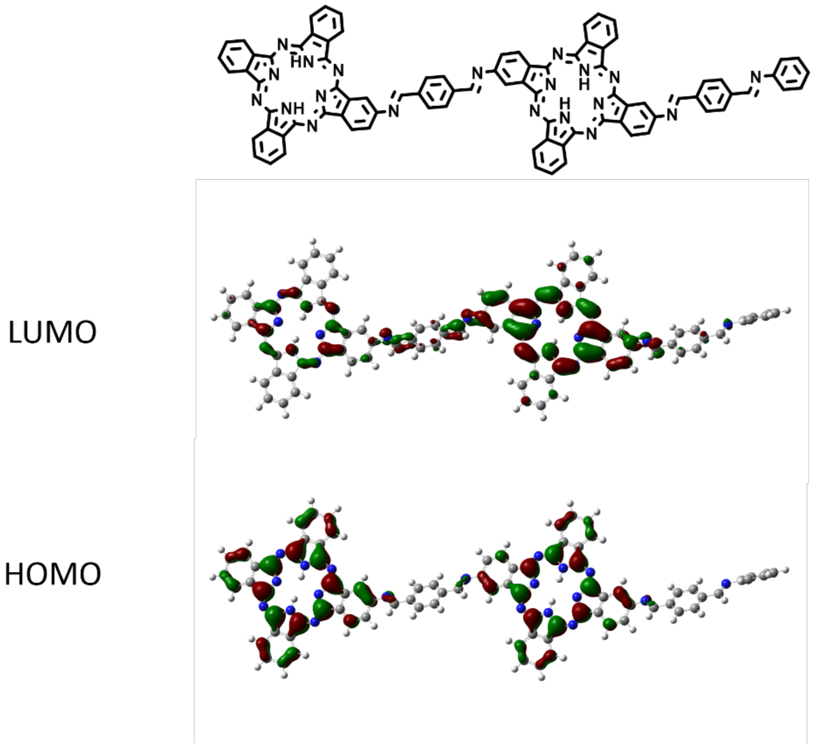

Supplementary Figure 1. Molecular electronic structure of the frontier orbitals of M1.

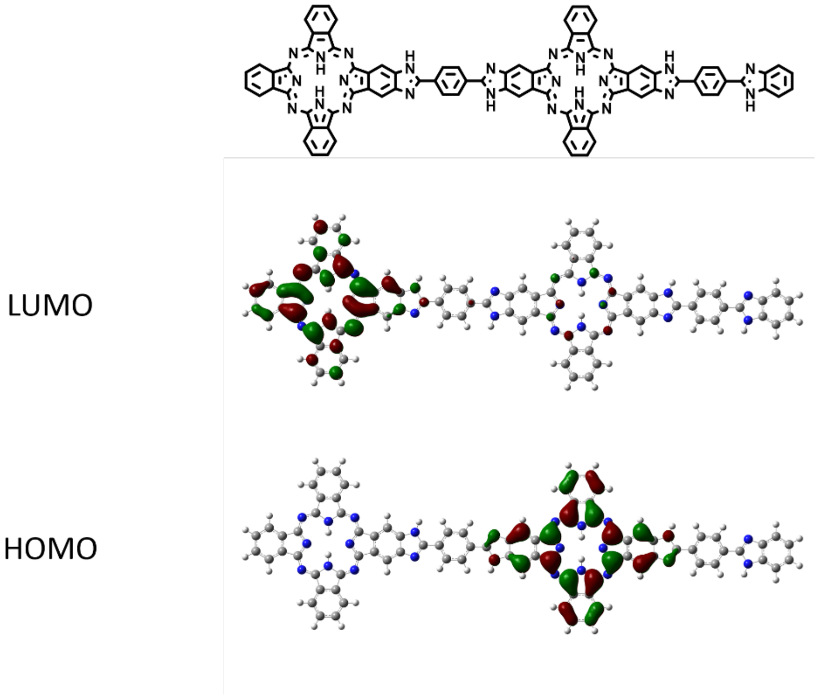

Supplementary Figure 2. Molecular electronic structure of the frontier orbitals of M2.

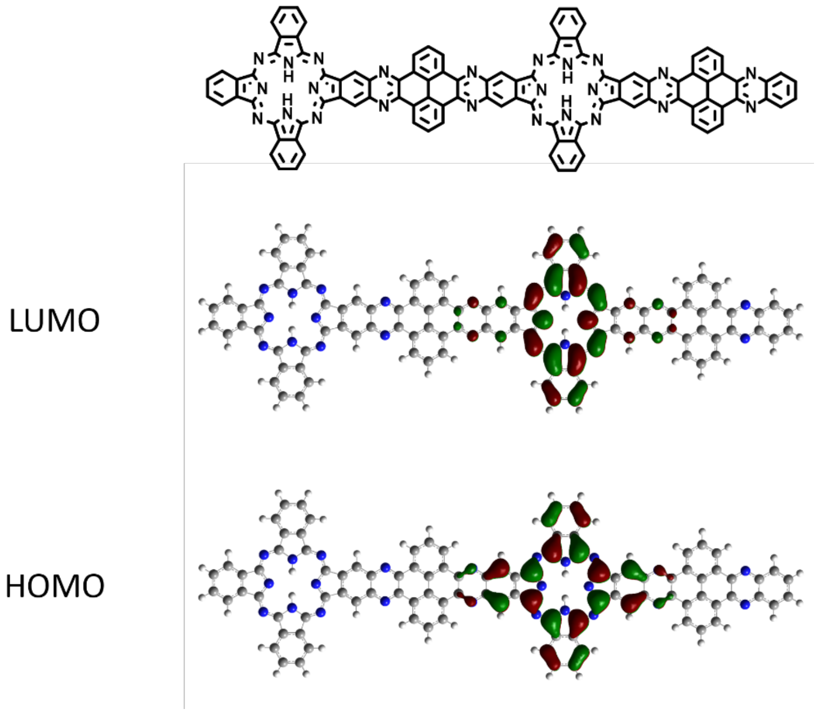

Supplementary Figure 3. Molecular electronic structure of the frontier orbitals of M3.

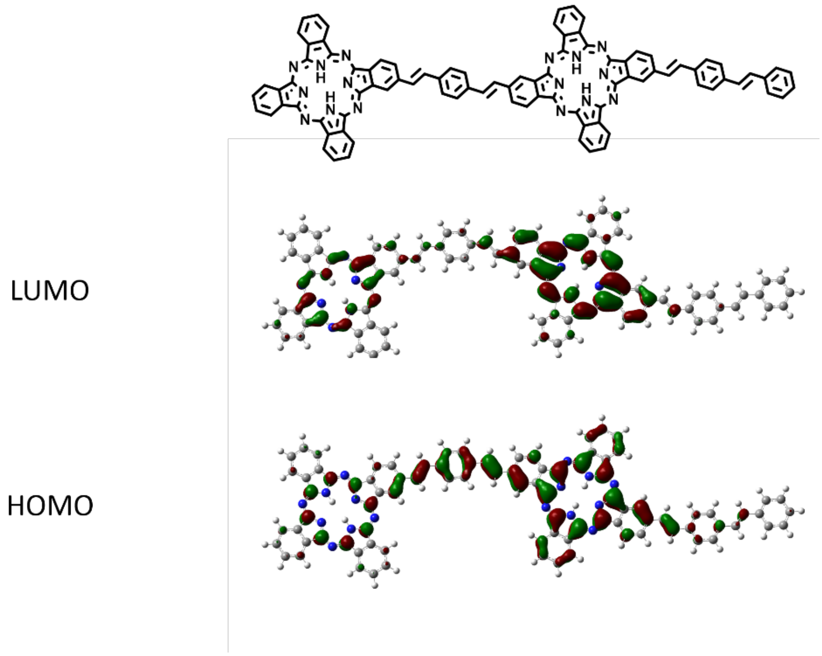

Supplementary Figure 4. Molecular electronic structure of the frontier orbitals of M4.

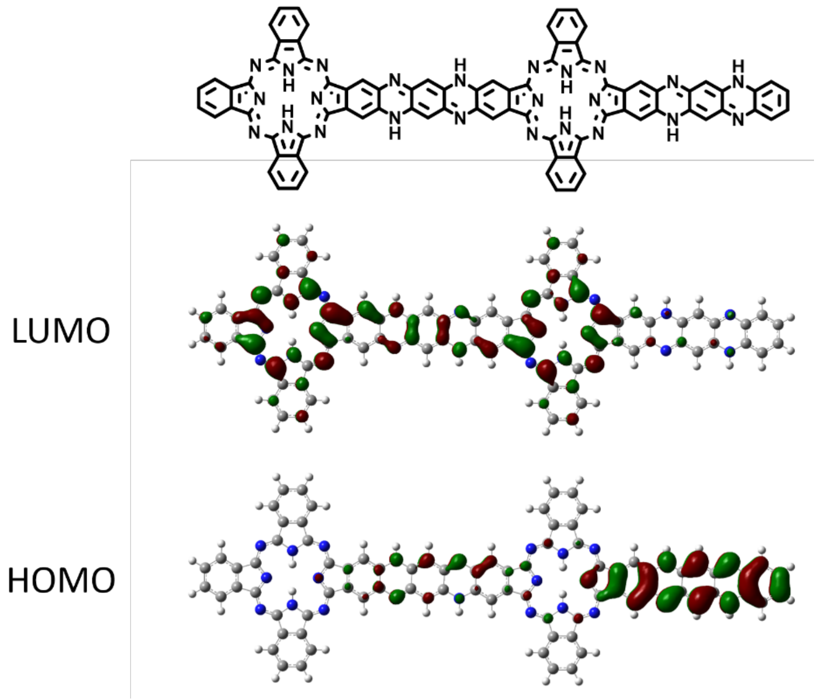

Supplementary Figure 5. Molecular electronic structure of the frontier orbitals of M5.

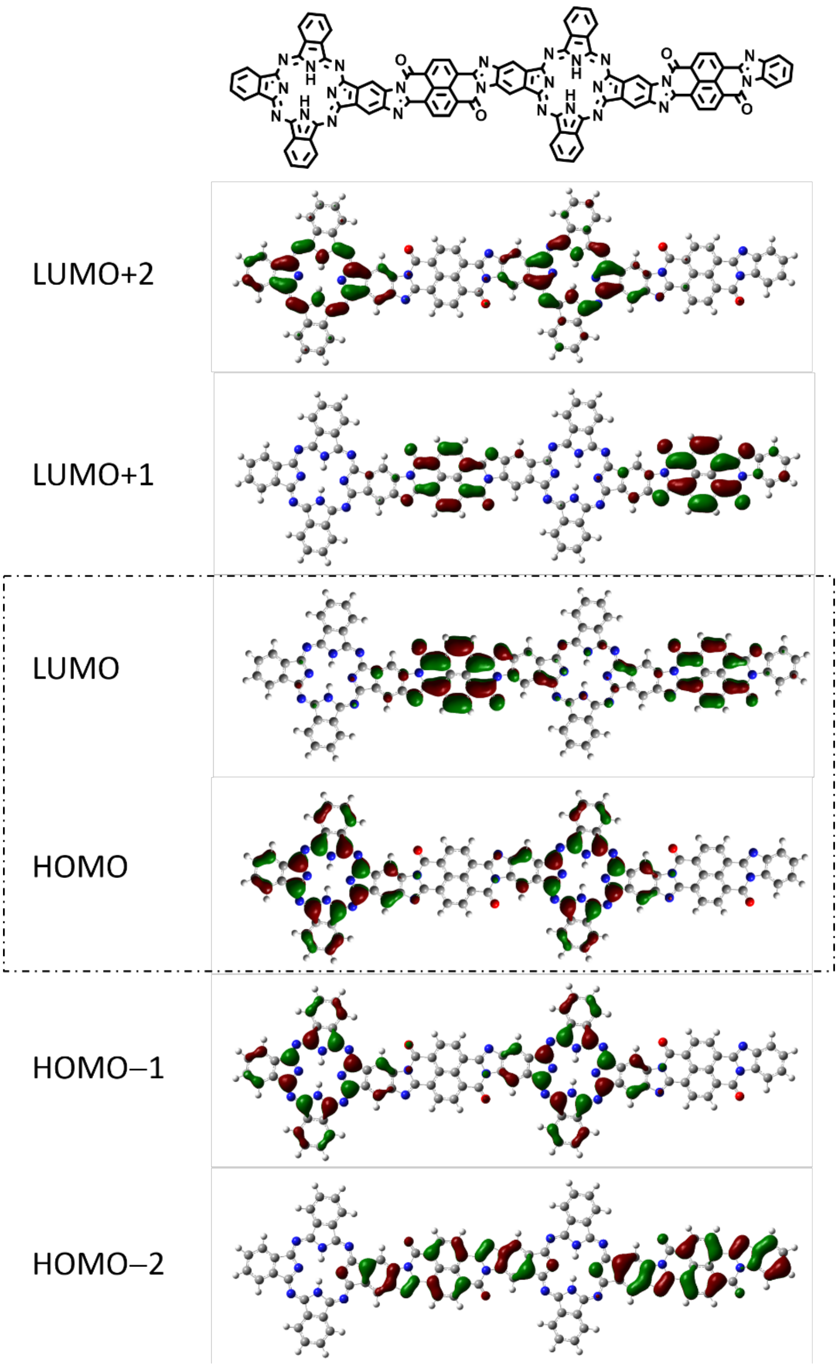

Supplementary Figure 6. Molecular electronic structure of the frontier orbitals of M6.

E_{HOMO} = –5.22 eV, E_{LUMO} = –3.68 eV, $E_{HOMO\text{-}LUMO}$ = 1.54 eV. E_{HOMO-1} = –5.25 eV, E_{HOMO-2} = –6.33 eV, E_{LUMO+1} = –3.61 eV, E_{LUMO+2} = –3.10 eV.

M6-iso1

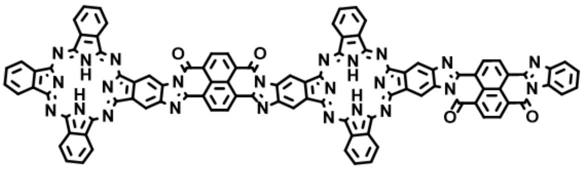

HOMO: -5.21 eV

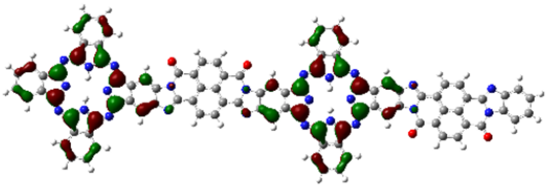

LUMO: -3.70 eV

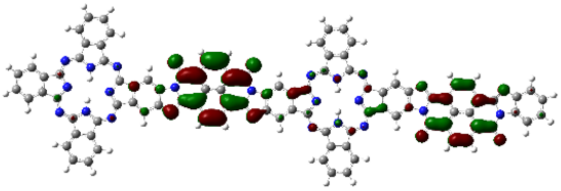

M6-iso2

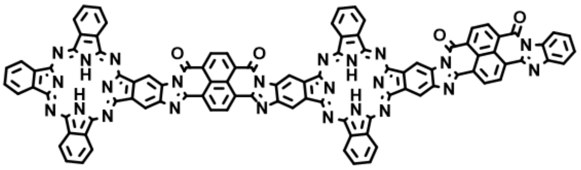

HOMO: -5.21 eV

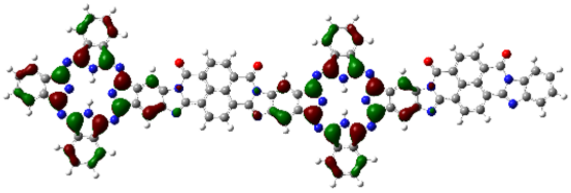

LUMO: -3.70 eV

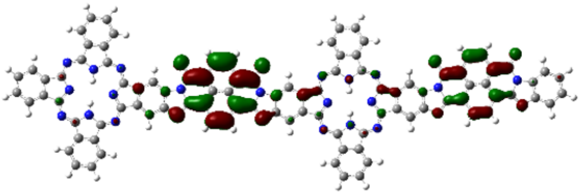

Supplementary Figure 7. Molecular electronic structure of the isomers of M6.

The isomers of **M6-iso1** and **M6-iso2** (vs. **M6**) show a negligible change in their molecular electronic structures and HOMO/LUMO energy levels (−5.21/−5.21 vs. −5.22; −3.70/−3.70 vs. −3.68 eV). In addition, both isomers are higher in energy than **M6** (1.11 kcal/mol for both vs. 0 kcal/mol).

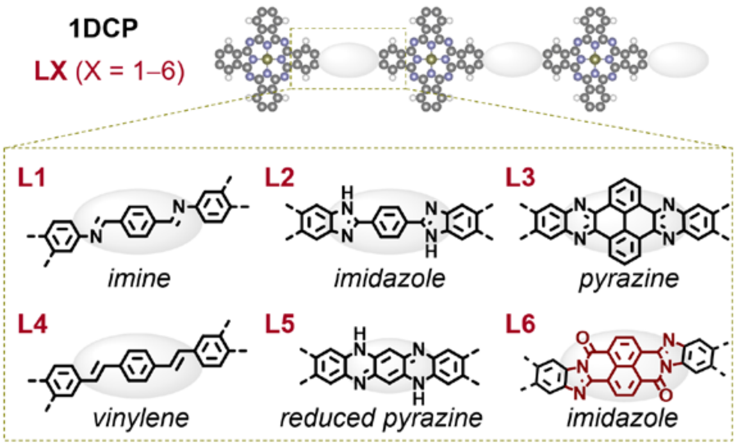

Supplementary Figure 8. Structure of phthalocyanine-based 1DCPs.

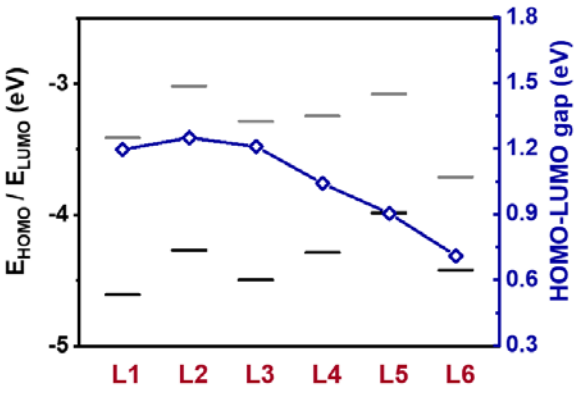

Supplementary Figure 9. HOMO/LUMO energy levels and HOMO–LUMO gap of phthalocyanine-based 1DCPs. Note that the HOMO and LUMO of 1DCPs here refer to the valence band maximum and conduction band minimum, respectively.

Calculations for 1DCPs were performed using the Quantum ESPRESSO 6.7 program[21]. The Generalized gradient approximation (GGA) based on the Perdew–Burke–Ernzerhof (PBE) functional is used for the exchange-correlation energy. The projector augmented wave (PAW) method is used to describe the ionic core pseudopotential. A planewave energy cutoff of 45 Ry are used, and a Monkhorst k-mesh is chosen as 2 × 1 × 1. All structures were fully relaxed until the total force is smaller than 0.0026 eV/Å in the bulk of the 1DCPs.

Compared with **L1–L3** (with energy gap in the range of 1.20–1.25 eV), **L4** and **L5** have diminished energy gaps of 1.04 and 0.90 eV, respectively. It is noted that the BBL-type **L6** shows stabilized LUMO level and thus further narrowed energy gap to 0.71 eV.

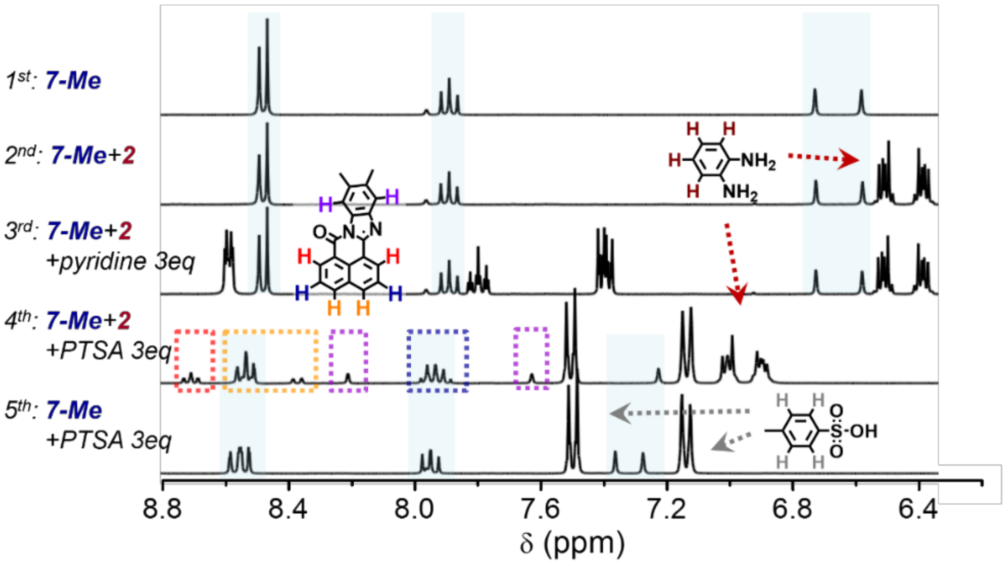

Supplementary Figure 10. ^{1}H NMR spectra for the exchange experiments. The 1st/5th spectra are shown for comparison. Some peaks from **8-Me** are marked by dotted lines.

Consequently, we performed *in-situ* exchange experiments involving **7-Me**, *o*-diamine compound **2** (2 equiv, eq) and catalysts in deuterated DMSO/D_2O (v/v = 500/1) at 90 °C for 12 h to explore the reversibility of the imide-bond formation. No reaction occurred in catalyst-free and pyridine conditions, while four new peaks assignable to **8-Me** emerged in the ^{1}H nuclear magnetic resonance (NMR) spectrum when using acid of PTSA or trifluoroacetic acid (TFA) as catalyst (Supplementary Figures 10–14). This suggests that acid promotes the imidazole ring formation. Nonetheless, no exchange product **7** was detectable. The *ex-situ* exchange experiments between **7-Me** (or **8-Me**) and **2** also led to no exchange reaction (Supplementary Scheme 2), which reveals that both the imide and imidazole formation are irreversible once the dehydration occurs.

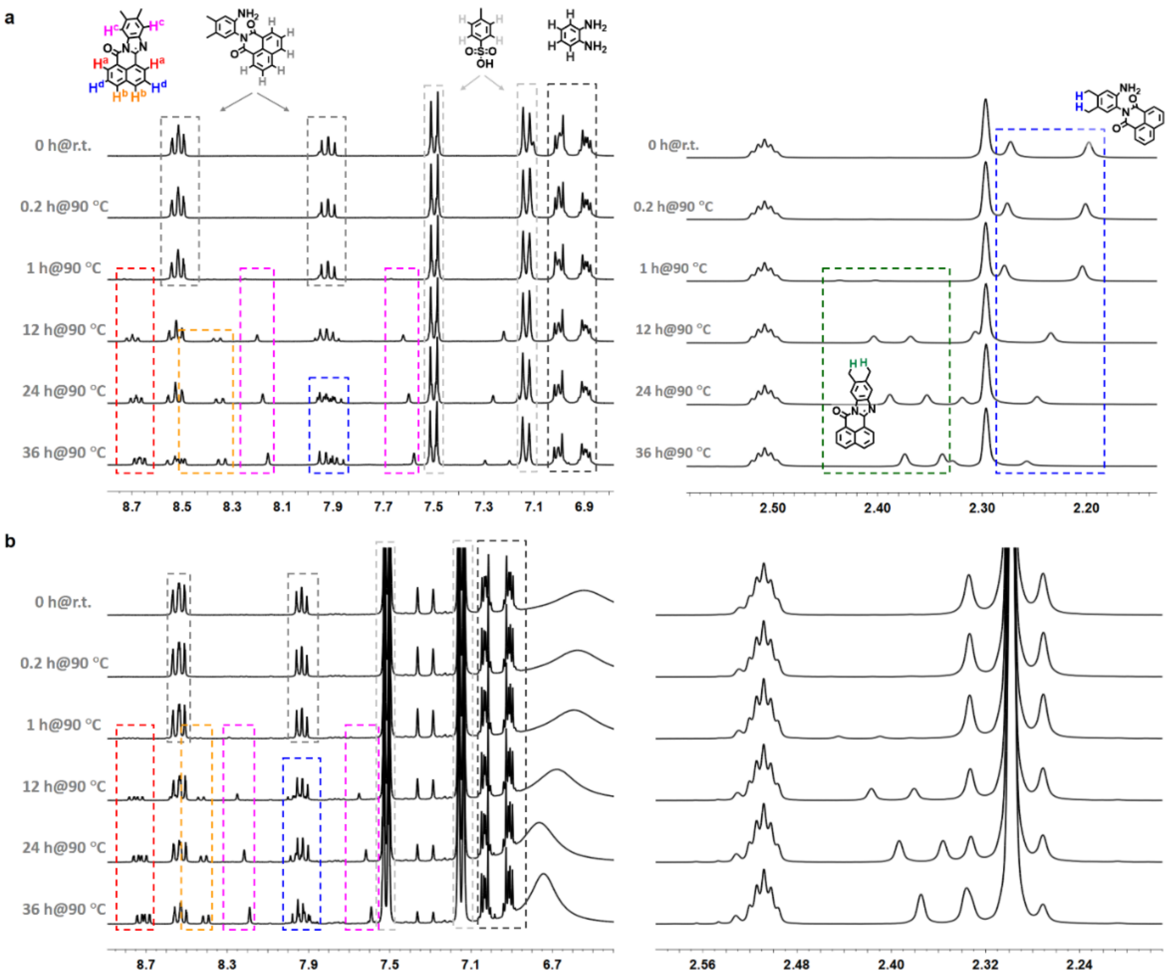

Supplementary Figure 11. Time-dependent *in-situ* ^{1}H NMR spectra of the reaction mixture of 7-Me, 2 and PTSA at 90 °C in DMSO-d$_6$. (a) 3 equiv PTSA. (b) 9 equiv PTSA.

Due to the consumption of free NH_2 via dehydration from **7-Me** to **8-Me**, more PTSA molecules were partially released and then the pH value decreased, which resulted in the shift of the signals for **8-Me**.

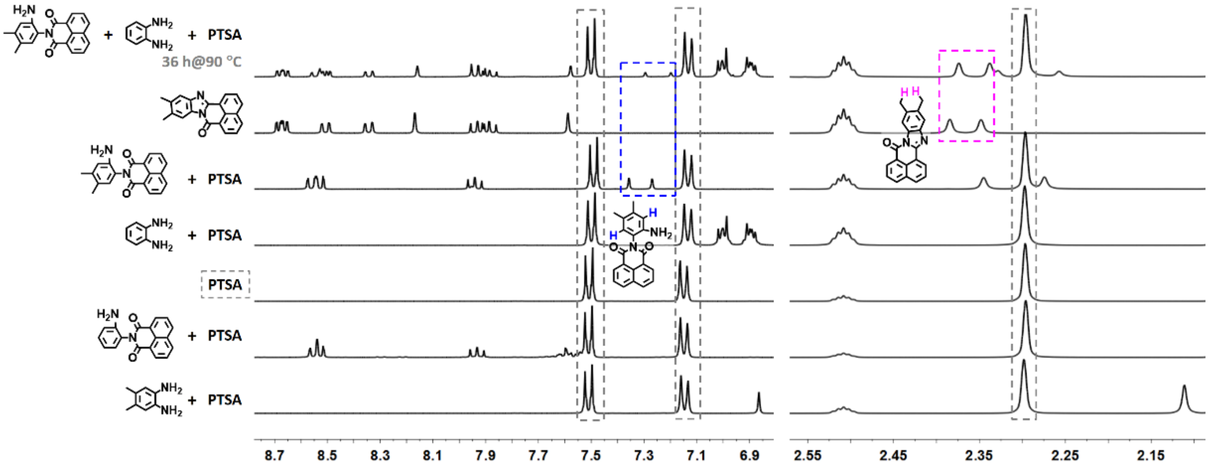

Supplementary Figure 12. Comparison of the ^{1}H NMR spectrum of the reaction mixture of 7-Me, 2 and PTSA at 90 °C for 36 h (36h@90 °C) with selected chemicals in DMSO-d$_6$.

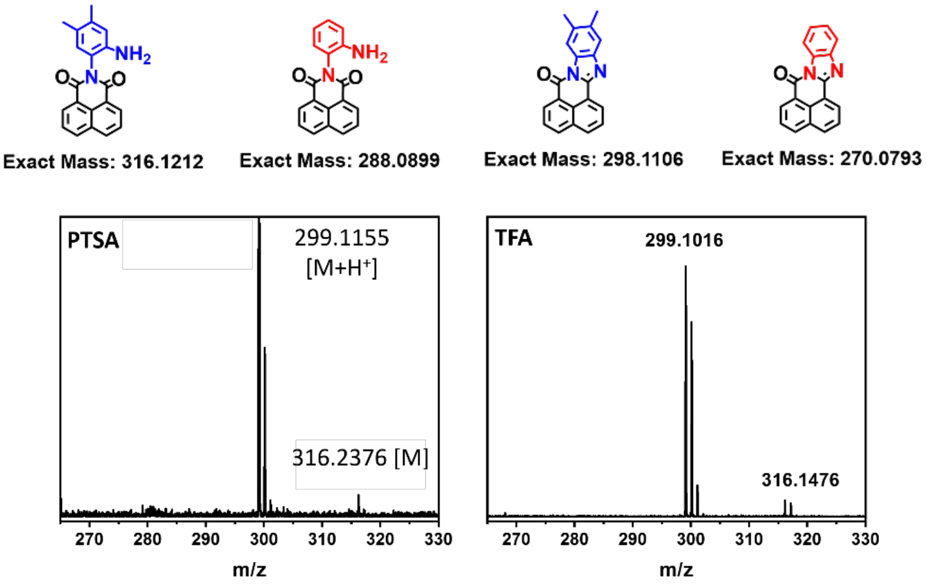

Supplementary Figure 13. MALDI-TOF MS of PTSA- or TFA-catalyzed *in-situ* control experiment at 90 °C for 36 h.

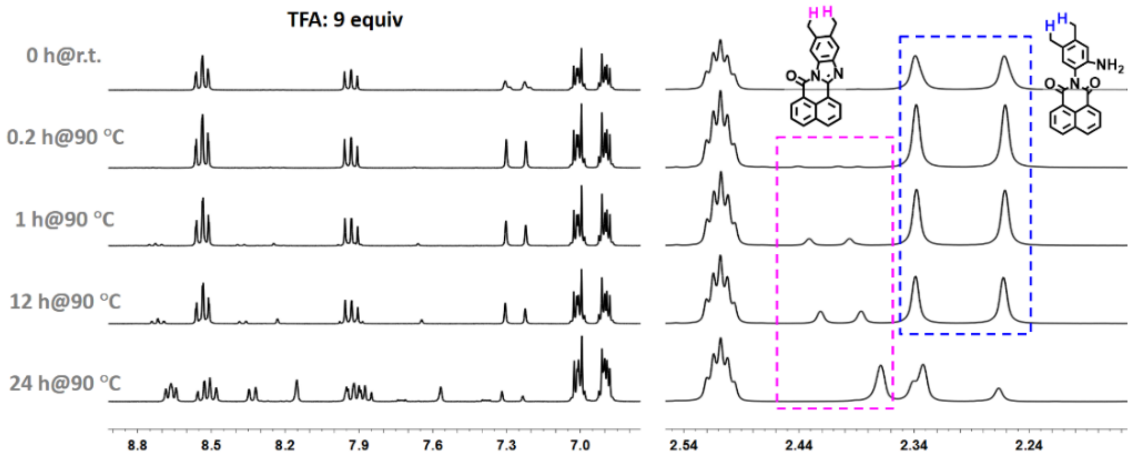

Supplementary Figure 14. Time-dependent *in-situ* ^{1}H NMR spectra of the reaction mixture of 7-Me, 2 and another acid of TFA (9 equiv) at 90 °C in DMSO-d$_6$.

With only 3 equiv TFA, the signal of TFA presents in the aromatic region and influences the analysis of **7-Me** and **8-Me**. Using 9 equiv TFA, signals of Me groups from **8-Me** appeared after 0.2 h, which is faster to the reaction catalyzed by 9 equiv PTSA. **8-Me** precipitated after heating for only 12 h.

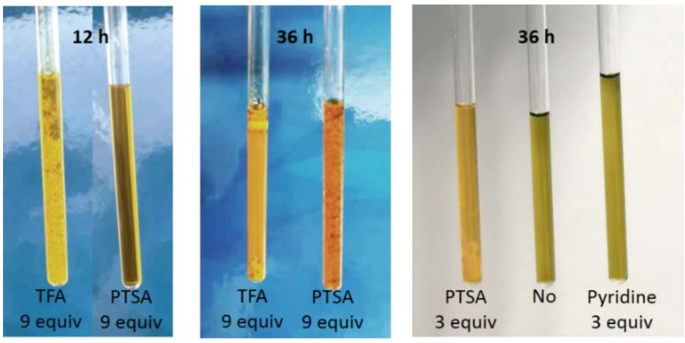

Supplementary Figure 15. Images of the *in-situ* control experiments. 8-Me precipitated after cooling at room temperature.

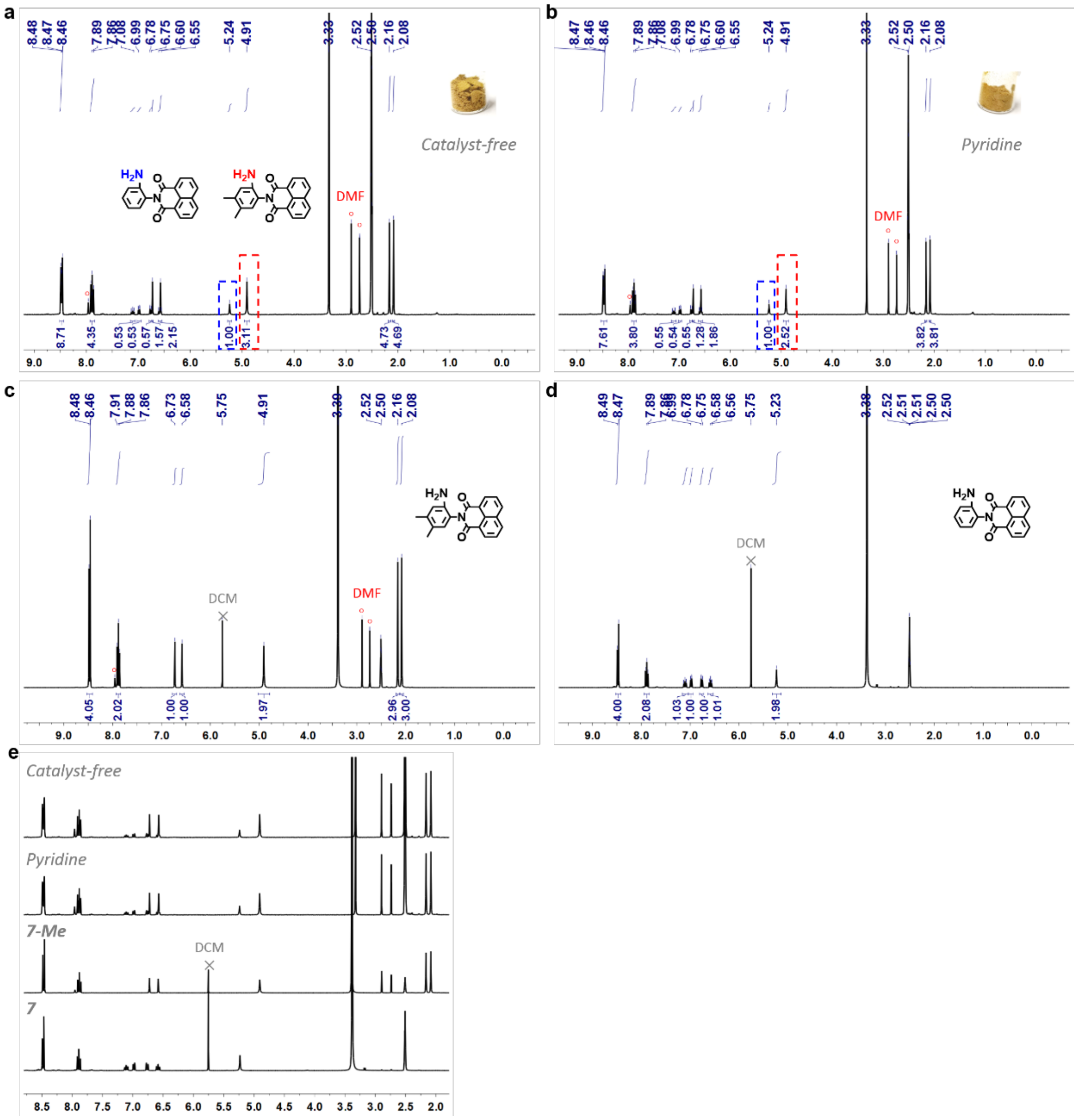

Supplementary Figure 16. ^{1}H NMR spectra of the product obtained from competitive reaction without catalyst (a) or with pyridine as catalyst (b), 7-Me (c) and 7 (d) in DMSO-d$_6$ as well as the spectra for comparison (e). Selected peaks for calculating the ratio between **7-Me** and **7** are marked by red and blue colors, respectively.

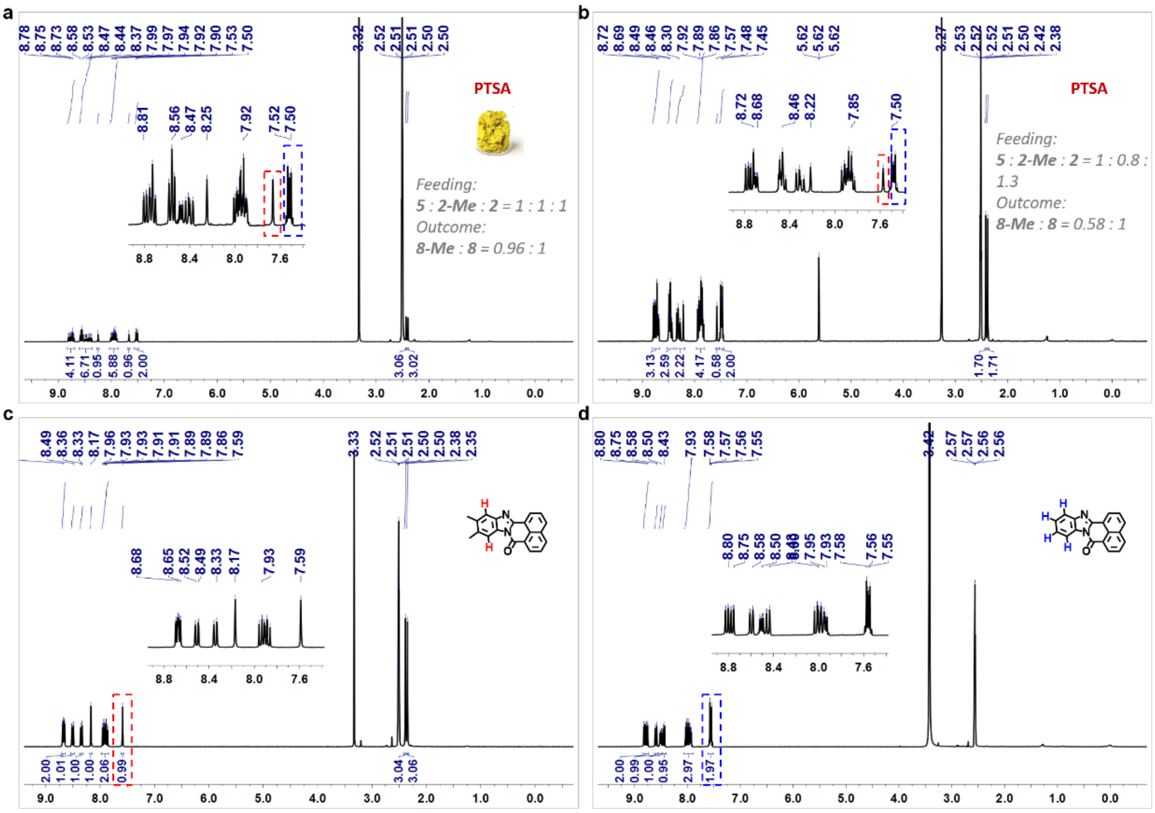

Supplementary Figure 17. ^{1}H NMR spectra of the product obtained from competitive reaction with PTSA (a,b) as catalyst, 8-Me (c) and 8 (d) in DMSO-d_6. Selected peak for calculating the ratio between **8-Me** and **8** are marked by red and blue colors, respectively.

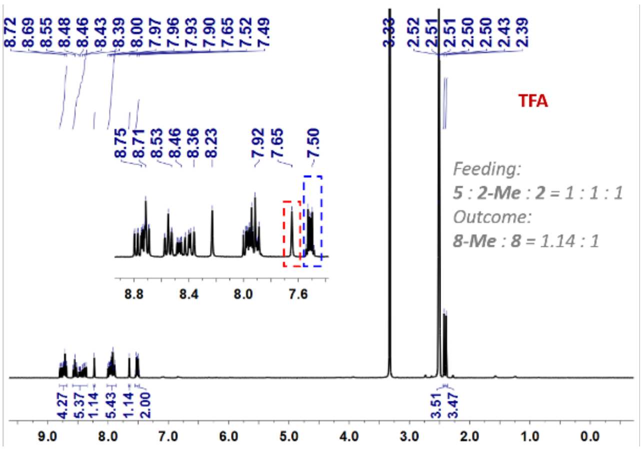

Supplementary Figure 18. ^{1}H NMR spectrum of the product obtained from competitive reaction with TFA as catalyst in DMSO-d_6. Selected peak for calculating the ratio between **8-Me** and **8** are marked by red and blue colors, respectively.

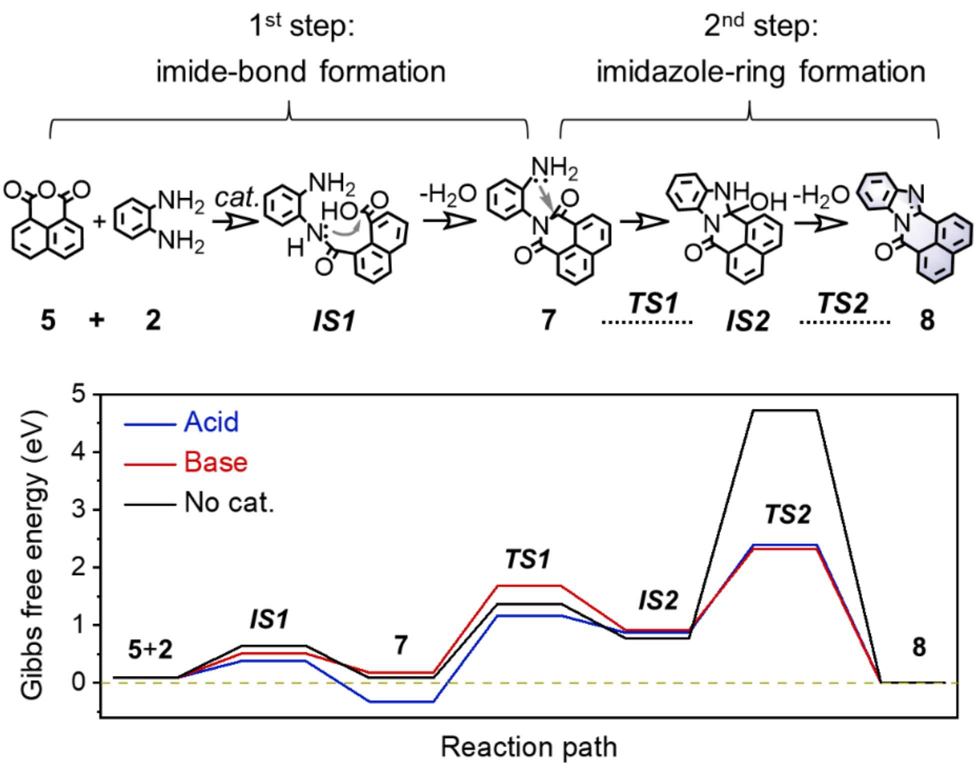

Catalyst	Gibbs free energy (eV)						
	**5 + 2**	***ISI***	**7**	***TS1***	***IS2***	***TS2***	**8**
No	0.09099	0.64186	0.08218	1.36724	0.77664	4.72118	0
Pyridine	0.09099	0.50861	0.1778	1.6787	0.91817	2.31392	0
PTSA	0.09099	0.37786	-0.32673	1.16302	0.87125	2.38576	0

Supplementary Figure 19. Reaction mechanism and calculated Gibbs free energies of the imidazole ring formation. *IS*: intermediate state. *TS*: transition state. The energies of **8** were used as references (0 eV), because **8** has almost no interaction with the employed basic or acidic catalyst, while the energy of **2** is significantly affected by the catalyst.

Although very few reports have shown that an organic acid (i.e. acetic acid) as solvent could promote this reaction,[20] its role and catalytic efficiency remain unexplored. DFT calculations are conducted to investigate the role of different catalysts in the condensation reaction. The energy barriers on the forward way towards **7** can be easily overcome by thermal fluctuations in all conditions. Next, transition state ***TS1*** (Supplementary Figure 23) appears, which is essential to trigger the cyclization of **7** thus forming the ring-closure

intermediate state ***IS2***. Notably, PTSA can stabilize all these states via protonation of the $–NH_2$ group, which results in an energetically more favorable ***TS1*** (1.16 eV) than that in catalyst-free or pyridine condition (1.38 or 1.68 eV). This indicates that PTSA stabilizes the intermediate/transition states with lower energies and benefits the formation of imidazole ring with enhanced reactivity due to the unique protonation-deprotonation ability of PTSA. In regard to the final dehydration from ***IS2*** to **8**, the 2nd transition state ***TS2*** with a substantially high energy (> 2.3 eV) is formed, indicative of the necessity of a high temperature for this step.

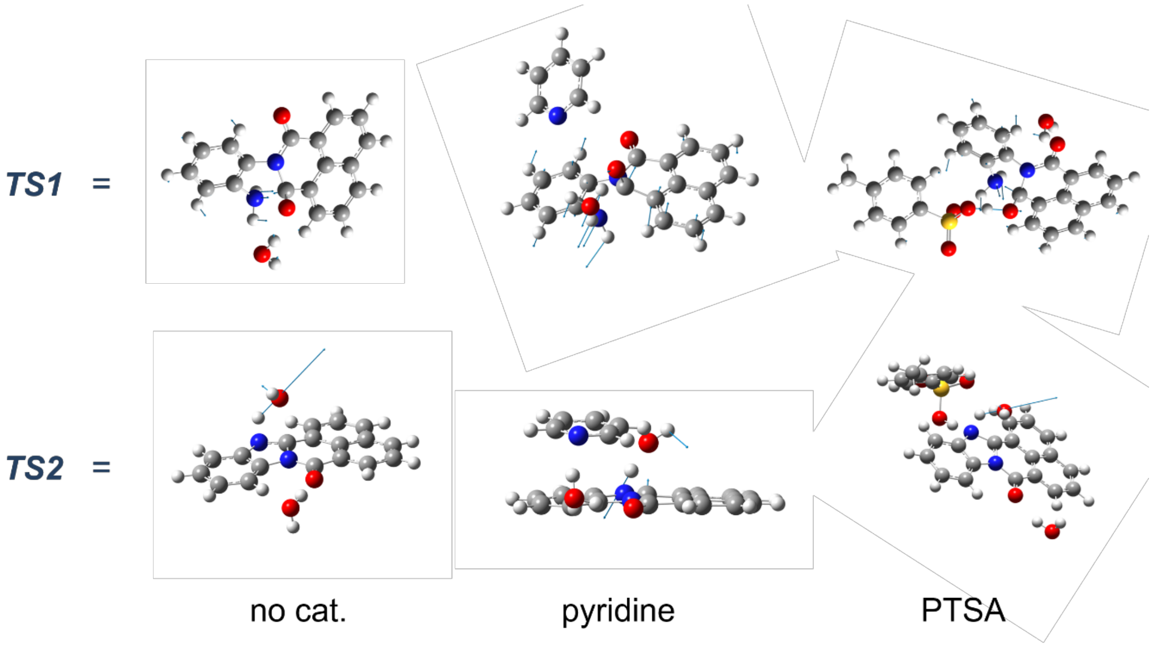

Supplementary Figure 20. Calculated structures of the transition states.

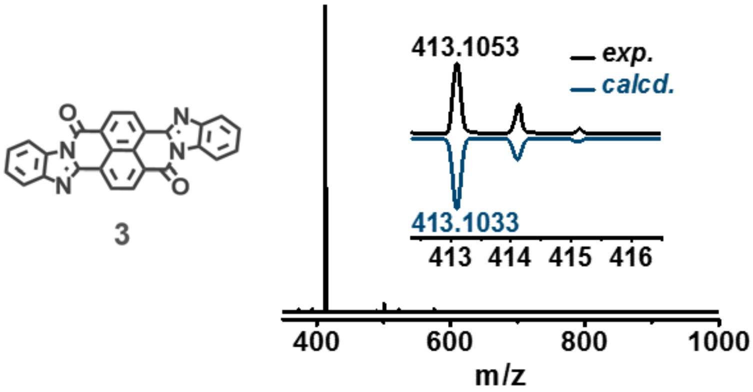

Supplementary Figure 21. High-resolution MALDI-TOF MS spectrum of 3.

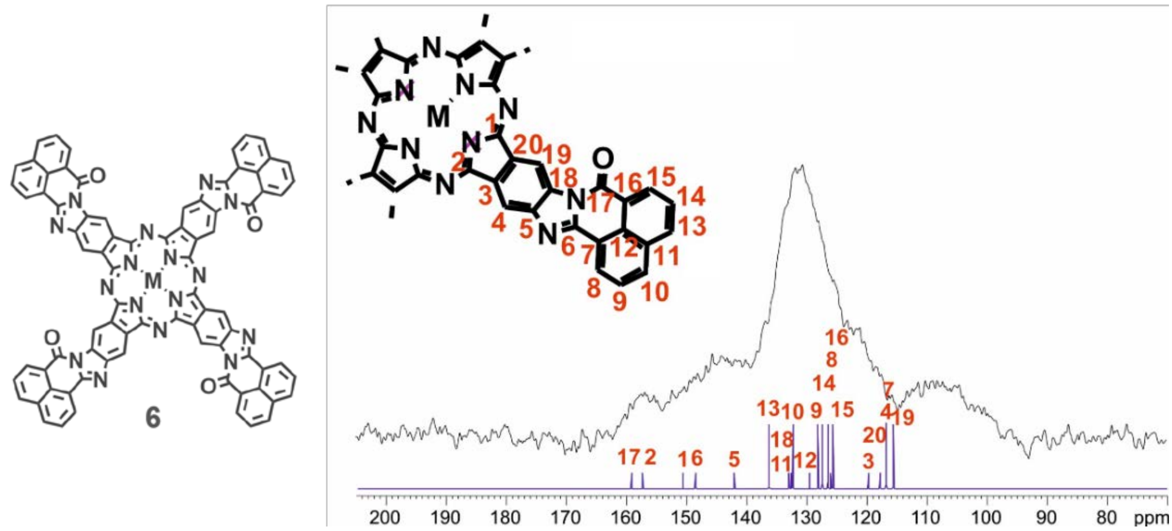

Supplementary Figure 22. Solid-state ^{13}C NMR spectrum of the model compound 6 (with M = Zn)**.** The program ACD/Labs was used for the prediction.[22]

6 was not detected by MALDI-TOF MS, which suggests that such a large and rigid ring-fused compound is not able to fly during the measurement. It's noted that the synthesized 2DCP (**2DCP-ZnPc**) from **7-Zn** was barely crystalline, which can be ascribed to the catalytic ability of Zn^{2+} in this condensation reaction[19] to disturb the interlayer arrangement of phthalocyanines.

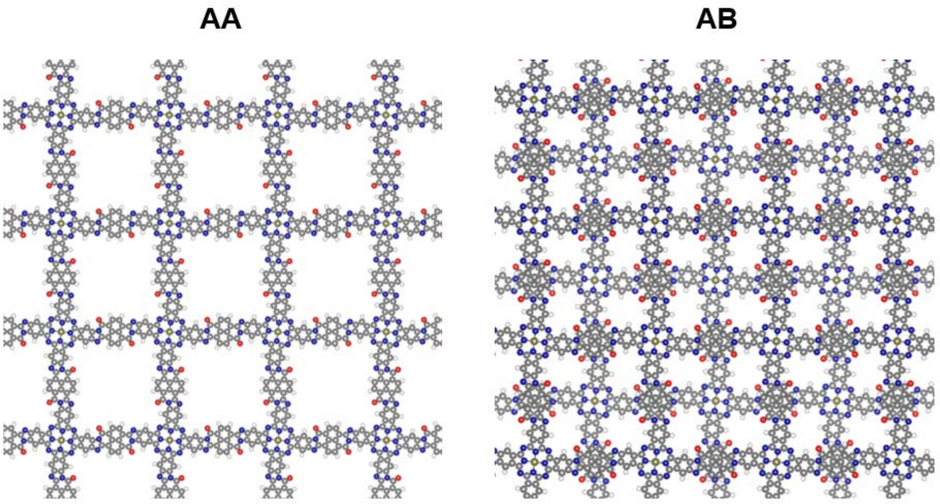

Supplementary Figure 23. 2DCP-CuPc with AA and AB stacking geometries.

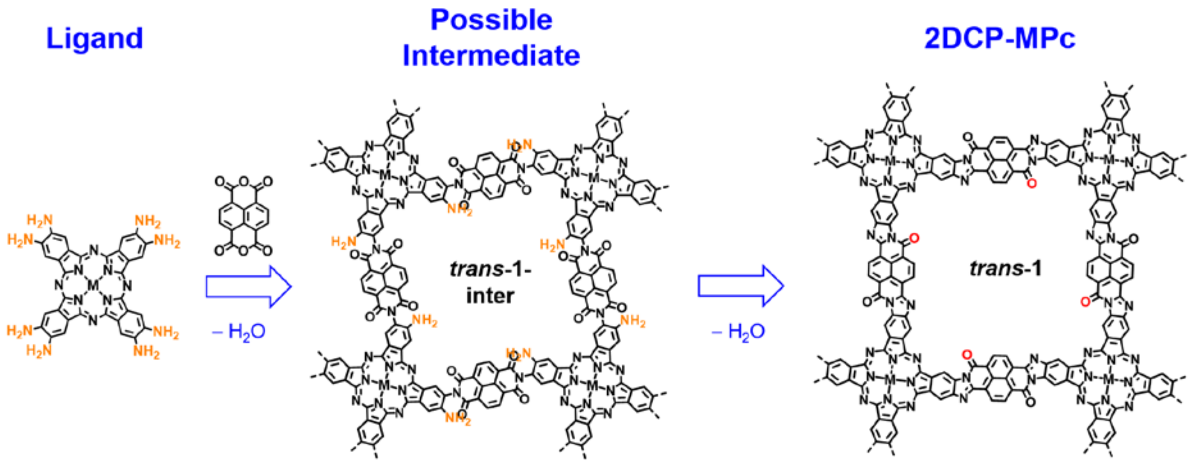

Supplementary Figure 24. Reaction pathway towards the formation of 2DCP-MPc (termed as *trans*-1, shown in Figure 2a).

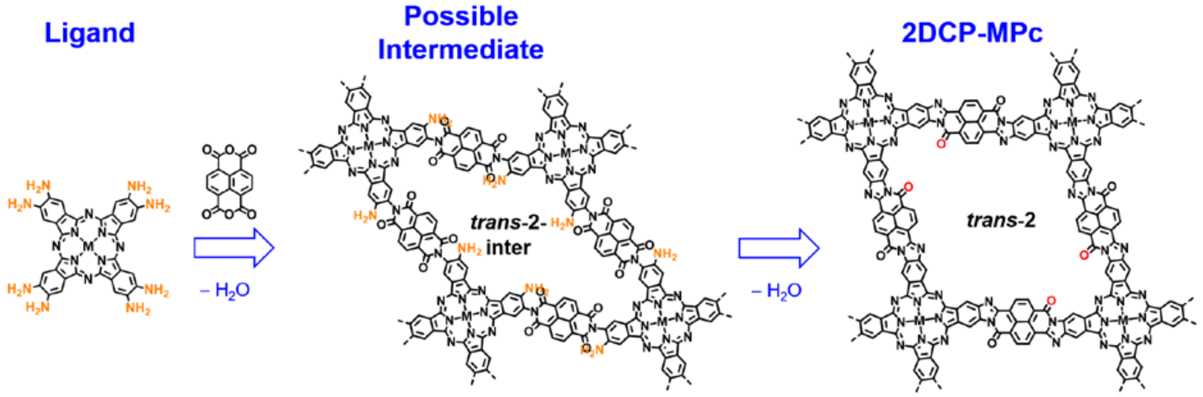

Supplementary Figure 25. Reaction pathway towards the formation of isomeric 2DCP-MPc (*trans*-2).

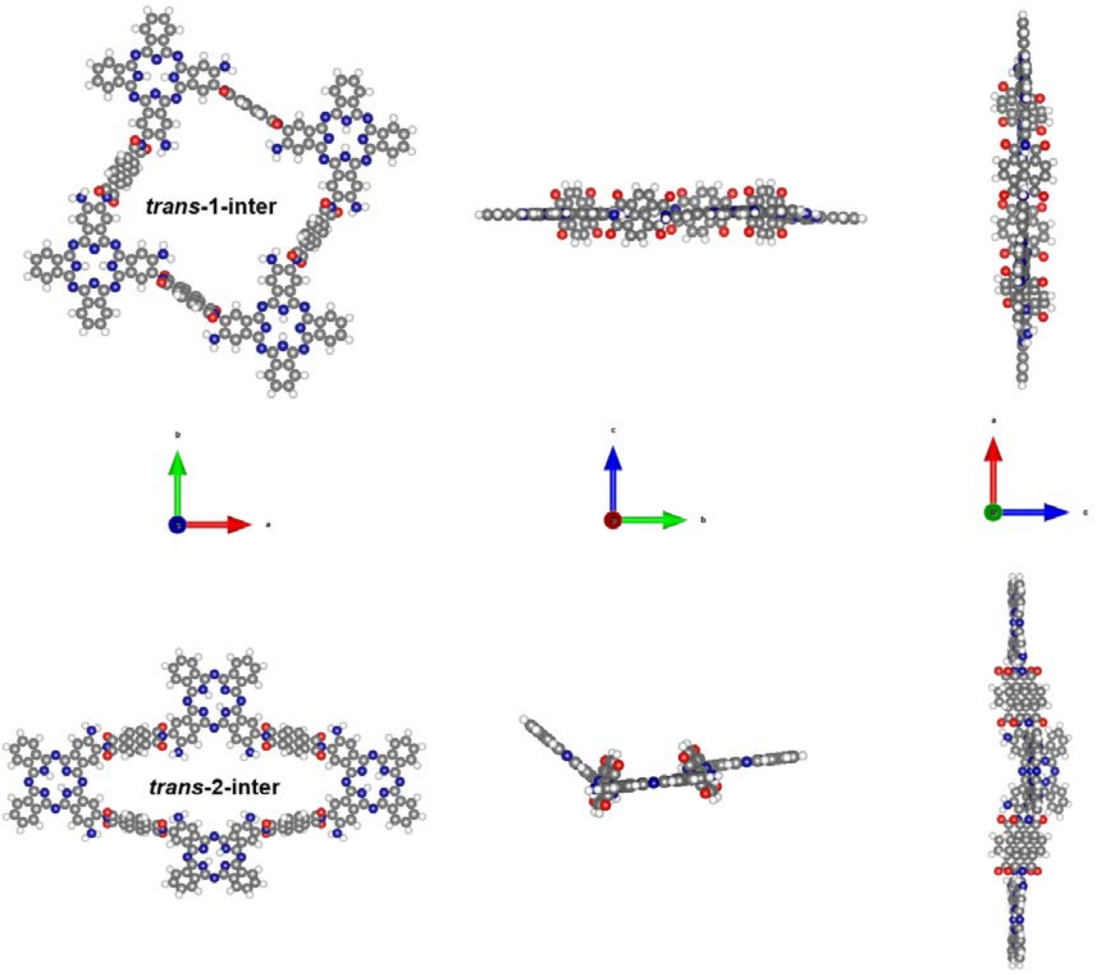

Supplementary Figure 26. Representative one-pore structural models of *trans*-1-inter and *trans*-2-inter to reveal the intralayer arrangement. The interaction between $-NH_2$ and PTSA is not considered.

Note that ***trans*-2-inter** possesses significantly lower symmetry than ***trans*-1-inter**. This means that ***trans*-2-inter** and ***trans*-2** represent, in principle, only ideal periodic structures.

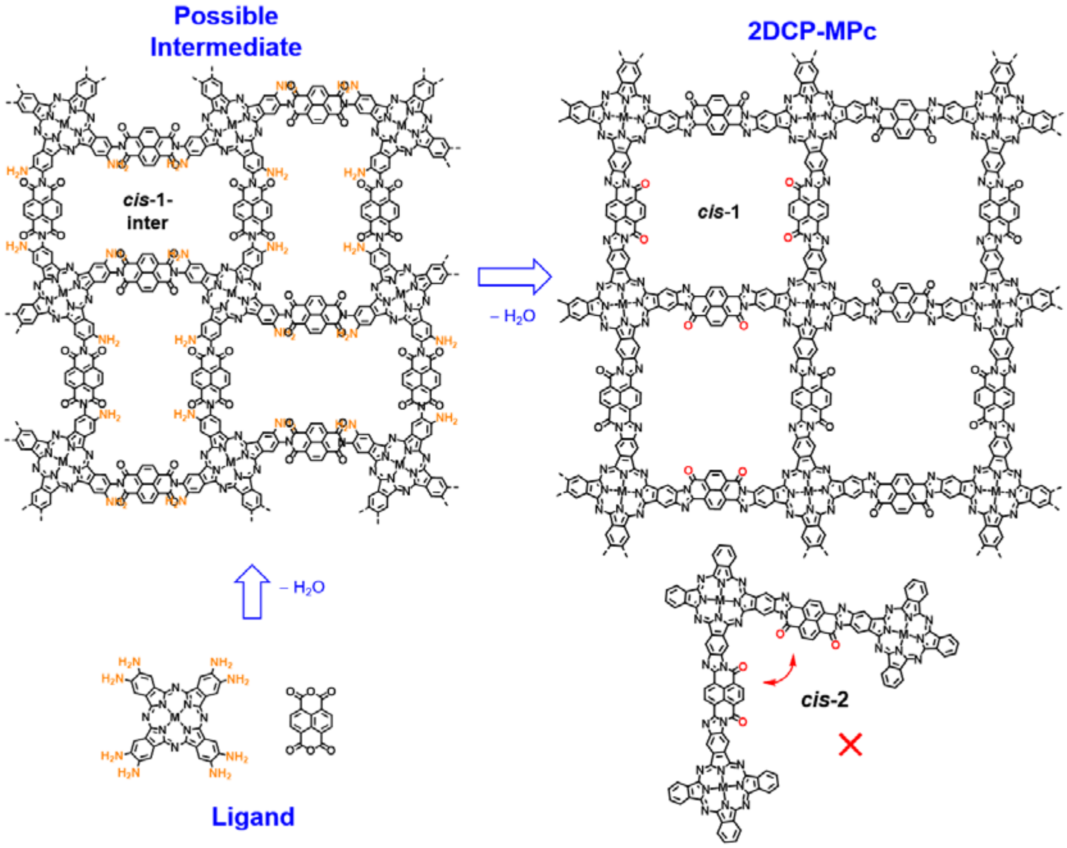

Supplementary Figure 27. Reaction pathway towards the formation of isomeric 2DCP-MPc (*cis*-1). Note that ***cis*-1-inter** possesses even lower symmetry than ***trans*-2-inter**.

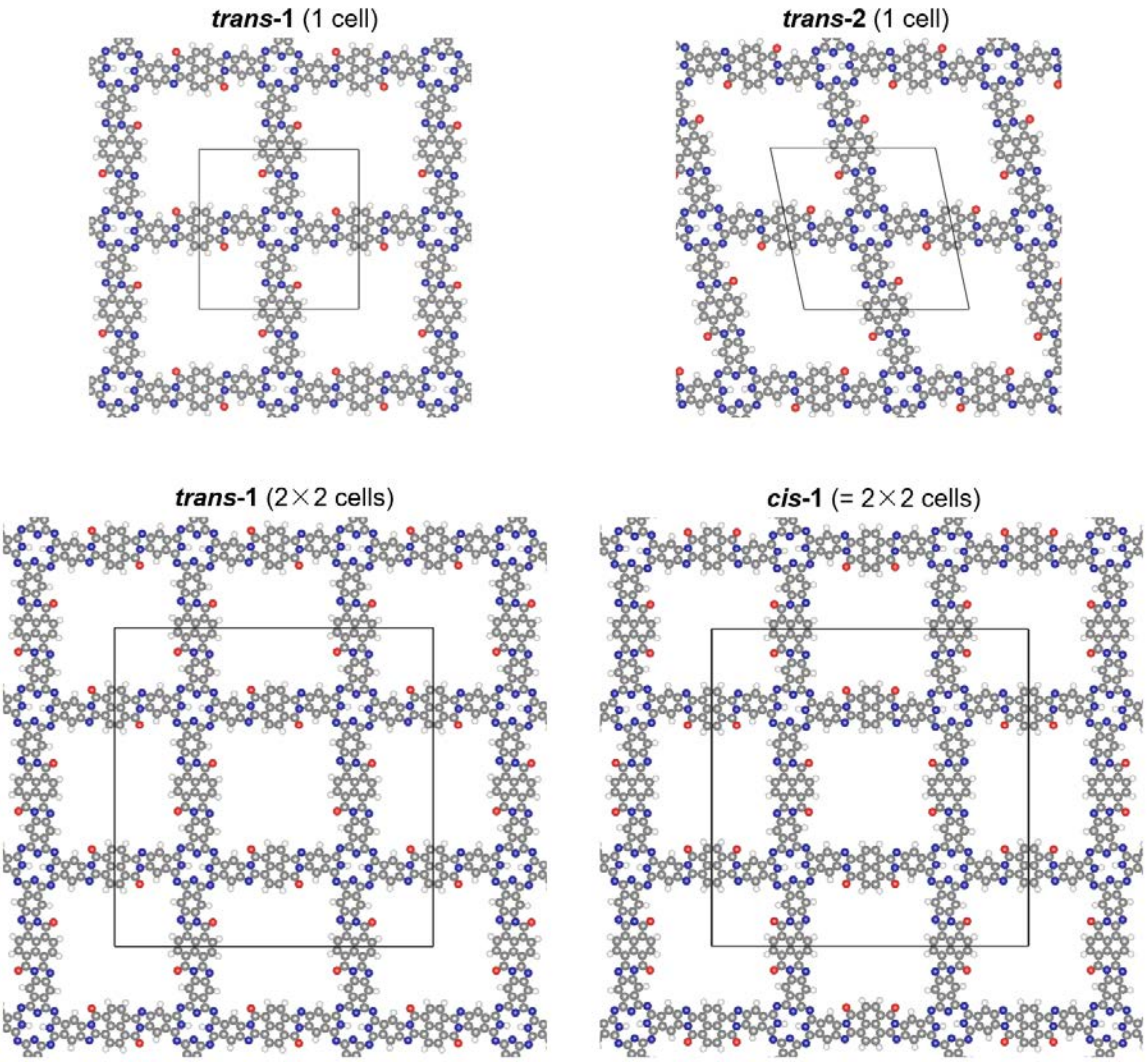

Supplementary Figure 28. Structure models of the metal-free *trans*-1, *trans*-2, and *cis*-1 monolayers. The unit cell is displayed in the black frame.

The condensation of *o*-diamine and anhydride towards imidazole ring formation adopts a two-step dehydration process, i.e., imide-bond formation and subsequent intramolecular dehydration, while the latter step has a significantly larger energy barrier than the former one (Supplementary Figure 19). Therefore, an imide-intermediate (termed as ***trans*-1-inter**, ***trans*-2-inter** or ***cis*-1-inter** shown in Supplementary Figures 24, 25 and 27) forms prior to the formation of **2DCP-MPc** during the 2D polycondensation between **4-M** and **1**. The steric hindrance between adjacent $-NH_2$ and $-C{=}O$ and the interaction (Supplementary Figure 26) between $-NH_2$ and PTSA (Supplementary Figure 20) influence both the intra- and inter-layer

arrangement of the imide-intermediate thus resulting in only one type of **2DCP-MPc** (***trans*-1**, ***trans*-2**, or ***cis*-1**) for each intermediate after the dehydration.

The formation of ***trans*-2-inter** or ***cis*-1-inter** apparently possesses a lower possibility than ***trans*-1-inter** due to the low symmetries. They represent, in principle, only ideal periodic structures (Supplementary Figure 26). However, we could not exclude the formation of trace ***trans*-2-inter** and ***cis*-1-inter** (thus ***trans*-2** and ***cis*-1**) during the polycondensation, which can happen at the edges of ***trans*-1** to bridge different crystal domains.

To investigate their energetic stability, we conducted DFT calculations using monolayers of the metal-free ***trans*-1, *trans*-2, and *cis*-1**. This simplifies the computation, as the unit cell of ***cis*-1** contains four times the number of atoms of the unit cell of ***trans*-1** (and the existence of metal leads to additional computational work). The results indicate that ***trans*-1** and ***trans*-2** (compared on the 1×1 cell level, shown in the upper figures in Supplementary Figure 28) are isoenergetic with a negligible energy difference of ~0.02 eV, while ***cis*-1** lays higher in energy than ***trans*-1** or ***trans*-2** (compared on the 2×2 cells level, shown in the lower figures in Supplementary Figure 28) by ~0.38 eV (8.73 kcal/mol). This is consistent with the calculated result for model compound **M6** in Supplementary Figure 7: that is, the *trans*-isomer is energetically more stable than the *cis*-one.

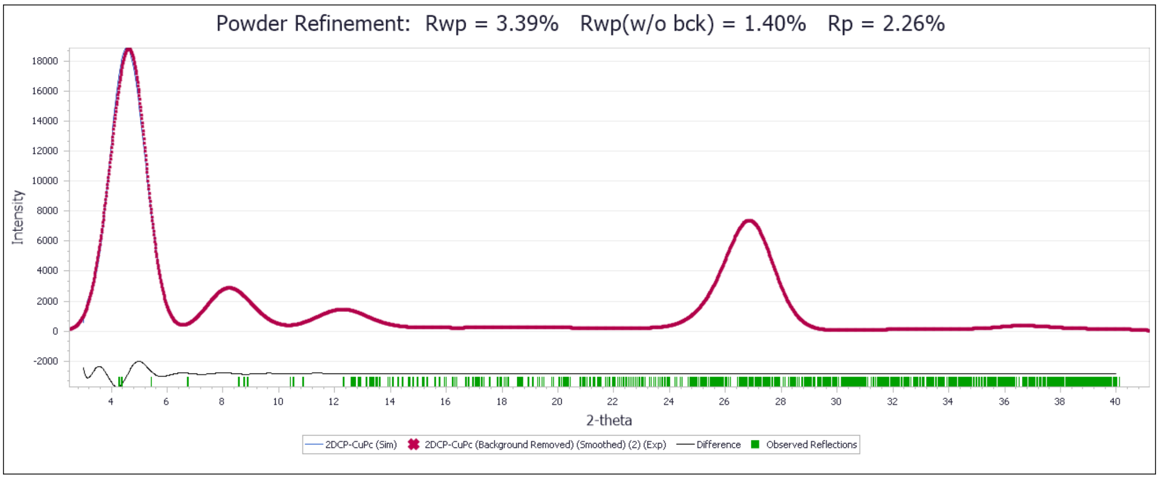

Supplementary Figure 29. Pawley refinement of 2DCP-CuPc. Experimentally observed PXRD pattern was reproduced by Pawley refinement method with tiny difference (R_{wp} =

3.39 %, R_p = 2.26%;).

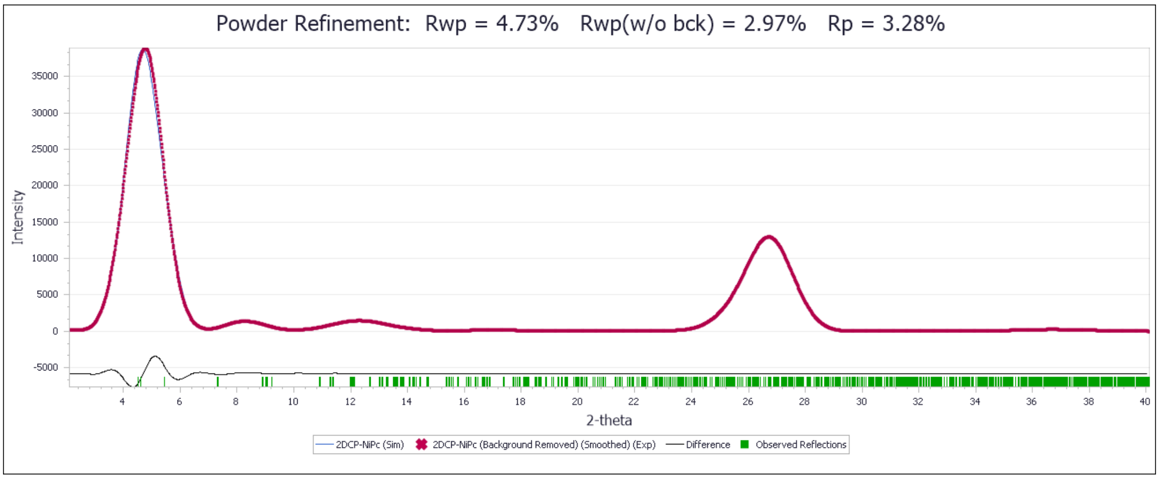

Supplementary Figure 30. Pawley refinements of 2DCP-NiPc. Experimentally observed PXRD pattern was reproduced by Pawley refinement method with tiny difference (R_{wp} = 4.73%, R_p = 3.28%).

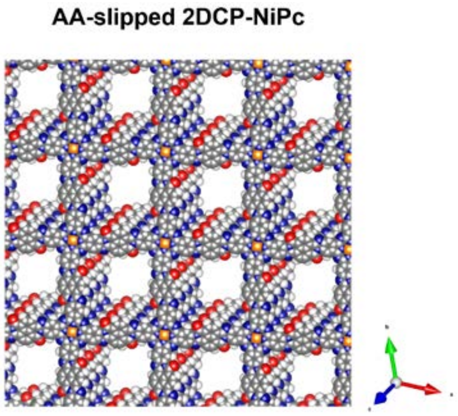

Supplementary Figure 31. AA-slipped stacked 2DCP-NiPc. The PXRD pattern presents (100) peak at 4.72°, which slightly differs from that of **2DCP-CuPc** at 4.62°,

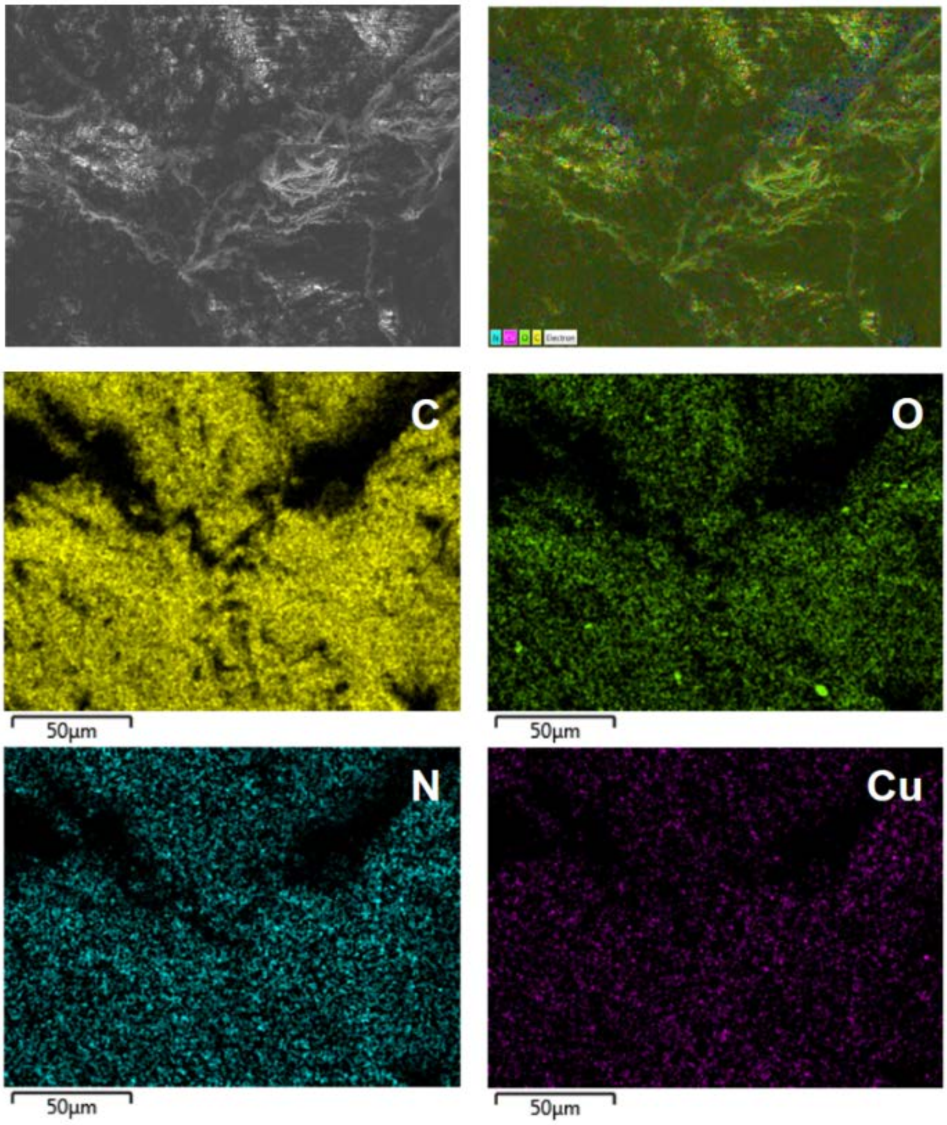

Supplementary Figure 32. Energy dispersive X-ray (EDX) spectroscopy of the elemental mapping images of 2DCP-CuPc.

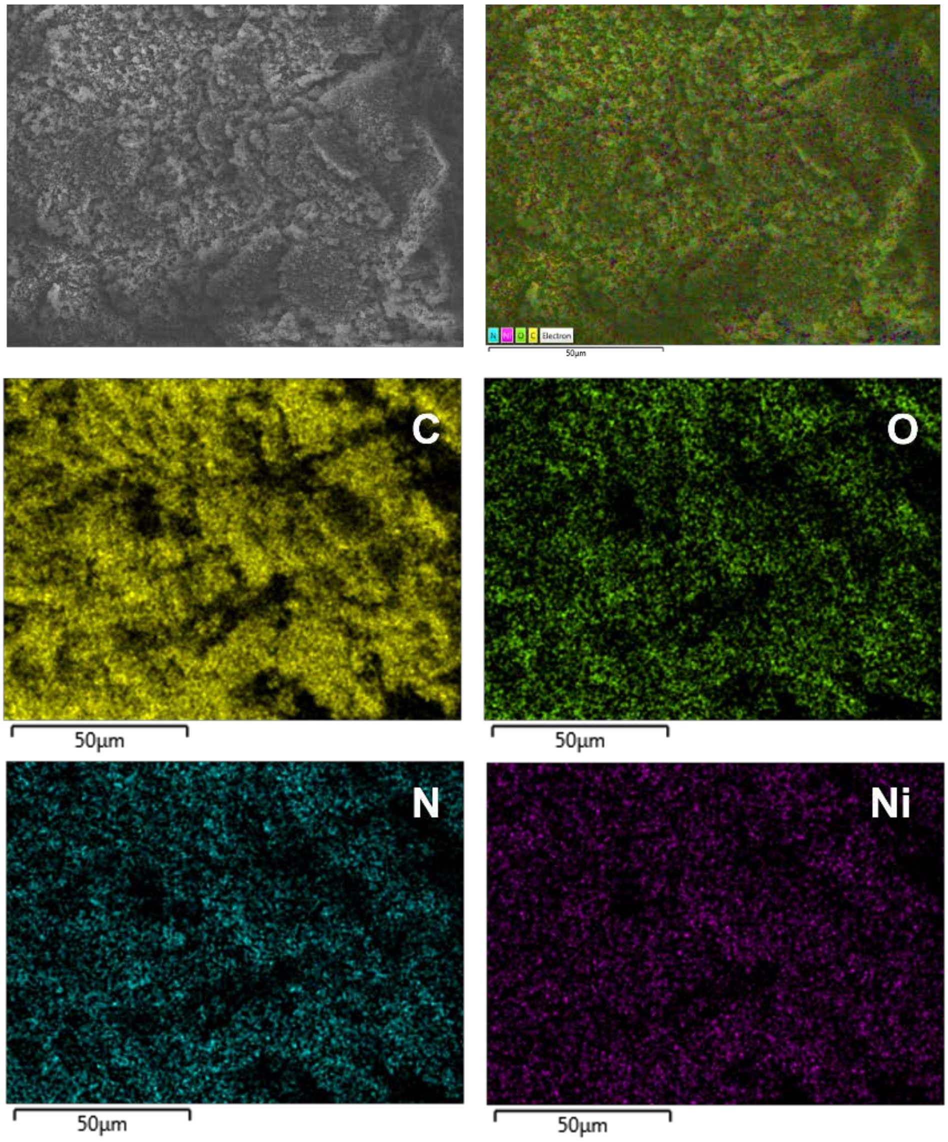

Supplementary Figure 33. Energy dispersive X-ray (EDX) spectroscopy of the elemental mapping images of 2DCP-NiPc.

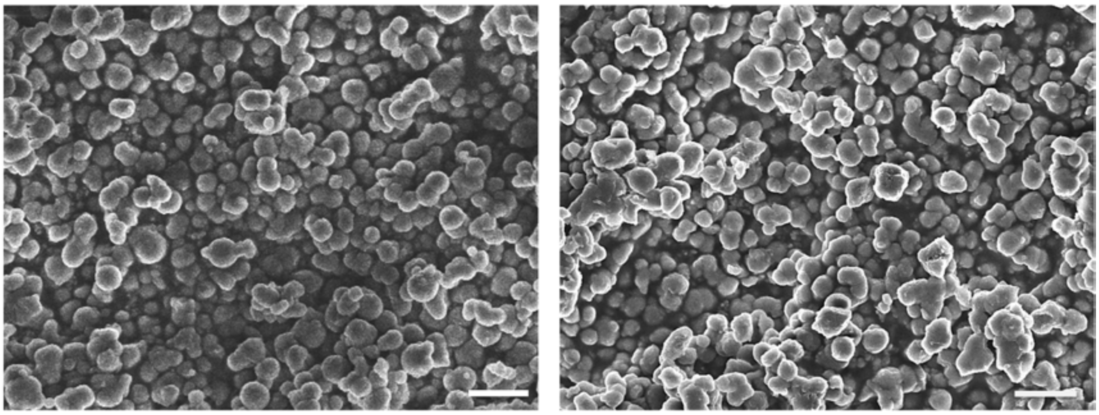

Supplementary Figure 34. SEM images of 2DCP-NiPc (left) and 2DCP-CuPc (right). Scale bar = 1 μm.

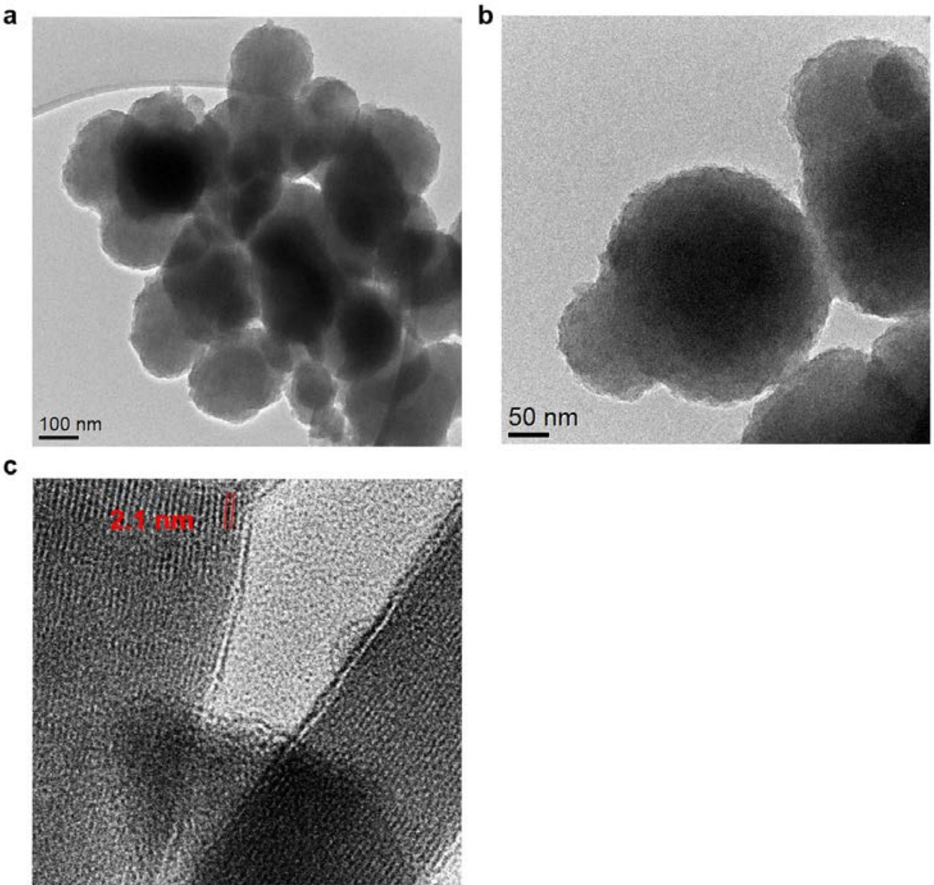

Supplementary Figure 35. (a,b) TEM images of 2DCP-CuPc and 2DCP-NiPc, respectively. (c) High-resolution TEM image of 2DCP-CuPc. Scale bar = 60 nm. Note that only the small 2DCP crystals can be easily dispersed via sonication in solvent to prepare the sample on TEM grid.

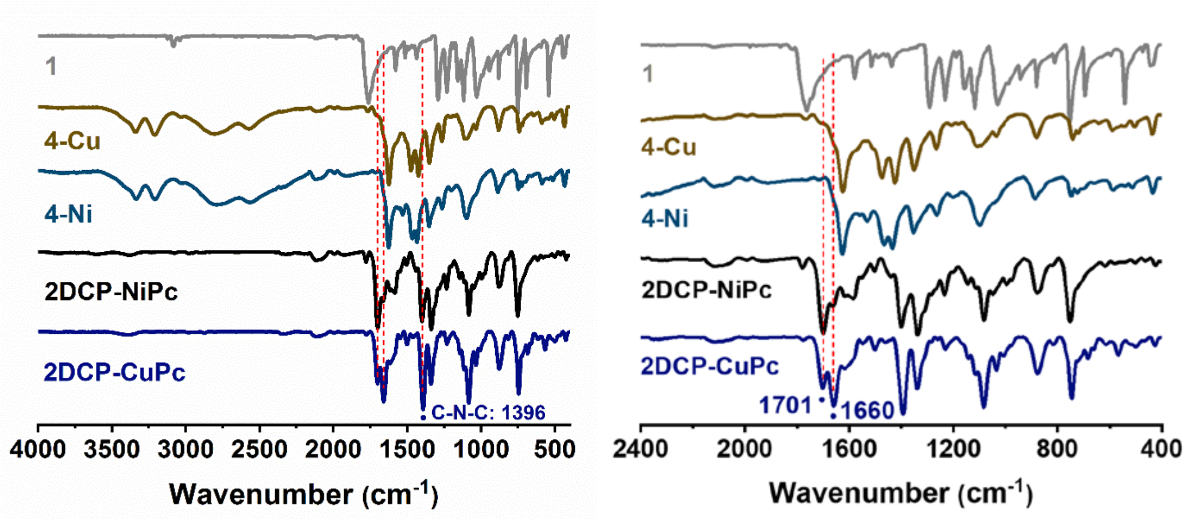

Supplementary Figure 36. FT-IR spectra in 400–4000 cm^{-1}.

The peaks at 1396 cm^{-1} in **2DCP-MPcs** are attributed to the stretching vibration of the C–N–C moiety.[23] The tiny peak at ca. 1780 cm^{-1} could be assigned to the unreacted carbonyls (C=O) at the edges.

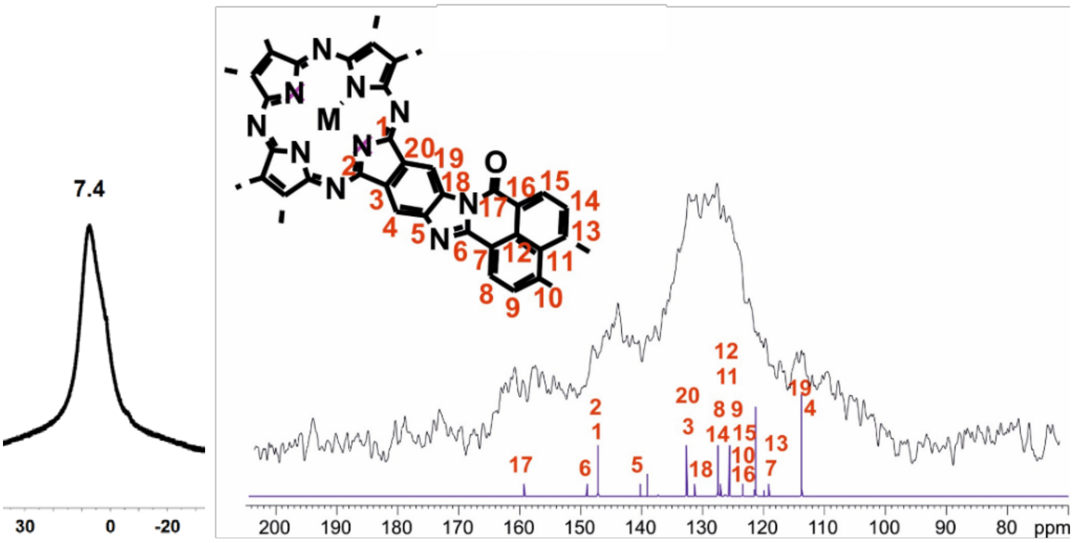

Supplementary Figure 37. Solid-state ^{1}H NMR (left) and experimental as well as predicted ^{13}C NMR (right) spectra of 2DCP-NiPc. The program ACD/Labs was used for the prediction.[22]

^{13}C cross-polarization magic-angle spinning (CP-MAS) NMR spectrum displays four signals at 109, 129, 144 and 159 ppm, which are in good agreement with the predicted

spectrum.

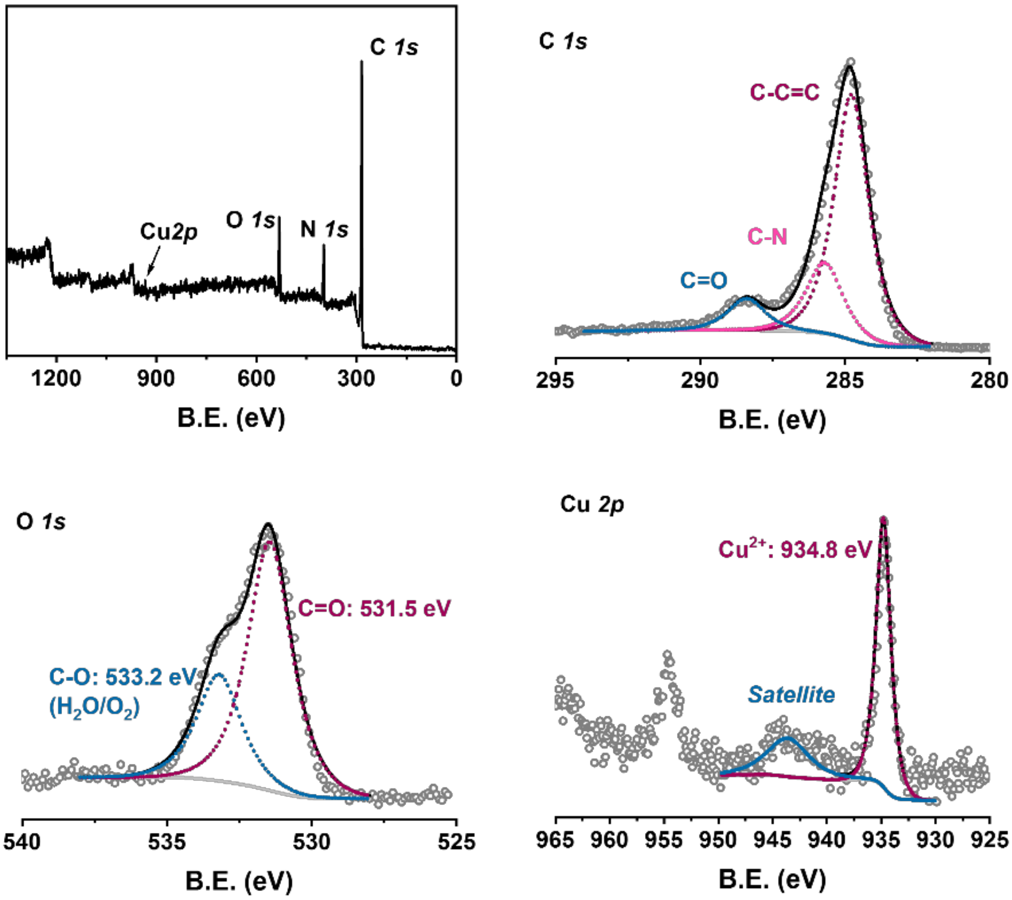

Supplementary Figure 38. XPS survey spectrum and the high-resolution C 1*s*, O 1*s*, and Cu 2*p* spectra of 2DCP-CuPc.

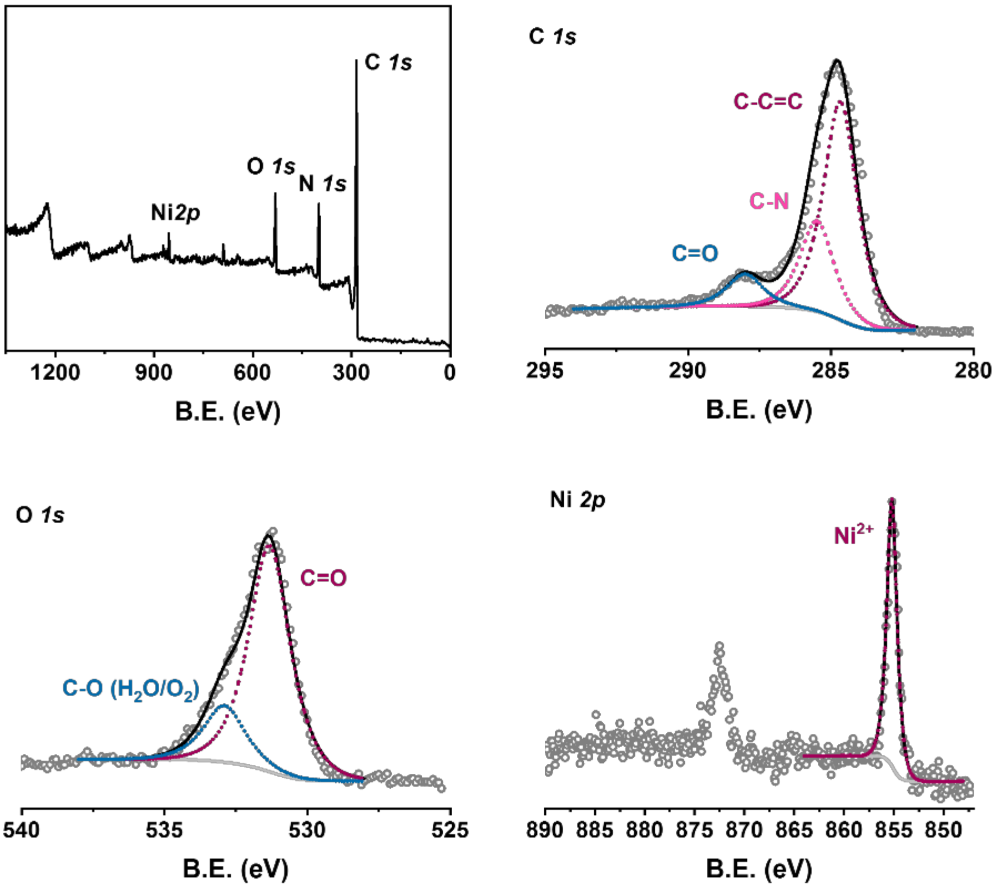

Supplementary Figure 39. XPS survey spectrum and the high-resolution C 1*s*, O 1*s*, and Ni 2*p* spectra of 2DCP-NiPc.

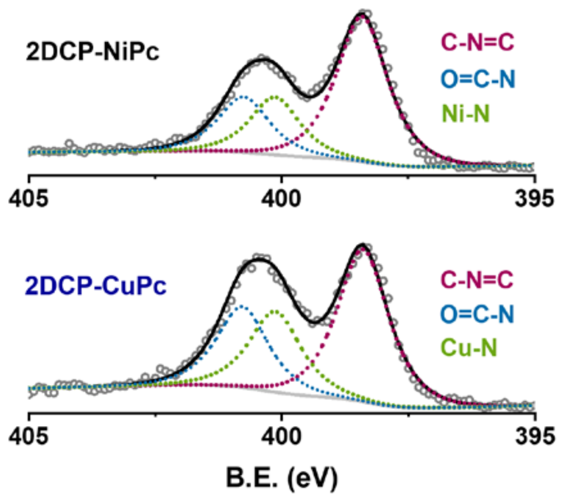

Supplementary Figure 40. XPS N 1*s* spectra of 2DCP-MPcs.

High-resolution XPS N 1*s* spectra reveal three types of N atoms at 398.4, 400.1, and 400.8 eV, which can be assigned to the imidazole (C–N=C and O=C–N)[23] and phthalocyanine (C–N=C and M–N) moieties. The calculated core-level energies for the N-atoms in the monolayer are also split into three groups: 380.3, 381.2, 382.3 eV corresponding to the C–N=C, M–N, and O=C–N, respectively (note that the calculated absolute values have no practical significance).

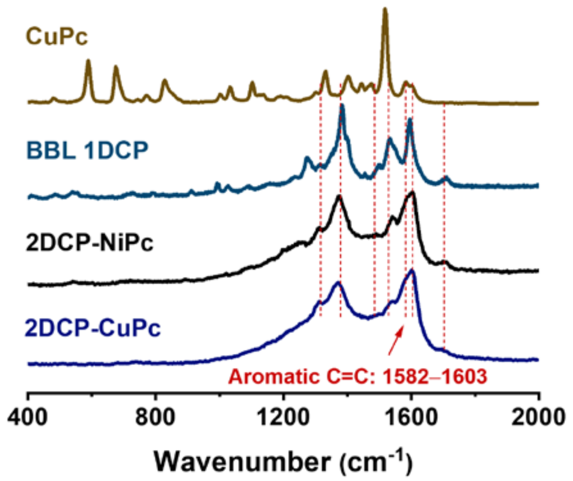

Supplementary Figure 41. Raman spectra of 2DCP-MPcs.

The Raman spectra present peaks matching well with those of the commercial copper phthalocyanine (CuPc) and BBL 1DCP.

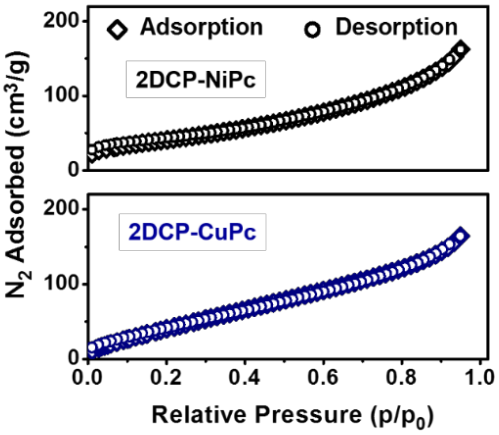

Supplementary Figure 42. Nitrogen physisorption of 2DCP-MPcs.

The crystalline **2DCP-NiPc** and **2DCP-CuPc** present Brunauer−Emmett−Teller surface areas of 138 and 191 $m^2\ g^{-1}$, respectively. The moderate surface areas are attributed to the densely packed 2DCP layers.

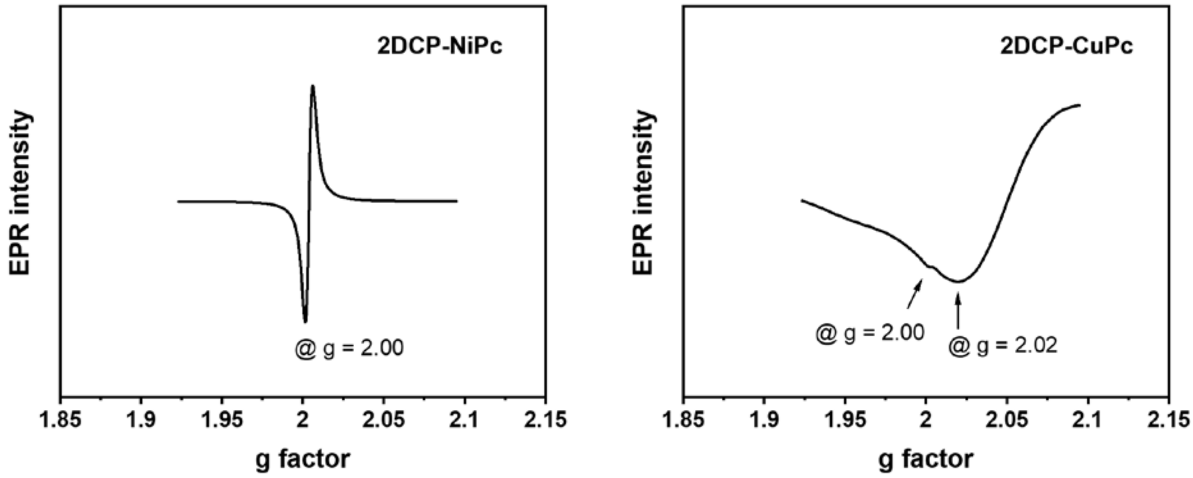

Supplementary Figure 43. Electron paramagnetic resonance (EPR) spectra of 2DCP-MPcs at room temperature.

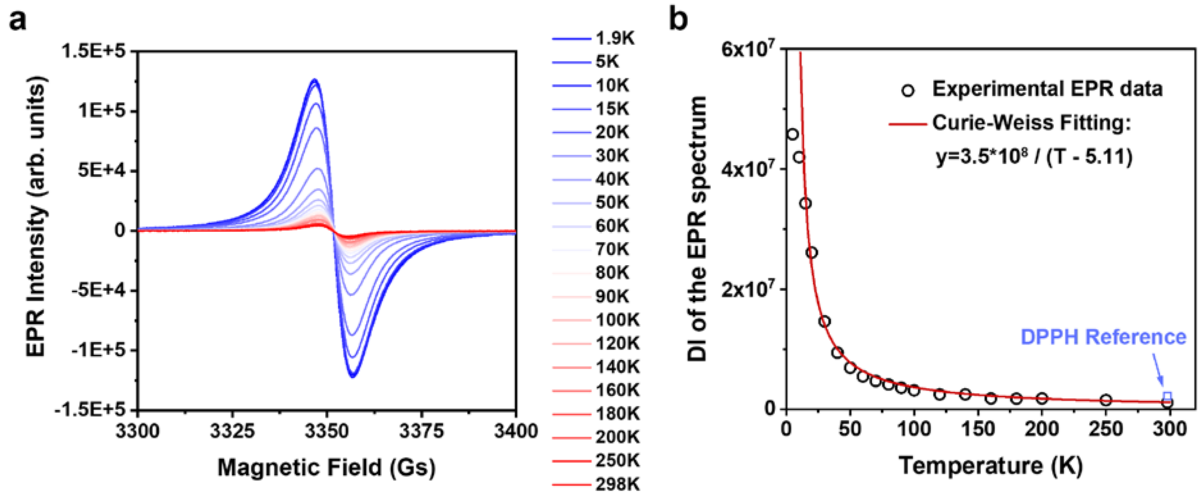

Supplementary Figure 44. Temperature-dependent EPR spectra of 2DCP-NiPc. a, EPR spectra in the temperature range of 1.9–298 K. **b,** Temperature dependence of the double-integration (DI) of the EPR spectra.

The EPR signals at g = 2.00 of both 2DCPs (Supplementary Figure 43) may be attributed to partial oxidation by air during sample treatment or induced biradicals for a partial polymer quinoid structure,[24] which has been widely observed in linear conjugated polymers and 2D conjugated covalent organic frameworks (2D *c*-COFs).[25-28] Using 2,2-diphenyl-1-picrylhydrazyl (DPPH) as a reference (Supplementary Figure 44b), which contains one spin per molecule, the spin number in **2DCP-NiPc** is measured to be 0.21 per unit cell (per phthalocyanine) at 298 K.

As is known, the double-integration (DI) of an EPR spectrum is proportional to the magnetic susceptibility, the temperature dependence of which can indicate whether a spin is localized or not: the susceptibility of localized spins is temperature-dependent while it is not for delocalized spins.[29] For **2DCP-NiPc**, the DI is calculated and plotted versus temperature in Supplementary Figure 44b. It can be seen that DI depends strongly on temperature. Moreover, the DI can be well fitted to the Curie-Weiss law which also describes temperature dependence of magnetic susceptibility of localized spins. In a word, these data indicate that the EPR signal of the sample mainly comes from localized spins, which do not delocalize prior to external excitation (e.g., optical excitation).

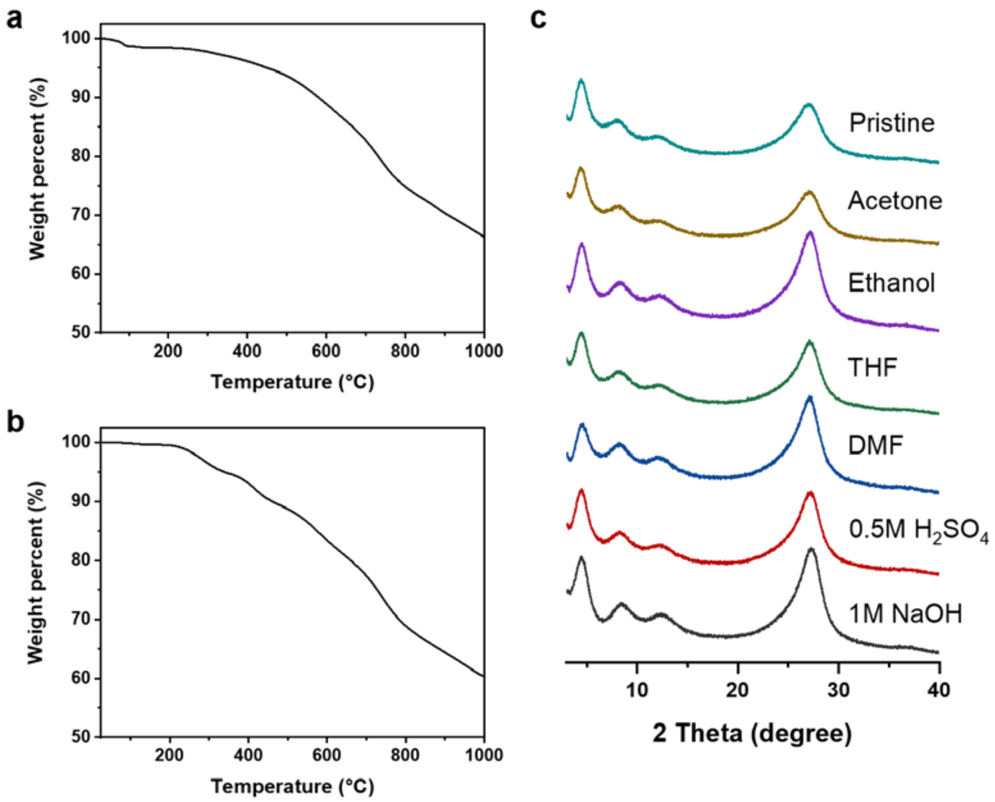

Supplementary Figure 45. Thermal and chemical stability of 2DCP-MPcs. (a,b) Thermogravimetric analysis of **2DCP-CuPc** and **2DCP-NiPc**, respectively. (c) PXRD patterns of **2DCP-CuPc** after soaking in various solvents including acetone, ethanol, tetrahydrofuran, dimethylformamide, and 0.5 M H_2SO_4/1.0 M NaOH aqueous solutions at room temperature for 24 h. The results were recorded in reflection geometry (on Panalytical) rather than in transmission geometry (on Stoe Stadi-P). The retained PXRD patterns after the treatments demonstrate high chemical stability.

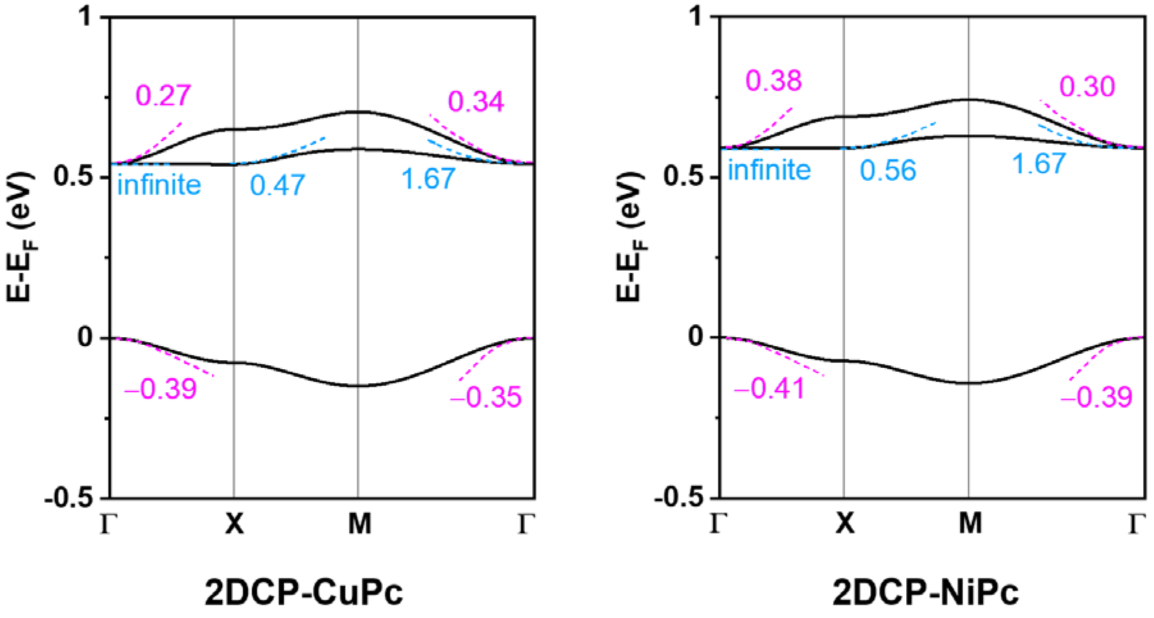

Supplementary Figure 46. Parabolic fits to the CBMs and VBM (dash lines) to estimate the effective masses of electrons and holes for monolayer 2DCP-CuPc (left) and 2DCP-NiPc (right). The figures are enlarged Figures 3a and 3b.

For the monolayer **2DCP-CuPc**, the two lowest conduction bands are nearly degenerate at Γ point. Thus, there are two types of electrons (i.e., light and heavy) at the Γ point, associated with two effective masses: 0.34 and 1.67 m_0 in the Γ→ M direction; 0.27 m_0 and infinitely high in the Γ→ X direction (see details in Supplementary Table 1). Since the CBM appears at X and Γ points, we have also evaluated the electron effective mass at X point to be 0.47 m_0 in the X→ M direction. We obtain further averaged effective masses for holes or electrons ($m^*_{h(\mathrm{avg})}$ or $m^*_{e(\mathrm{avg})}$) as 0.369 or 0.427 m_0, respectively. The electron-hole reduced effective mass (m^*, $\frac{1}{m^*} = \frac{1}{m^*_{e(\mathrm{avg})}} + \frac{1}{m^*_{h(\mathrm{avg})}}$) is inferred to be 0.198 m_0. Note that we use and present all m^*_h in this work in the absolute value.

$$m^*_{h(\mathrm{avg})} = 2[\frac{1}{m^*_{h(\Gamma\mathrm{X})}} + \frac{1}{m^*_{h(\Gamma\mathrm{M})}}]^{-1};\ m^*_{e(\mathrm{avg})} = 4[\frac{1}{m^*_{e(\mathrm{XM})}} + \frac{1}{m^*_{e(\Gamma\mathrm{M})}} + \frac{1}{m^*_{e(\Gamma\mathrm{X}+1)}} + \frac{1}{m^*_{e(\Gamma\mathrm{M}+1)}}]^{-1}$$

Monolayer **2DCP-NiPc** shows almost identical energy bands as monolayer **2DCP-CuPc** near the Fermi level. Similarly, $m^*_{h(\mathrm{avg})}$ and $m^*_{e(\mathrm{avg})}$ for **2DCP-NiPc** are calculated to be 0.400 and 0.479 m_0, respectively. This leads to a slightly larger m^* than that of monolayer

2DCP-CuPc (m^*: 0.218 vs. 0.198 m_0). Given that AA-slipped stacked **2DCP-NiPc** presents a slightly smaller m^* than that of AA-slipped stacked **2DCP-CuPc** (0.137 vs. 0.172 m_0, Supplementary Table 2), we anticipate stronger intermolecular *d*(metal)-π and/or *d*(metal)-*d*(metal) interactions for the former. This is further confirmed by the metal *d* orbital-projected band structures shown in Supplementary Fig. 48, where the Ni *d* orbital contributes more to the band edges near the Fermi level than the Cu *d* orbital, despite their similar PDOS shown in Figure 3.

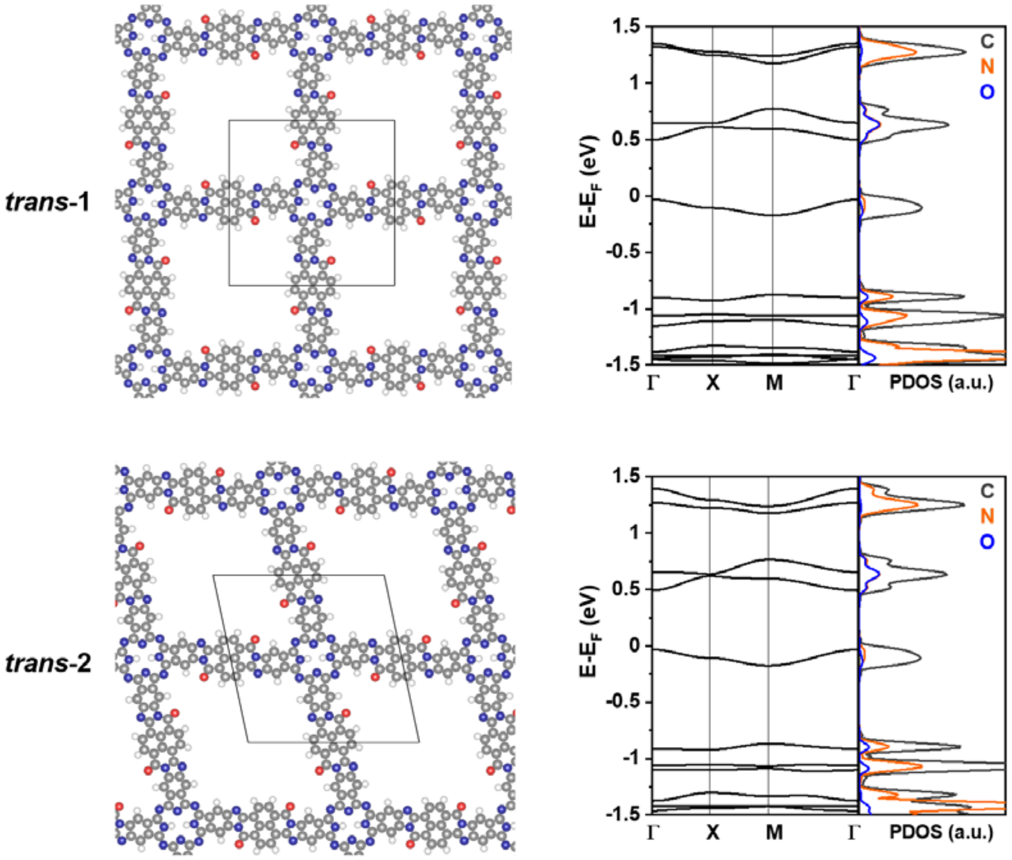

Supplementary Figure 47. Electronic band structures of monolayers of the *trans*-isomeric 2DCP-MPc (metal-free *trans*-1 and *trans*-2).

As discussed on Supplementary Figure 28, only trace ***trans*-2** and ***cis*-1** can potentially form at the edges of ***trans*-1** crystal domain. To explore the electronic effects of *trans*- and *cis*-isomers on the BBL-ladder-type backbone and to simplify the calculations (due to the large 2×2 replicated cell of ***trans*-1** and equal cell of ***cis*-1** shown in Supplementary Figure 28), we first compared the electronic structures and HOMO/LUMO energy levels of the model compounds (**M6** and **M6-iso1**/**M6-iso2**) of ***trans*-1** and ***cis*-1** in Supplementary Figures 6 and 7. They only show negligible difference, implying that the electronic properties (e.g., bandgap, effective mass) of ***trans*-1** and ***cis*-1** are similar

The electronic band structures of the monolayers of metal-free ***trans*-1** and ***trans*-2** were further compared. As shown in Supplementary Figure 47, they present almost identical band structures near the Fermi level, and thus identical effective masses for electrons and holes.

Taking all the results into consideration, the isomeric structures (***trans*-1**, ***trans*-2** and ***cis*-1**) do not influence the electronic structures of **2DCP-MPc**.

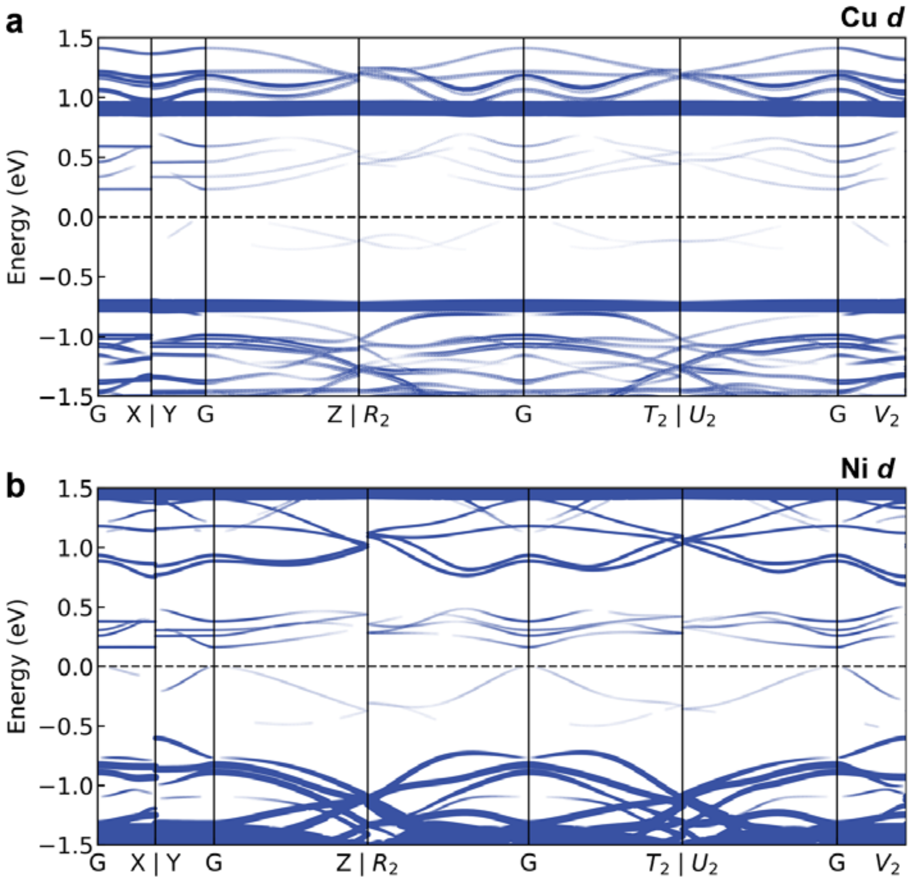

Supplementary Figure 48. Metal *d* orbital-projected band structures for the AA-slipped stacked 2DCP-CuPc (a) and 2DCP-NiPc (b).

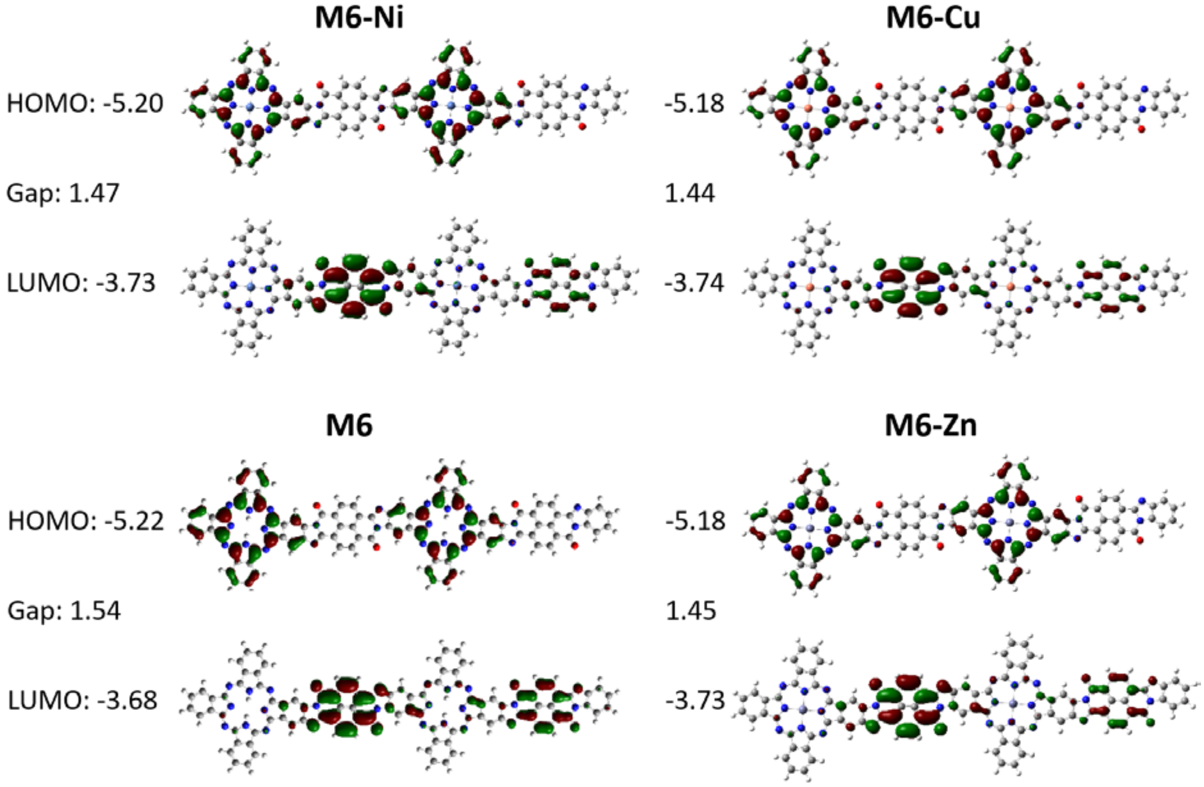

Supplementary Figure 49. Molecular electronic structure of the metal-embedded M6.

Clearly, Ni, Cu, or Zn does not hybridize with the phthalocyanine core, but the incorporation of metal centers into **M6** slightly reduces the HOMO–LUMO gap (by 0.07–0.10 eV). The comparison of the HOMO/LUMO of **M6-Ni** and **M6-Cu** suggests a minimal influence of changing the metal center from Ni to Cu on the electronic structure.

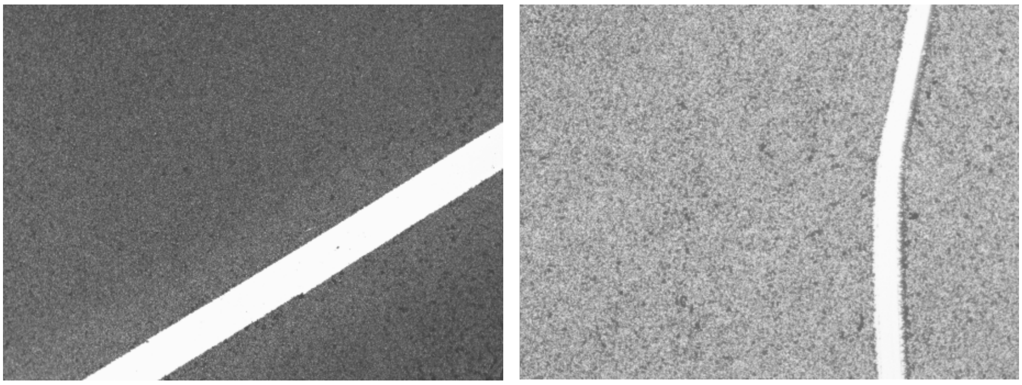

Supplementary Figure 50. Optical microscopy images of the as-synthesized 2DCP-NiPc (let) and 2DCP-CuPc (right) film on fused silica substrate.

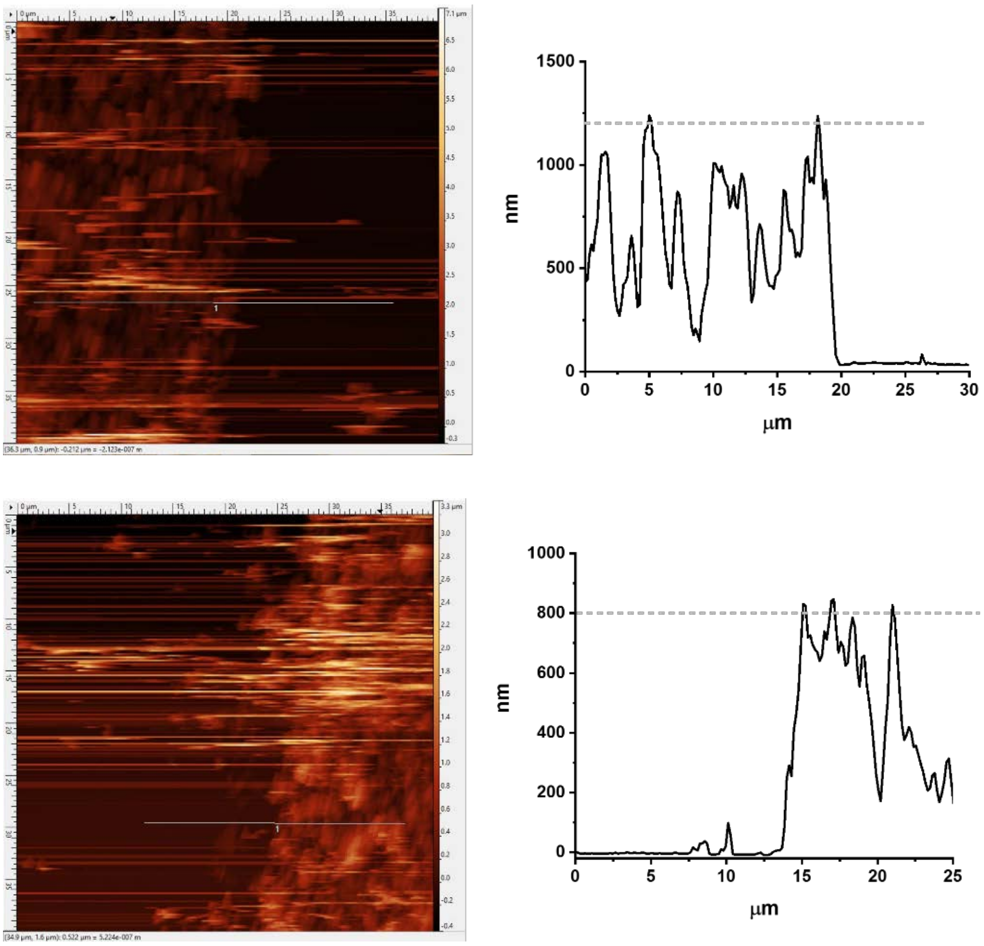

Supplementary Figure 51. Atomic force microscopy (AFM) images of 2DCP-NiPc (top) or 2DCP-CuPc (down) film on fused silica substrate. The thickness is ca. 1200 and 800 nm for **2DCP-NiPc** and **2DCP-CuPc**, respectively.

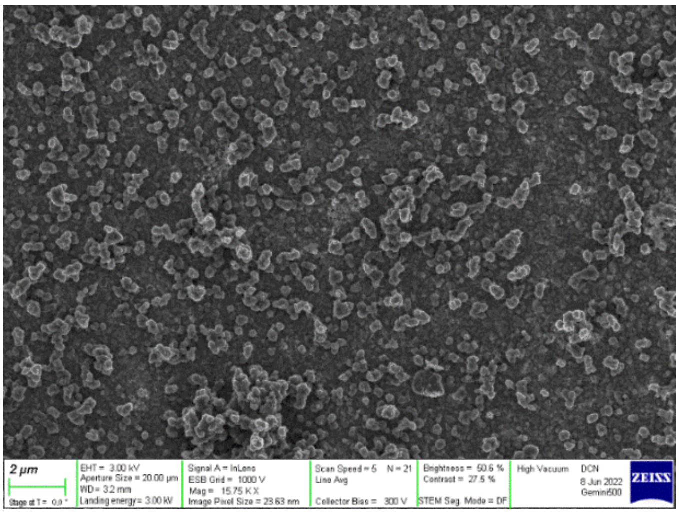

Supplementary Figure 52. SEM image of 2DCP-NiPc film on fluorine doped tin oxide (FTO)-coated glass substrate. The 2DCP films on the insulating fused silica substrates face charging issue during the SEM measurements.

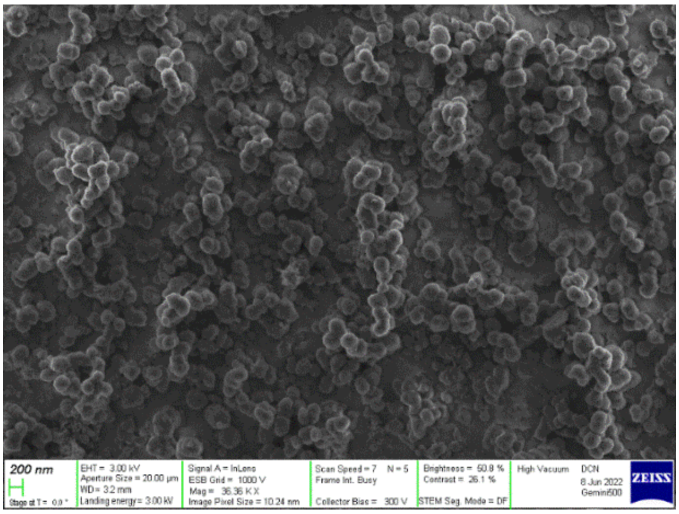

Supplementary Figure 53. SEM image of 2DCP-CuPc film on FTO-coated glass substrate.

The **2DCP-NiPc** film is continuous, which also contains many big **2DCP-NiPc** particles on top of the bottom film (Supplementary Figure 52). However, many pinholes are observed in the **2DCP-CuPc** film, which indicates higher defect density than that in the **2DCP-NiPc**

film.

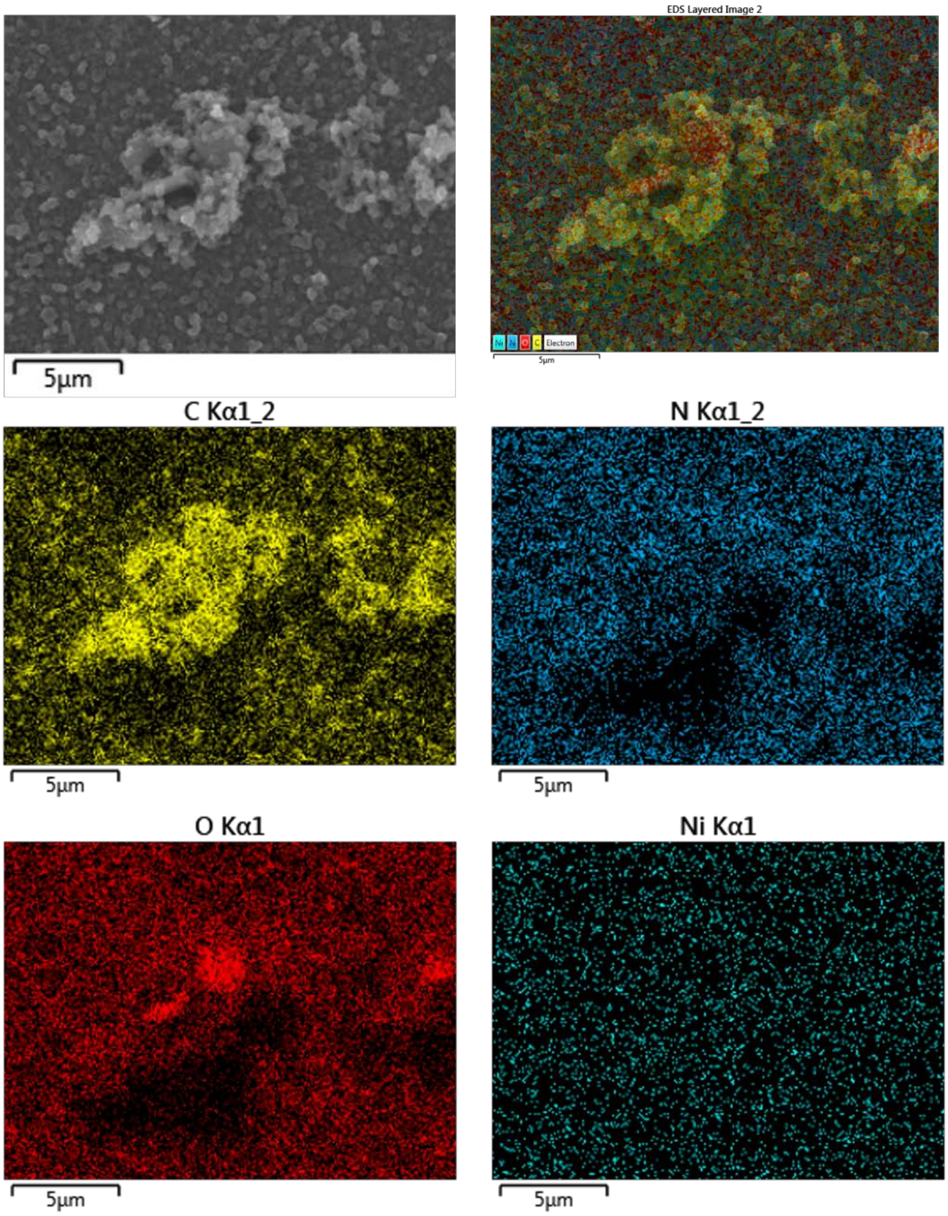

Supplementary Figure 54. EDX spectroscopy of the elemental mapping images of 2DCP-NiPc film on FTO-coated glass substrate.

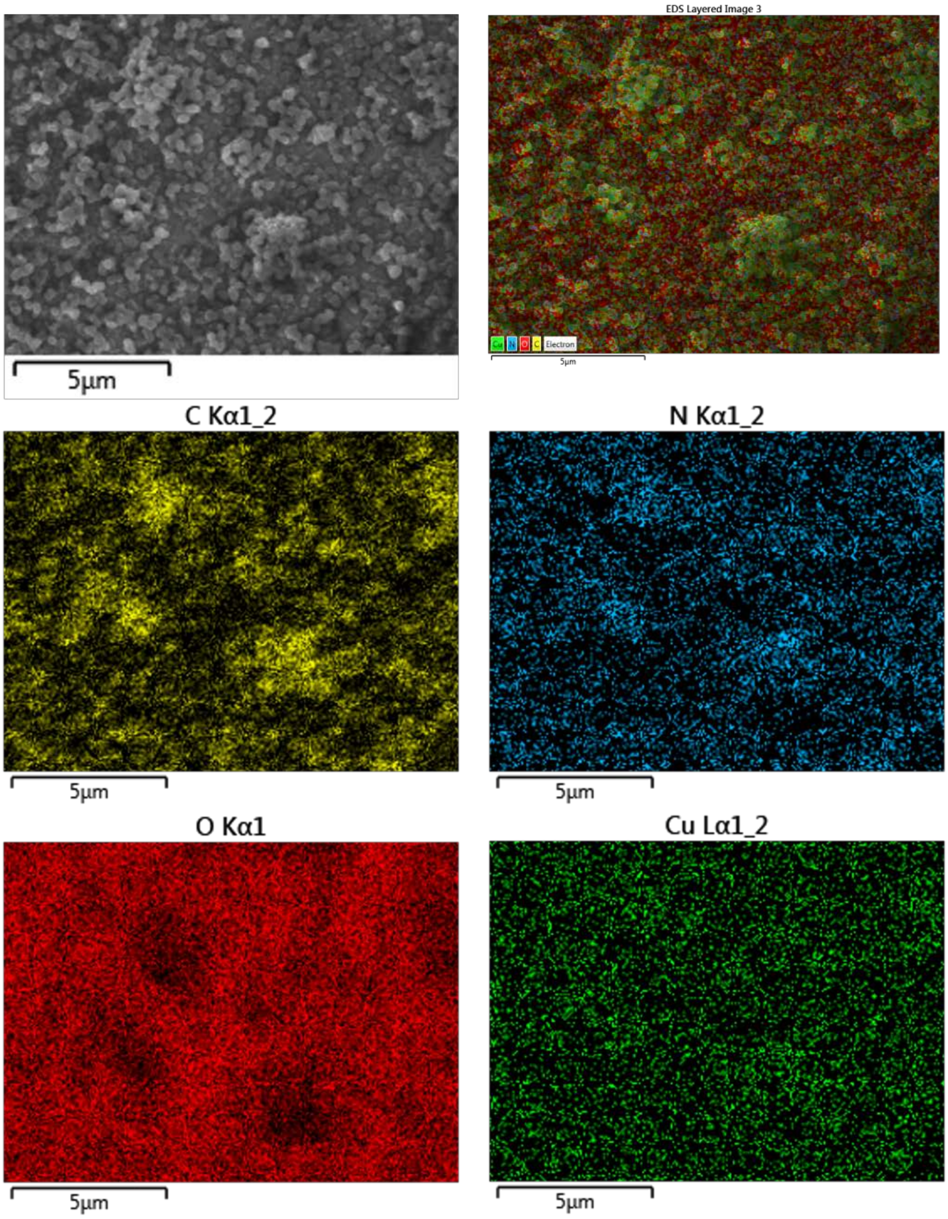

Supplementary Figure 55. EDX spectroscopy of the elemental mapping images of 2DCP-CuPc film on FTO-coated glass substrate.

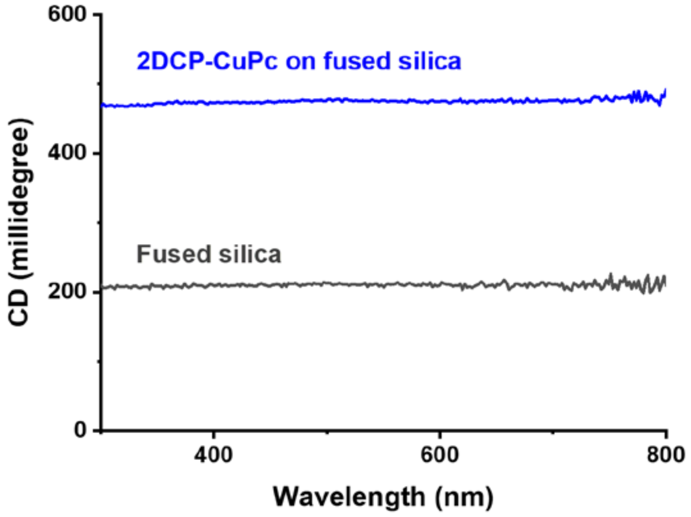

Supplementary Figure 56. Circular dichroism spectra of blank fused silica substrate and 2DCP-CuPc film on fused silica. 2DCP-CuPc film does not show chiral behavior in the UV-visible range.

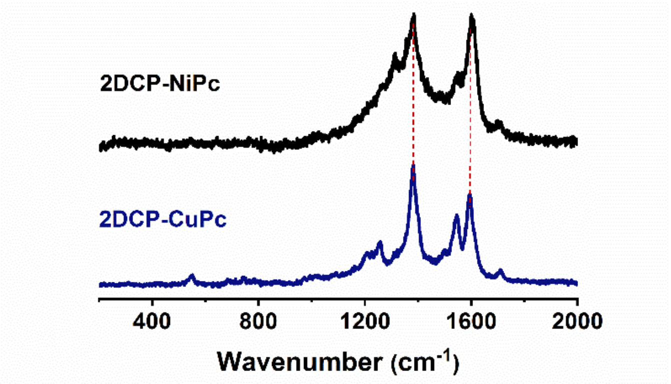

Supplementary Figure 57. Raman spectra of 2DCP-MPc film on fused silica substrate. The film samples display similar peaks to those of the **2DCP-MPcs** powders.

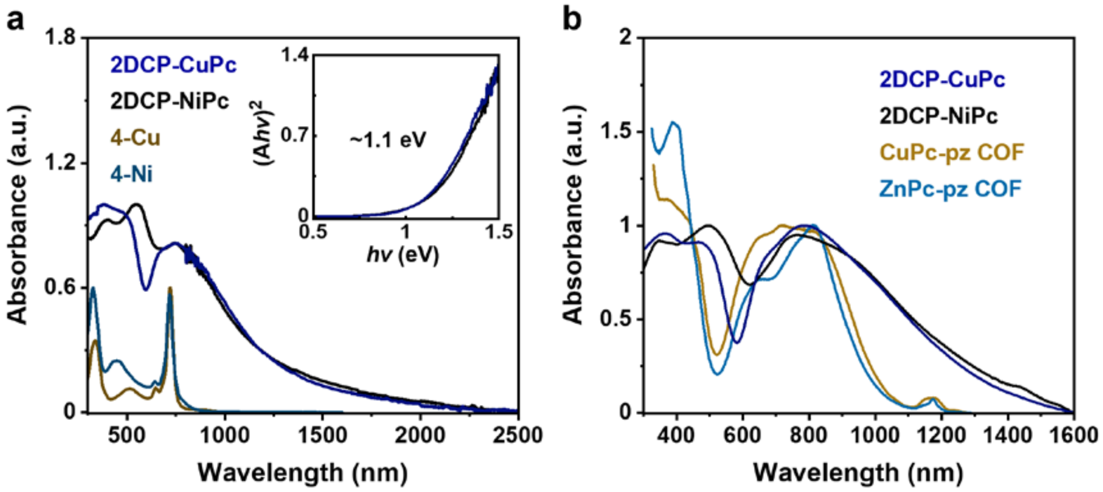

Supplementary Figure 58. UV-visible-near IR absorption of 2DCP-MPc film on fused silica (a) and the dispersions of 2DCP-MPc powders in DMSO (b) measured without eliminating scattering. The absorption spectra of the dispersions of MPc-pz COF in DMSO[17] without eliminating scattering are shown as references.

Clearly, scattering largely influences the determination of optical bandgap. For example, the spectra measured without eliminating scattering (Supplementary Figure 58a) show a bandgap deviation of ~0.2 eV (smaller), compared to the spectra shown in Fig. 4a. In addition, **2DCP-MPcs** exhibit optical bandgaps smaller than (or at least equal to) the previously reported ladder-type MPc-pz COF[17]. So the optical bandgaps of MPc-pz COFs are set as 1.4 eV in Fig. 5b.

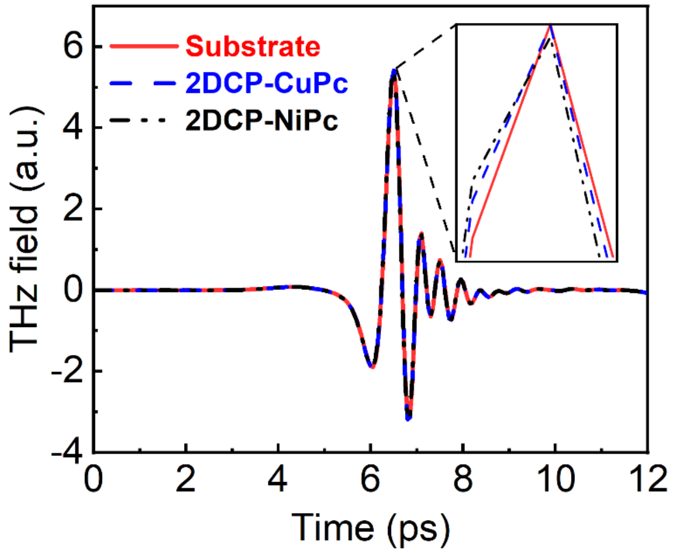

Supplementary Figure 59. Time-domain waveforms of the transmitted THz pulses through the blank fused silica substrate (red solid line), 2DCP-CuPc (blue dash line), and 2DCP-NiPc (black dash-dot line).

We observed almost no THz absorption in the as-prepared 2DCPs in the absence of optical excitation. This confirms that our samples have negligible background charge carrier density under ambient conditions, consistent with their semiconducting and undoped nature.

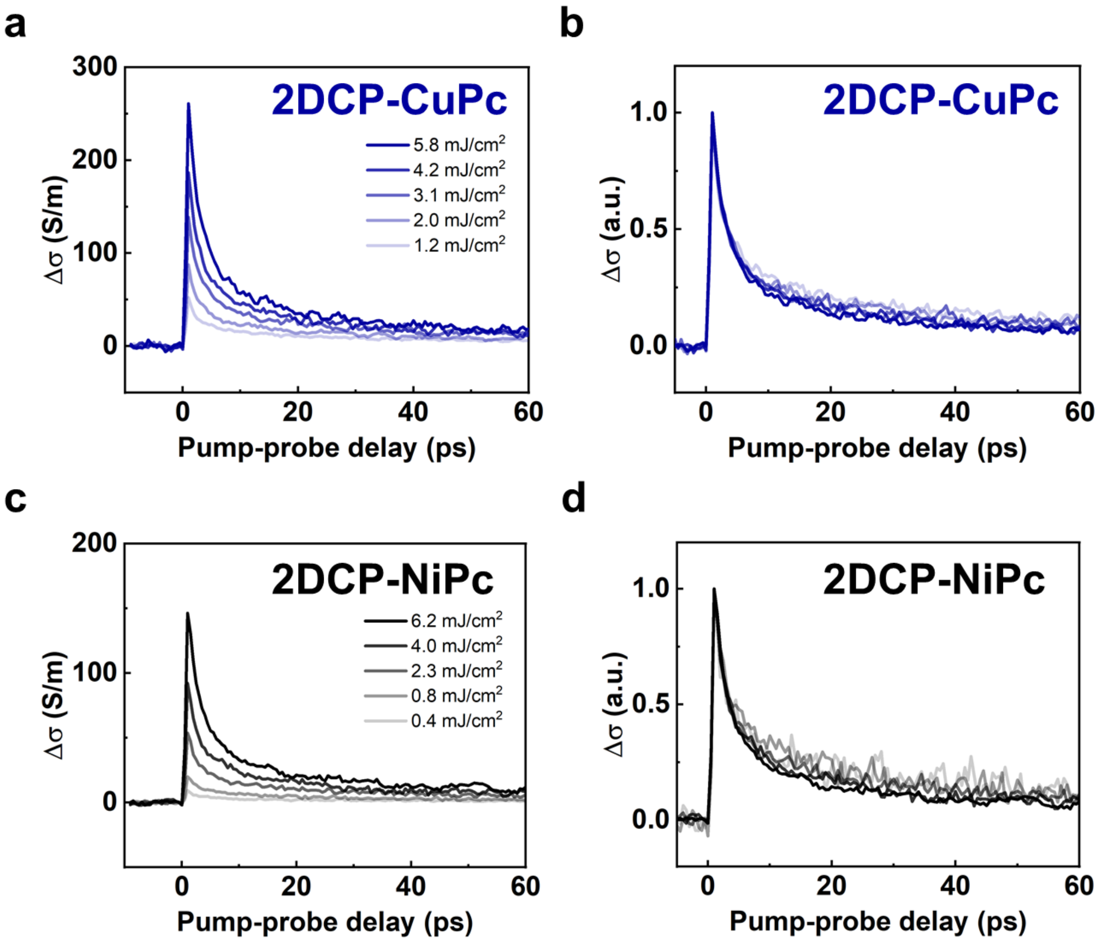

Supplementary Figure 60. Fluence-dependent THz photoconductivity dynamics of 2DCP-MPcs. a,c, Fluence-dependent THz photoconductivity of **2DCP-CuPc** and **2DCP-NiPc** films, respectively. **b,d,** Normalized fluence-dependent THz photoconductivity of **2DCP-CuPc** and **2DCP-NiPc** films, respectively.

To provide more insights, we have performed fluence-dependent THz photoconductivity measurements. We found that, in our available pump fluence range, the photoconductivity of **2DCP-MPcs** (Supplementary Figure 60a,c) increases with the pump fluence, while the normalized dynamics overlap within the experimental error (Supplementary Figure 60b,d). This result indicates that the charge carrier lifetime, or equivalently the decay rate, does not depend on the pump fluence in the pump-fluence range used in the study. Based on the data, we can safely conclude that the monomolecular recombination e.g., trap-assisted recombination, dominates the charge carrier decay. Given the extremely high charge carrier mobility, we exclude geminate charge recombination as the main recombination channel.

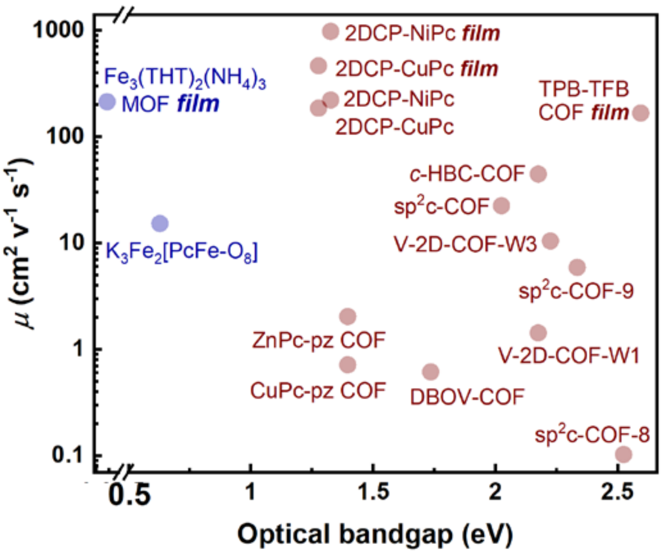

Supplementary Figure 61. Comparison of charge carrier mobilities and optical bandgaps of 2DCP-MPcs with reported COFs (light-wine spots) and MOFs (light-blue spots) measured by THz spectroscopy at ambient temperature. Most polymers were synthesized as powder samples. The film samples are labeled ***film***. Some bandgaps are estimated from the reported optical absorption spectra; note that light scattering from the samples often impedes an accurate bandgap determination.

As shown in Figure 1 and Supplementary Figure 9 by simulations, compared with the classical linkages (imine, vinylene) for 2D *c*-COFs, the BBL-ladder-type linkage confers a narrow bandgap and significantly enhanced intramolecular interaction to the resultant polymer backbone. These predictions have panned out experimentally: **2DCP-MPcs** show the lowest optical bandgaps (~1.3 eV), the smallest effective charge carrier masses ($m*$, in the range of 0.137–0.172 m_0) and the highest charge carrier mobilities (μ, up to 971 $cm^2\ V^{-1}\ s^{-1}$ at room temperature) among the reported 2DCPs and COFs (see details in Supplementary Table 4).

Besides $m*$, the charge carrier scattering time (τ) plays an important role in determining μ $\left(= \frac{e\tau}{m^*}(1+c)\right.$, following the Drude-Smith model), where c describes the backscattering probability (see detailed discussion related to Supplementary Figure 62). Extensive π-delocalization and high crystallinity (implying low-level density of structural defects/boundaries) are expected to positively affect τ. Taking imine-linked 2D c-COFs as

references, **2DCP-MPcs** and state-of-the-art (in terms of conjugation, vinylene-linked[30,31] or ladder-type-pyrazine-linked[17,32]) 2D *c*-COFs present similar levels of crystallinity (that is yet far below that of the highly crystalline imine-linked 2D *c*-COFs). The strongly π-delocalized **2DCP-MPcs** exhibit τ (of ca. 70 fs) exceeding that of the latter in the range of ca. 30–50 fs (Supplementary Table 4). Increasing the crystallinity of **2DCP-MPcs** to the level of imine-linked 2D *c*-COFs might further elevate the currently obtained τ and thus μ.

The charge transport properties (τ, m^*, c, and μ) of representative 2DCPs are listed below (see more details in Supplementary Table 4).

2DCP-NiPc (BBL): $m^* = 0.137\ m_0$, $\tau = 76 \pm 3$ fs, $c = 0$, $\mu = 971$ cm^2 V^{-1} s^{-1}

sp^{2}c-COF (vinylene)[30]: $m^* = 0.2\ m_0$, $\tau = 41 \pm 5$ fs, $c = -0.94$, $\mu = 22.1$ cm^2 V^{-1} s^{-1}

ZnPc-pz (pyrazine)[17]: $m^* = 2.3\ m_0$ (holes), $\tau = 30 \pm 4$ fs, $c = -0.91$, $\mu = 2.0$ cm^2 V^{-1} s^{-1}

TPB−TFB COF (imine)[33]: $m^* = 0.78\ m_0$, $\tau = \sim 72$ fs, $c = 0$, $\mu = 165$ cm^2 V^{-1} s^{-1}

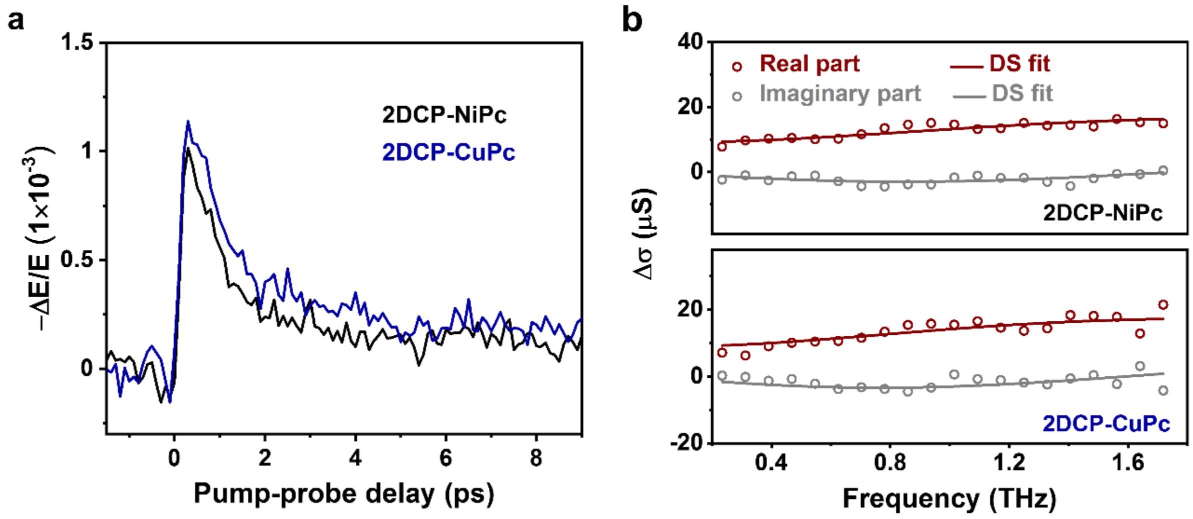

Supplementary Figure 62. THz spectroscopy of 2DCP-MPc powder samples. a, THz photoconductivity dynamics. **b,** Frequency-resolved complex photoconductivity. The solid lines correspond to the Drude-Smith (DS) fits describing the real and imaginary components of the complex THz photoconductivity, respectively.

We investigated the charge transport properties of **2DCP-MPc** in power form: a sample geometry that has been widely used to investigate 2D *c*-COFs by THz spectroscopy.[17,30,34] The **2DCP-MPc** powder samples were first sandwiched between two fused silica substrates to form ~10s of μm thick thin films. By measuring the transmitted THz traces with and without photoexcitation in the time domain (E_{pump}(t) and E_0(t)) and further converting them into the frequency domain by Fourier transform ($E_{pump}(\omega)$ and $E_0(\omega)$), we can obtain their complex photoconductivity ($\Delta\sigma(\omega)$) following the thin-film approximation:

$$\Delta\sigma(\omega) = -\frac{n_1 + n_2}{Z_0 l} \cdot \frac{(E_{pump}(\omega) - E_0(\omega))}{E_0(\omega)}$$

where Z_0 = 377 Ω is the impedance of free space, n_1 and n_2 are the refractive indices of the media before and after the sample, and l is the excitation thickness. We use $l = 1$ to obtain the sheet complex sheet photoconductivity ($\Delta\sigma_s(\omega)$).

$\Delta\sigma_s(\omega)$ of **2DCP-MPc** powder samples was fitted by the DS model, which provides a phenomenological description of confined charge transport in the material:

$$\sigma_{DS}(\omega) = \frac{\omega_p^2 \varepsilon_0 \tau_{DS}}{1 - i\omega\tau_{DS}} \cdot (1 + \frac{c}{1 - i\omega\tau_{DS}})$$

where c is the backscattering probability and τ_{DS} is the DS relaxation time. Here we use the DS relaxation time to approximate the Drude scattering time, which is in reality a function of both the DS relaxation time and diffusion time[35].

Photoconductivity measurements yield spectral responses that differ from the Drude behavior (Supplementary Figure 62b). The data can be well fitted by the DS model shown in solid lines. The DS model phenomenologically describes transport of free charge carriers in nanomaterials where the backscattering of charge carriers occurs e.g. at grain boundaries. In the model, a parameter c ranging from 0 (isotropic scattering) to -1 (100% backscattering) evaluates the backscattering probability. The best DS description of the data provide effective scattering times of 69 ± 7 and 61 ± 4 fs as well as c parameters of -0.74 ± 0.02 and -0.72 ± 0.01 for **2DCP-CuPc** and **2DCP-NiPc**, respectively. Using $m*$ from DFT calculation and τ as well as c from the DS fitting, we can estimate the charge carrier mobility following: $\mu = \frac{e\tau}{m^*}(1 + c)$ in the dc limit. This also yields very high mobilities of 183 ± 19 and 219 ± 14 $cm^2\ V^{-1}\ s^{-1}$ for **2DCP-CuPc** and **2DCP-NiPc**, respectively.

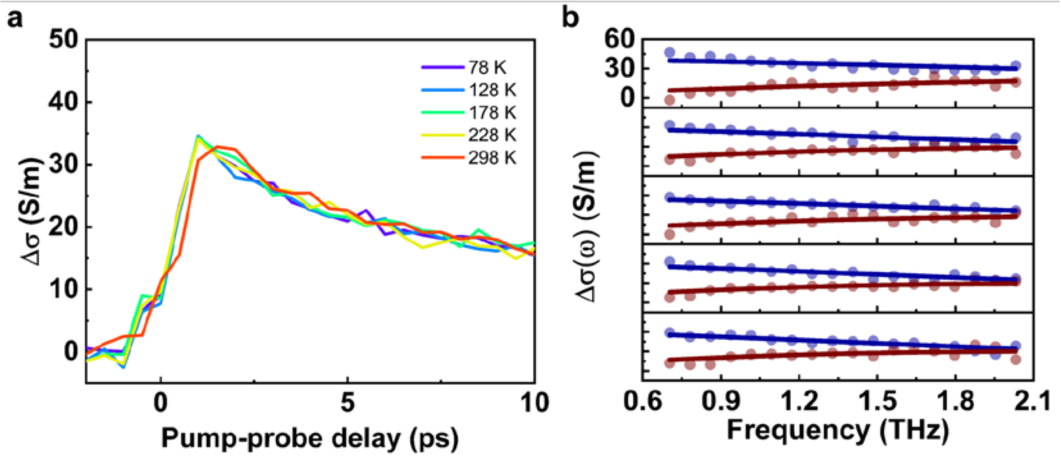

Supplementary Figure 63. Temperature-dependent THz photoconductivity of 2DCP-CuPc thin film. **a**, Temperature-dependent THz photoconductivity dynamics of **2DCP-CuPc**. **b,** Temperature-dependent frequency-resolved complex THz photoconductivity measured at the ~0.5 ps after the maximum photoconductivity of **2DCP-CuPc**, from top to bottom: 298 K, 228 K, 178 K, 128 K, 78 K. The solid lines correspond to the Drude fits describing the real and imaginary components of the complex THz photoconductivity.

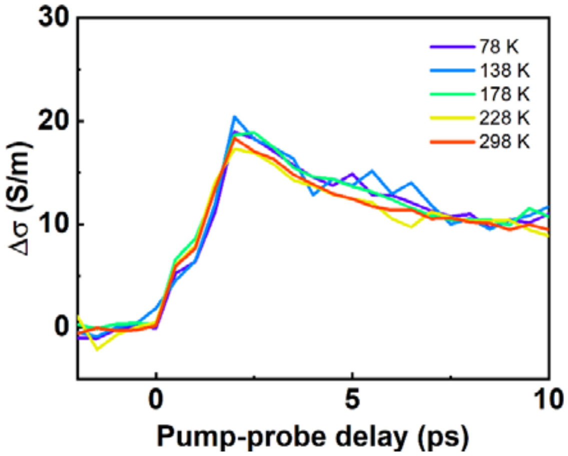

Supplementary Figure 64. Temperature-dependent THz photoconductivity dynamics of 2DCP-NiPc thin film.

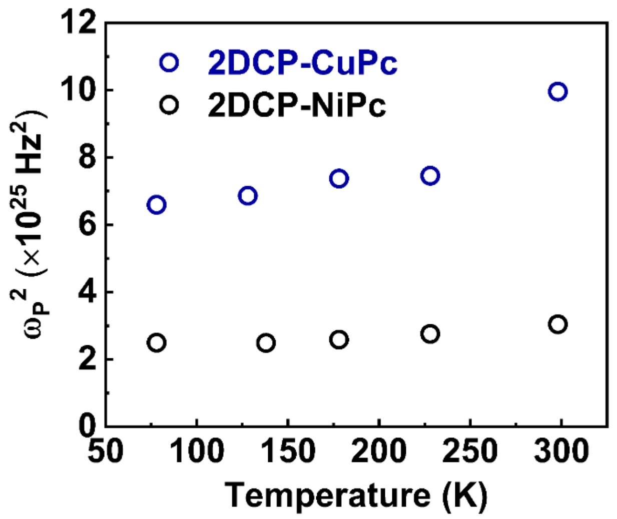

Supplementary Figure 65. **Temperature-dependent ω_p^2 of 2DCP-CuPc and 2DCP-NiPc thin films.**

Section D. Supplementary Tables

Supplementary Table 1. Effective masses of 2DCP-MPc monolayers obtained from band structure calculations. Different fitting methods were used to obtain the effective masses.

**2DCP-MPc**	**Hole effective mass (m_0)**[a]		**Electron effective mass (m_0)**		
	Γ−X	Γ−M	Γ−X	X−M	Γ−M
**Calculated by SUMO Python toolkit**[16]**:** *Parabolic fitting (inaccurate m_e*)*					
**Ni**	0.370	-	10.00 (1st CB)	-	-
**Cu**	0.351	-	10.00 (1st CB)	-	-
**Calculated by manual fitting:** *Parabolic fitting*					
**Cu**	0.39	0.35	Infinite (1st CB)	0.47 (1st CB)	1.67 (1st CB)
			0.27 (2nd CB)	-	0.34 (2nd CB)
**Ni**	0.41	0.39	Infinite (1st CB)	0.56 (1st CB)	1.67 (1st CB)
			0.38 (2nd CB)	-	0.30 (2nd CB)

[a]Note that m_h^* is mathematically negative. However, to avoid misunderstanding in the calculation, we use and present all m_h^* in this work in the absolute value.

1st CB = the lowest conduction band; 2nd CB = the 2nd lowest conduction band

Supplementary Table 2. Effective masses of AA-slipped stacked 2DCP-MPcs obtained from band structure calculations by SUMO Python toolkit[16]. All m_h^* take the absolute value for calculation.

2DCP-MPc	Effective masses (m_0) at different K-paths						
	**1**	**2**	**3**	**4**	**5**	**6**	**7**
	**Γ−Y**	**Γ−Z**	**Γ−R_2**	**Γ−T_2**	**Γ−U_2**	**Γ−V_2**	**Γ−X**
**Ni (*h*)**[a]	0.125	0.803	0.264	0.278	0.530	0.126	0.362
**Ni (*e*)**	0.139	1.664	0.454	0.368	1.933	0.180	infinite
**Cu (*h*)**[a]	0.129	2.338	0.496	0.550	1.104	0.147	0.354
**Cu (*e*)**	0.145	2.275	0.766	0.59	2.669	0.207	14.634

[a]Note that m_h^* is mathematically negative. However, to avoid misunderstanding in the calculation, we use and present all m_h^* in this work in the absolute value.

$$m_{h(\mathrm{avg})}^* = 7[\frac{1}{m_1^*}+\frac{1}{m_2^*}+\frac{1}{m_3^*}+\frac{1}{m_4^*}+\frac{1}{m_5^*}+\frac{1}{m_6^*}+\frac{1}{m_7^*}]^{-1}$$

$$m_{e(\mathrm{avg})}^* = 6[\frac{1}{m_1^*}+\frac{1}{m_2^*}+\frac{1}{m_3^*}+\frac{1}{m_4^*}+\frac{1}{m_5^*}+\frac{1}{m_6^*}]^{-1}$$

$$\frac{1}{m^*} = \frac{1}{m_{e(\mathrm{avg})}^*}+\frac{1}{m_{h(\mathrm{avg})}^*}$$

2DCP-NiPc: averaged hole effective mass $m_{h(\mathrm{avg})}^* = 0.240\, m_0$

averaged electron effective mass $m_{e(\mathrm{avg})}^* = 0.319\, m_0$

electron-hole reduced effective mass $m^* = 0.137\, m_0$

2DCP-CuPc: averaged hole effective mass $m_{h(\mathrm{avg})}^* = 0.310\, m_0$

averaged electron effective mass $m_{e(\mathrm{avg})}^* = 0.386\, m_0$

electron-hole reduced effective mass $m^* = 0.172\, m_0$

Supplementary Table 3. Bi-exponential fits and carrier lifetimes of 2DCP-MPc films.

Materials	A_1 (unitless)	t_1 (ps)	A_2 (unitless)	t_2 (ps)
**2DCP-CuPc**	14.6	4.5	17.8	34.3
**2DCP-NiPc**	8.9	4.5	9.0	40.5

The carrier lifetimes (t) of **2DCP-MPc** films are calculated by $t = \frac{A_1 t_1 + A_2 t_2}{A_1 + A_2}$.

We describe the photoconductivity decay of **2DCP-MPc** films by a bi-exponential function as follows:

$$y = A_1 e^{-\frac{x}{t_1}} + A_2 e^{-\frac{x}{t_2}}$$

Supplementary Table 4. **A comparison of charge carrier mobilities of 2DCP-MPcs (films and powders) with 1DCPs (including graphene nanoribbons) and other organic 2D framework materials (e.g., COFs, MOFs) measured by THz spectroscopy at ambient temperature.**

In our work, we estimate the charge carrier mobility at the dc limit. For Drude-Smith-type charge transport, this reads: $\mu = \frac{e\tau}{m^*}(1+c)$. For Drude-type transport, this reduces to $= \frac{e\tau}{m^*}$ (as c = 0). We provide a comprehensive summary of μ values together with τ and c as well as the inferred m^* from calculation. The stacking modes of the layer-stacked polymers and the theoretical calculation methods for effective mass calculation are also offered, i.e., AA, AA-s (AA-slipped), ABC. Note that most polymers were synthesized as powder samples. Only the film samples are labeled ***film***.

As non-contact THz spectroscopy was employed to reveal the intrinsic charge carrier mobilities of the developed **2DCP-MPcs**, we have summarized the information of 1DCPs, 2DCPs, COFs and MOFs characterized by the same technique for a fair comparison, and also offered the data of COFs obtained using another well-known non-contact technique, i.e., time-resolved microwave conductivity (TRMC). The mobilities obtained by field-effect transistor and Hall effect measurements are not considered in this table.

**Sample**	**Theory method**[a]	**Applied m^* (m_0)**	**τ (fs)**	**c**	**μ ($cm^2\ V^{-1}\ s^{-1}$)**
**2DCP-NiPc *film***	DFT (*AA-s*)	0.137[b]	76 ± 3	0 (Drude)	**971 ± 44 (*r.t.*)**
			108 ± 7	0 (Drude)	1386 ± 96 (*78K*)
**2DCP-CuPc *film***		0.172[b]	45 ± 3	0 (Drude)	**460 ± 31 (*r.t.*)**
			69 ± 4	0 (Drude)	706 ± 37 (*78K*)
**2DCP-NiPc**		0.137[b]	61 ± 4	−0.72 ± 0.01	219 ± 14 (*r.t.*)
**2DCP-CuPc**		0.172[b]	69 ± 7	−0.74 ± 0.02	183 ± 19 (*r.t.*)
**2D conjugated COFs (*conjugated polymers*)**					
ZnPc-pz[17]	DFT (*AA-s*)	2.3 (*hole*)	30 ± 4	−0.91 ± 0.02	~2.0

CuPc-pz		2.3 (*hole*)	30 ± 4	−0.97 ± 0.02	~0.7
sp^2c-COF[30]	DFTB (*AA*)	0.2*[b]*	41 ± 5	−0.94 ± 0.02	22.1 ± 2.7
sp^2c-COF-6		~0.9*[b,c]*	~35*[c]*	N/A	2.3 ± 0.5
sp^2c-COF-8		~0.9*[b,c]*	~10*[c]*	N/A	<0.1
sp^2c-COF-9		~0.9*[b,c]*	~50*[c]*	N/A	5.8 ± 0.9
c-HBC-COF[34]	DFTB (*AA*)	0.21*[b]*	87 ± 5	N/A	44
DBOV-COF[36]	DFTB (*ABC*)	N/A	36 ± 6	−0.85 ± 0.02	0.6 ± 0.1
V-2D-COF-W1[31]	DFT (*AA-s*)	0.63*[b]*	49 ± 8	N/A	~1.4
V-2D-COF-W3		N/A	37 ± 6	N/A	~10.3
V-2D-COF-W4		N/A	56 ± 8	N/A	~0.6
TPB−TFB COF[33] ***film***	DFTB (*AA*)	0.78*[b]*	~72	0 (Drude)	165 ± 10
**Doped 2D conjugated COFs (*conjugated polymers*)**					
ZnPc-pz-I_2[37]	DFT (*AA-s*)	2.1 (*hole*)	66 ± 3	−0.98 ± 0.02	6.3
I_2-sp^2c-COF[30]	N/A	N/A	~85*[c]*	N/A	51.1 ± 3.1
I_2-sp^2c-COF-6	N/A	N/A	~70*[c]*	N/A	5.7 ± 0.3
I_2-V-2D-COF-W1[31]	N/A	N/A	110 ± 12	N/A	3.1
I_2-V-2D-COF-W4	N/A	N/A	104 ± 14	N/A	1.2
**2D non-conjugated COFs (*polymers*)**					
CuPc-MIDA-COF[38]	DFTB (*AA*)	~0.6*[b,c]*	23 ± 13	N/A	13.3
HHTP-MIDA-COF		~1.2*[b,c]*	16 ± 12	N/A	3.4
**2D conjugated MOFs (*conjugated coordination polymers*)**					
$K_3Fe_2[PcFe\text{-}O_8]$[39]	DFT (*AA-s*)	~1.9*[d]*	53 ± 8	−0.69 ± 0.02	*15 ± 2[e]*
$Fe_3(THT)_2(NH_4)_3$[40] ***film***	DFT (*AA-s*)	0.88*[d]*	104 ± 5	0 (Drude)	211 ± 7
**1DCPs including graphene nanoribbons**					
FBDOPV-2T[41]			110 ± 7	−0.997	N/A
FBDOPV-2F2T			119 ± 4	−0.996	N/A
FBDOPV-4F2T			71 ± 4	−0.997	N/A

FGNR[42]	28 ± 1	−0.99	*104 ± 3*[e]
cGNR[43]	57 ± 3	−1	*617 ± 32*[e]
cMGNR[44]	36 ± 2	−0.97	2.4
6-CZGNR-(2,1)[45]	29 ± 2	−0.97	~18
6-CGNR-edge[46]	40 ± 3	−0.92	N/A
6-CGNR-cove	35 ± 2	−0.93	N/A
p-AGNR	18 ± 4	−0.88	N/A
9-AGNRs[47]	20 ± 5	−0.72	*352 ± 88*[e]
**Other materials**			
$MoTe_2$ (few-layer)[48]	11 ± 2	0 (Drude)	45 ± 9
Doped BP (nanosheets)[49]	88 ± 3	−0.88	97 ± 3
**2D conjugated and non-conjugated COFs measured by the TRMC rather than THz technique**			
COF-366[50]			8.1
COF-66			3.0
CS-COF[51]			4.2
H_2P-COF[52]			3.5
CuP-COF			0.19
ZnP-COF			0.032/0.016
NiPc COF[53]			1.3
HBC-COF[54]			0.7
2D-NiPc-BTDA COF[55]			0.6
TTF-Ph-COF[56]			0.2
TTF-Py-COF			0.08
AntTTH[57] ***film***			0.1
2D D-A COF[58]			0.01/0.04

[a]DFT and DFTB represent two typical methods to calculate energy band diagram.

[b]Reduced *m** of electron and hole.

[c]Estimated from literature.

[d]Averaged $m*$ of electron and hole.

[e]Calculated by $\mu = \frac{e\tau}{m^*}$ and c was neglected.

Section E. Supplementary References

1 Gaussian 16 Rev. C.01 (Wallingford, CT, 2016).

2 Grimme, S., Ehrlich, S. & Goerigk, L. Effect of the damping function in dispersion corrected density functional theory. *J. Comput. Chem.* **32**, 1456-1465, doi:https://doi.org/10.1002/jcc.21759 (2011).

3 Ochterski, J. W. Thermochemistry in Gaussian. Can be found under http://gaussian.com/thermo/ (2000).

4 Kresse, G. & Furthmüller, J. Efficiency of ab-initio total energy calculations for metals and semiconductors using a plane-wave basis set. *Comput. Mater. Sci.* **6**, 15-50, doi:https://doi.org/10.1016/0927-0256(96)00008-0 (1996).

5 Kresse, G. & Furthmüller, J. Efficient iterative schemes for ab initio total-energy calculations using a plane-wave basis set. *Phys. Rev. B* **54**, 11169-11186, doi:10.1103/PhysRevB.54.11169 (1996).

6 Blöchl, P. E. Projector augmented-wave method. *Phys. Rev. B* **50**, 17953-17979, doi:10.1103/PhysRevB.50.17953 (1994).

7 Kresse, G. & Joubert, D. From ultrasoft pseudopotentials to the projector augmented-wave method. *Phys. Rev. B* **59**, 1758-1775, doi:10.1103/PhysRevB.59.1758 (1999).

8 Perdew, J. P. *et al.* Atoms, molecules, solids, and surfaces: Applications of the generalized gradient approximation for exchange and correlation. *Phys. Rev. B* **46**, 6671-6687, doi:10.1103/PhysRevB.46.6671 (1992).

9 Perdew, J. P., Burke, K. & Ernzerhof, M. Generalized Gradient Approximation Made Simple. *Phys. Rev. Lett.* **77**, 3865-3868, doi:10.1103/PhysRevLett.77.3865 (1996).

10 Anisimov, V. I., Aryasetiawan, F. & Lichtenstein, A. I. First-principles calculations of the electronic structure and spectra of strongly correlated systems: theLDA+<i>U</i>method. *J. Phys. Condens. Matter* **9**, 767-808, doi:10.1088/0953-8984/9/4/002 (1997).

11 Li, W. *et al.* High temperature ferromagnetism in π-conjugated two-dimensional metal–organic frameworks. *Chem. Sci.* **8**, 2859-2867, doi:10.1039/C6SC05080H (2017).

12 Monkhorst, H. J. & Pack, J. D. Special points for Brillouin-zone integrations. *Phys. Rev. B* **13**, 5188-5192, doi:10.1103/PhysRevB.13.5188 (1976).

13 Grimme, S. Semiempirical GGA-type density functional constructed with a long-range dispersion correction. *J. Comput. Chem.* **27**, 1787-1799, doi:https://doi.org/10.1002/jcc.20495 (2006).

14 Wang, V., Xu, N., Liu, J.-C., Tang, G. & Geng, W.-T. VASPKIT: A user-friendly interface facilitating high-throughput computing and analysis using VASP code. *Comput. Phys. Commun.* **267**, 108033, doi:https://doi.org/10.1016/j.cpc.2021.108033 (2021).

15 Formalik, F., Fischer, M., Rogacka, J., Firlej, L. & Kuchta, B. Benchmarking of GGA density functionals for modeling structures of nanoporous, rigid and flexible MOFs. *J. Chem. Phys.* **149**, 064110, doi:10.1063/1.5030493 (2018).

16 M Ganose, A., J Jackson, A. & O Scanlon, D. sumo: Command-line tools for plotting

and analysis of periodic ab initio calculations. *J. Open Source Softw.* **3**, 717, doi:10.21105/joss.00717 (2018).

17 Wang, M. *et al.* Unveiling Electronic Properties in Metal–Phthalocyanine-Based Pyrazine-Linked Conjugated Two-Dimensional Covalent Organic Frameworks. *J. Am. Chem. Soc.* **141**, 16810-16816, doi:10.1021/jacs.9b07644 (2019).

18 Jiang, L. *et al.* A Crystalline Polyimide Porous Organic Framework for Selective Adsorption of Acetylene over Ethylene. *J. Am. Chem. Soc.* **140**, 15724-15730, doi:10.1021/jacs.8b08174 (2018).

19 Mamada, M., Pérez-Bolívar, C. & Anzenbacher, P. Green Synthesis of Polycyclic Benzimidazole Derivatives and Organic Semiconductors. *Org. Lett.* **13**, 4882-4885, doi:10.1021/ol201973w (2011).

20 Singh, D. & Baruah, J. B. Solvation controlling reaction paths and gel-formation in imide derivatives. *Tetrahedron Lett.* **49**, 4374-4377, doi:https://doi.org/10.1016/j.tetlet.2008.05.030 (2008).

21 Giannozzi, P. *et al.* QUANTUM ESPRESSO: a modular and open-source software project for quantum simulations of materials. *J. Phys. Condens. Matter* **21**, 395502, doi:10.1088/0953-8984/21/39/395502 (2009).

22 ACD/C+H NMR Predictors and DB 2017.1.3, Advanced Chemistry Development, Inc., Toronto, ON, Canada, www.acdlabs.com (2017).

23 Noh, H.-J. *et al.* Vertical two-dimensional layered fused aromatic ladder structure. *Nat. Commun.* **11**, 2021, doi:10.1038/s41467-020-16006-0 (2020).

24 Huang, D. *et al.* Conjugated-Backbone Effect of Organic Small Molecules for n-Type Thermoelectric Materials with ZT over 0.2. *J. Am. Chem. Soc.* **139**, 13013-13023, doi:10.1021/jacs.7b05344 (2017).

25 Lu, Y. *et al.* Rigid Coplanar Polymers for Stable n-Type Polymer Thermoelectrics. *Angew. Chem. Int. Ed.* **58**, 11390-11394, doi:https://doi.org/10.1002/anie.201905835 (2019).

26 Lakshmi, V. *et al.* A Two-Dimensional Poly(azatriangulene) Covalent Organic Framework with Semiconducting and Paramagnetic States. *J. Am. Chem. Soc.* **142**, 2155-2160, doi:10.1021/jacs.9b11528 (2020).

27 Yang, J. *et al.* Protonated Imine-Linked Covalent Organic Frameworks for Photocatalytic Hydrogen Evolution. *Angew. Chem. Int. Ed.* **60**, 19797-19803, doi:https://doi.org/10.1002/anie.202104870 (2021).

28 Yang, J. *et al.* Constitutional isomerism of the linkages in donor–acceptor covalent organic frameworks and its impact on photocatalysis. *Nat. Commun.* **13**, 6317, doi:10.1038/s41467-022-33875-9 (2022).

29 Khulbe, K. C., Ismail, A. F. & Matsuura, T. Chapter 3 - Electron Paramagnetic Resonance (EPR) Spectroscopy. *Membrane Characterization* 47-68, doi: https://doi.org/10.1016/B978-0-444-63776-5.00003-6 (2017).

30 Jin, E. *et al.* Module-Patterned Polymerization towards Crystalline 2D sp2-Carbon Covalent Organic Framework Semiconductors. *Angew. Chem. Int. Ed.* **61**, e202115020, doi:https://doi.org/10.1002/anie.202115020 (2022).

31 Liu, Y. *et al.* Vinylene-Linked 2D Conjugated Covalent Organic Frameworks by Wittig Reaction. *Angew. Chem. Int. Ed.* **134**, e202209762,

doi:https://doi.org/10.1002/anie.202209762 (2022).

32 Guo, J. *et al.* Conjugated organic framework with three-dimensionally ordered stable structure and delocalized π clouds. *Nat. Commun.* **4**, 2736, doi:10.1038/ncomms3736 (2013).

33 Fu, S. *et al.* Outstanding Charge Mobility by Band Transport in Two-Dimensional Semiconducting Covalent Organic Frameworks. *J. Am. Chem. Soc.* **144**, 7489-7496, doi:10.1021/jacs.2c02408 (2022).

34 Xing, G. *et al.* Nonplanar Rhombus and Kagome 2D Covalent Organic Frameworks from Distorted Aromatics for Electrical Conduction. *J. Am. Chem. Soc.* **144**, 5042-5050, doi:10.1021/jacs.1c13534 (2022).

35 Cocker, T. L. *et al.* Microscopic origin of the Drude-Smith model. *Phys. Rev. B* **96**, 205439, doi:10.1103/PhysRevB.96.205439 (2017).

36 Jin, E. *et al.* A Nanographene-Based Two-Dimensional Covalent Organic Framework as a Stable and Efficient Photocatalyst. *Angew. Chem. Int. Ed.* **61**, e202114059, doi:https://doi.org/10.1002/anie.202114059 (2022).

37 Wang, M. *et al.* High-Mobility Semiconducting Two-Dimensional Conjugated Covalent Organic Frameworks with p-Type Doping. *J. Am. Chem. Soc.* **142**, 21622-21627, doi:10.1021/jacs.0c10482 (2020).

38 Jin, E. *et al.* Exceptional electron conduction in two-dimensional covalent organic frameworks. *Chem* **7**, 3309-3324, doi:https://doi.org/10.1016/j.chempr.2021.08.015 (2021).

39 Yang, C. *et al.* A semiconducting layered metal-organic framework magnet. *Nat. Commun.* **10**, 3260, doi:10.1038/s41467-019-11267-w (2019).

40 Dong, R. *et al.* High-mobility band-like charge transport in a semiconducting two-dimensional metal–organic framework. *Nat. Mater.* **17**, 1027-1032, doi:10.1038/s41563-018-0189-z (2018).

41 Wang, Z.-Y. *et al.* Correlating Charge Transport Properties of Conjugated Polymers in Solution Aggregates and Thin-Film Aggregates. *Angew. Chem. Int. Ed.* **60**, 20483-20488, doi:https://doi.org/10.1002/anie.202107395 (2021).

42 Yao, X. *et al.* Synthesis of Nonplanar Graphene Nanoribbon with Fjord Edges. *J. Am. Chem. Soc.* **143**, 5654-5658, doi:10.1021/jacs.1c01882 (2021).

43 Niu, W. *et al.* A Curved Graphene Nanoribbon with Multi-Edge Structure and High Intrinsic Charge Carrier Mobility. *J. Am. Chem. Soc.* **142**, 18293-18298, doi:10.1021/jacs.0c07013 (2020).

44 Yang, L. *et al.* Solution Synthesis and Characterization of a Long and Curved Graphene Nanoribbon with Hybrid Cove–Armchair–Gulf Edge Structures. *Adv. Sci.* **9**, 2200708, doi:https://doi.org/10.1002/advs.202200708 (2022).

45 Wang, X. *et al.* Cove-Edged Graphene Nanoribbons with Incorporation of Periodic Zigzag-Edge Segments. *J. Am. Chem. Soc.* **144**, 228-235, doi:10.1021/jacs.1c09000 (2022).

46 Ivanov, I. *et al.* Role of Edge Engineering in Photoconductivity of Graphene Nanoribbons. *J. Am. Chem. Soc.* **139**, 7982-7988, doi:10.1021/jacs.7b03467 (2017).

47 Chen, Z. *et al.* Chemical Vapor Deposition Synthesis and Terahertz Photoconductivity of Low-Band-Gap N = 9 Armchair Graphene Nanoribbons. *J. Am. Chem. Soc.* **139**,

3635-3638, doi:10.1021/jacs.7b00776 (2017).
48 Zheng, W., Bonn, M. & Wang, H. I. Photoconductivity Multiplication in Semiconducting Few-Layer MoTe2. *Nano Lett.* **20**, 5807-5813, doi:10.1021/acs.nanolett.0c01693 (2020).
49 Shi, H. *et al.* Molecularly Engineered Black Phosphorus Heterostructures with Improved Ambient Stability and Enhanced Charge Carrier Mobility. *Adv. Mater.* **33**, 2105694, doi:https://doi.org/10.1002/adma.202105694 (2021).
50 Wan, S. *et al.* Covalent Organic Frameworks with High Charge Carrier Mobility. *Chem. Mater.* **23**, 4094-4097, doi:10.1021/cm201140r (2011).
51 Guo, J. *et al.* Conjugated organic framework with three-dimensionally ordered stable structure and delocalized pi clouds. *Nat. Commun.* **4**, 2736, doi:10.1038/ncomms3736 (2013).
52 Feng, X. *et al.* High-rate charge-carrier transport in porphyrin covalent organic frameworks: switching from hole to electron to ambipolar conduction. *Angew. Chem. Int. Ed.* **51**, 2618-2622, doi:10.1002/anie.201106203 (2012).
53 Ding, X. *et al.* Synthesis of metallophthalocyanine covalent organic frameworks that exhibit high carrier mobility and photoconductivity. *Angew. Chem. Int. Ed.* **50**, 1289-1293, doi:10.1002/anie.201005919 (2011).
54 Dalapati, S. *et al.* Rational design of crystalline supermicroporous covalent organic frameworks with triangular topologies. *Nat. Commun.* **6**, 7786, doi:10.1038/ncomms8786 (2015).
55 Ding, X. *et al.* An n-channel two-dimensional covalent organic framework. *J. Am. Chem. Soc.* **133**, 14510-14513, doi:10.1021/ja2052396 (2011).
56 Jin, S. *et al.* Two-dimensional tetrathiafulvalene covalent organic frameworks: towards latticed conductive organic salts. *Chem. Eur. J.* **20**, 14608-14613, doi:10.1002/chem.201402844 (2014).
57 Ghosh, S. *et al.* Band-like Transport of Charge Carriers in Oriented Two-Dimensional Conjugated Covalent Organic Frameworks. *Chem. Mater.* **34**, 736-745, doi:10.1021/acs.chemmater.1c03533 (2022).
58 Feng, X. *et al.* An Ambipolar Conducting Covalent Organic Framework with Self-Sorted and Periodic Electron Donor-Acceptor Ordering. *Adv. Mater.* **24**, 3026-3031, doi:doi:10.1002/adma.201201185 (2012).
